# Supplementary material for: Impact and cost-effectiveness of measles vaccination through microarray patches in 70 low-income and middle-income countries: mathematical modelling and early-stage economic evaluation
Source: BMJ Glob Health. 2023 Nov 10;8(11):e012204. doi: 10.1136/bmjgh-2023-012204 (PMC10649680; doi:10.1136/bmjgh-2023-012204)
Supplement: Supplementary data [file bmjgh-2023-012204supp001.pdf]

Online supplemental file S1. Supplementary methods and results.

For the manuscript entitled 'Impact and cost-effectiveness of measles vaccination through microarray patches in 70 low-income and middle-income countries: mathematical modelling and early-stage economic evaluation'

Han Fu[1, 2*], Kaja Abbas[1,2,3,4], Stefano Malvolti[5], Christopher Gregory[6], Melissa Ko[5], Jean-Pierre Amorij[7], Mark Jit[1,2,8]

[1]Department of Infectious Disease Epidemiology, London School of Hygiene & Tropical Medicine, London, United Kingdom

[2]Centre for Mathematical Modelling of Infectious Diseases, London School of Hygiene & Tropical Medicine, London, United Kingdom

[3]Public Health Foundation of India, New Delhi, India

[4]School of Tropical Medicine and Global Health, Nagasaki University, Nagasaki, Japan

[5]MMGH Consulting GmbH, Zurich, Switzerland

[6]Immunization Unit, Programme Division, United Nations Children's Fund (UNICEF), New York, United States

[7]Supply Division, Vaccine Centre, UNICEF, Copenhagen, Denmark

[8]School of Public Health, University of Hong Kong, Hong Kong, SAR, China

*Corresponding author: Han.Fu@lshtm.ac.uk

Contents

Text A. Estimating the theoretical price ranges for MR-MAPs 2
Text B. Cost of treating a measles case 4
Table A. List of 70 countries and related inputs included in the study 5
Table B. DynaMICE model parameters 7
Table C. Incremental cost (in million US$) of introducing MR-MAPs by income groups, coverage assumptions, and the MR-MAP prices 8
Table D. Number of countries where introducing MR-MAPs is cost-effective using differential discounting 8
Figure A. Health opportunity costs in 70 low- and middle-income countries. 9
Figure B. Net health benefits of introducing MR-MAPs in 70 countries using equal discounting 10

Text A. Estimating the theoretical price ranges for MR-MAPs

Utilising vaccine pricing data sources from the WHO Market Information for Access to Vaccines (MI4A) database (formerly known as VP3), United Nations Children's Fund (UNICEF) Supply Division price dashboard, and Pan American Health Organization Revolving Fund, a price benchmarking analysis for measles-rubella microarray patches (MR-MAPs) was conducted using data for MR vaccines considering single-dose vials (SDV) and pre-filled syringe (PFS) presentations procured by UNICEF for use in Gavi-eligible countries (low- and middle-income settings).[1-3] The identified prices for SDV and PFS presentations serve as the proxy for potential price ranges of MR-MAPs.

Currently, UNICEF procures only multi-dose vials (MDV) of MR for Gavi-eligible countries. To address this gap and to be able to estimate the potential price ranges of MR-MAPs as if procured by UNICEF for Gavi-eligible countries, a three-step approach was adopted.

Step 1: Identify non-MR vaccine price benchmarks and calculate the price differentials

All available data on vaccine prices were reviewed from the abovementioned data sources. Two criteria were applied when selecting the potential pricing data: (I) If it was possible to isolate the impact on price due to difference in presentation selected; and (ii) If different presentations (i.e., MDV, SDV, or PFS) were commercialised by one manufacturer in different markets (e.g., Gavi-eligible countries, self-procuring low and middle-income countries (LMICs)). Based on these criteria, pricing data for two vaccines were selected.

The monovalent paediatric Hepatitis B vaccine was selected as the most appropriate benchmark of MDV to SDV with an average difference between MDV and SDV prices of 122%. The paediatric Pneumococcal Conjugate vaccine was selected as the most appropriate benchmark of SDV to PFS with an average difference SDV and PFS of 110%. Note that these presentations are purchased by self-procuring countries.

Step 2: Apply the price differentials to calculate potential price ranges for MR in SDV and PFS presentations

First, the MR MDV (10-dose) price was converted to a SDV by applying the 122% price differential to the UNICEF price for Gavi-eligible countries of US$0.58 per dose and US$0.72 per dose. This estimated a MR SDV price range of US$1.29 to US$1.60 per dose for Gavi-eligible countries.

Next, the MR PFS price was estimated by applying the 110% price differential to the above calculated SDV price ranges resulting in an estimated MR PFS price range of US$2.70 to US$3.36 per dose in self-procuring LMICs.

Finally, these estimates were further validated by comparing measles-mumps-rubella (MMR) in SDV and PFS, and inactivated polio vaccine (IPV) in MDV and PFS, which indicated a general validity of the theoretical MR SDV and PFS prices.

Step 3: Calculate a potential price range for MR-MAPs for Gavi-supported countries

As the MR PFS price is for self-procuring LMICs, tiered pricing should be reflected, and it is important to consider Gavi-eligible countries procuring through UNICEF. Thus, we calculated the price differentials between self-procuring LMICs and UNICEF prices for Gavi-supported countries considering other vaccines. Four different vaccine products were utilised for this price differential, including: 2 pentavalent vaccines, 1 IPV vaccine and 1 MMR vaccine. The average price differential between self-procuring LMICs and Gavi-supported countries was 15%. Thus, the 15% price differential was applied, resulting in a *theoretical* MR PFS price range for Gavi-eligible countries between US$2.35 to US$2.92 per dose.

A similar methodology was employed to calculate estimated prices for middle-income countries. The table below provides an overview of the potential ranges of MR-MAP prices considering the country type. It should be noted that the actual market prices can vary based on many factors such as cost of goods sold, balance of demand and supply, specific country demand for one specific presentation. In this cost-effectiveness analysis, we took the lower SDV price and higher PFS price of each income group and used these proxy prices as the lower and upper MR-MAP prices.

Table: Potential ranges for MR-MAP prices by eligibility of Gavi support and income levels (US$)
Abbreviation: MR-MAP-measles-rubella microarray patch.

**Price comparators**		**Gavi-eligible or low income**	**Lower middle income (excluding Gavi-eligible)**	**Upper middle income**
**Single-dose vials (SDV)**	**Low**	1.29	1.48	2.63
	**High**	1.60	1.84	3.27
**Pre-filled syringe (PFS)**	**Low**	2.35	2.70	4.19
	**High**	2.92	3.36	5.20

Fu H, *et al. BMJ Glob Health* 2023; 8:e012204. doi: 10.1136/bmjgh-2023-012204

Text B. Cost of treating a measles case

To understand the cost of treating a measles case or an episode of measles disease in low- and middle-income countries (LMICs), we searched on PubMed with the search term combining the following criteria:

1. *measles*
2. *AND (((treatment OR medic* OR health*) AND cost) OR cost-of-illness)*
3. *NOT (Andorra OR Aruba OR Australia OR Austria OR Bahamas OR Belgium OR Bermuda OR Brunei Darussalam OR Canada OR Cayman Islands OR Channel Islands OR Cyprus OR Denmark OR Faeroe Islands OR Finland OR France OR French Polynesia OR Germany OR Greenland OR Guam OR Hong Kong OR Iceland OR Ireland OR Israel OR Italy OR Japan OR Korea Republic OR Kuwait OR Liechtenstein OR Luxembourg OR Macao OR Monaco OR Netherlands OR New Caledonia OR New Zealand OR Northern Mariana Islands OR Norway OR Portugal OR Qatar OR Singapore OR Spain OR Sweden OR Switzerland OR Taiwan OR United Arab Emirates OR United Kingdom OR United States OR Virgin Islands OR Netherlands Antilles).*

Adding a timeframe filter, we identified 322 articles published from 1st January 2000 to 30th September 2022. After screening and title, abstract, and full-text articles, we included 15 studies that provide cost estimates for measles treatment in LMICs. We further included 3 studies found through the reference lists of articles in the initial search collection.

We extracted a total of 35 estimates from 25 countries. All the cost estimates were converted to United States dollars[4] if needed and then inflated to the 2020 price using the Gross Domestic Product deflators.[5] We summarised the cost estimates by the World Bank income levels defined at the study years, with median and 25th–75th percentile: US$10.9 (6.47–19.2) in low-income countries,[6-12] US$120 (13.3–130) in lower-middle-income countries,[6, 7, 9, 13-17] and US$235 (134–295) in upper-middle-income countries.[6, 7, 18-23] The median cost of treating a measles case increases with income level. However, a large heterogeneity was observed across countries within income groups.

Table A. List of 70 countries and related inputs included in the study

Country-specific information on World Bank income level, Gavi eligibility, N&S type in use, introduction years and market penetration of MR-MAPs. The market penetration proportion of MR-MAPs was applied to replace traditional N&S doses used in national immunisation programmes, as the delivery components A–C described in Table 1 in the main text. For countries using mostly MMR in their immunisation programmes, the market penetration of MR-MAP is null. Abbreviations: MR-MAP-measles-rubella microarray patch, MMR-measles-mumps-rubella, N&S-needle and syringe.

				N&S type		Introduction year of MR-MAPs		
Income level	ISO code	Country	Gavi eligibility	Valent	Vial	Sequential plan	Accelerated plan	MR-MAPs market penetration
Low (n = 20)	AFG	Afghanistan	Yes	MR	5	2033	2033	0.8
	BFA	Burkina Faso	Yes	MR	5	2033	2030	0.8
	BDI	Burundi	Yes	MR	5	2033	2032	0.8
	TCD	Chad	Yes	MR	10	2032	2031	0.8
	COD	Democratic Republic of the Congo	Yes	MR	10	2032	2030	0.8
	ETH	Ethiopia	Yes	MR	5	2033	2033	0.8
	GMB	Gambia	Yes	MR	10	2035	2036	0.8
	LBR	Liberia	Yes	MR	5	2031	2030	0.8
	MDG	Madagascar	Yes	MR	5	2031	2031	0.8
	MWI	Malawi	Yes	MR	10	2032	2030	0.8
	MLI	Mali	Yes	MR	5	2033	2030	0.8
	MOZ	Mozambique	Yes	MR	10	2034	2031	0.8
	NER	Niger	Yes	MR	10	2031	2031	0.8
	SLE	Sierra Leone	Yes	MR	5	2037	2034	0.8
	SOM	Somalia	Yes	MR	5	2032	2032	0.8
	SDN	Sudan	Yes	MR	5	2033	2030	0.8
	SYR	Syria	Yes	MR	10	2039	2039	0.3
	TGO	Togo	Yes	MR	5	2037	2033	0.8
	UGA	Uganda	Yes	MR	10	2032	2030	0.8
	YEM	Yemen	Yes	MR	5	2031	2030	0.8
Lower middle (n = 35)	DZA	Algeria	No	MR	10	2032	2034	0.3
	AGO	Angola	No	MR	10	2033	2030	0.8
	BGD	Bangladesh	Yes	MR	5	2034	2034	0.8
	BEN	Benin	Yes	MR	10	2031	2033	0.8
	CMR	Cameroon	Yes	MR	5	2031	2032	0.8
	COM	Comoros	Yes	MR	5	2037	2037	0.8
	COG	Republic of Congo	Yes	MR	5	2032	2032	0.8
	CIV	Cote d'Ivoire	Yes	MR	10	2036	2031	0.8
	EGY	Egypt	No	MR	10	2038	2035	0.3
	SWZ	Eswatini	No	MR	5	2039	2037	0.8
	GHA	Ghana	Yes	MR	5	2032	2032	0.8
	IND	India	Yes	MR	5	2035	2030	0.8
	IDN	Indonesia	No	MR	10	2033	2030	0.8

	KEN	Kenya	Yes	MR	5	2036	2032	0.8
	KIR	Kiribati	No	MR	10	2040	2039	0.8
	LAO	Laos	Yes	MR	5	2033	2031	0.8
	LSO	Lesotho	Yes	MR	5	2038	2034	0.8
	MRT	Mauritania	Yes	MR	10	2038	2033	0.8
	FSM	Federated States of Micronesia	No	MMR	2	2038	2036	0
	MMR	Myanmar	Yes	MR	5	2036	2035	0.8
	NPL	Nepal	Yes	MR	10	2037	2033	0.8
	NGA	Nigeria	Yes	MR	5	2034	2031	0.8
	PAK	Pakistan	Yes	MR	10	2033	2031	0.8
	PNG	Papua New Guinea	Yes	MR	5	2036	2033	0.8
	PHL	Philippines	No	MR	5	2030	2030	0.8
	WSM	Samoa	No	MR	5	2037	2038	0.3
	STP	Sao Tome and Principe	Yes	MR	5	2040	2039	0.8
	SEN	Senegal	Yes	MR	5	2035	2031	0.8
	SLB	Solomon Islands	Yes	MR	5	2039	2038	0.8
	TZA	Tanzania	Yes	MR	5	2031	2032	0.8
	TUN	Tunisia	No	MR	10	2033	2033	0.8
	UKR	Ukraine	No	MMR	2	2038	2034	0
	VNM	Viet Nam	No	MR	10	2035	2032	0.8
	ZMB	Zambia	Yes	MR	5	2033	2032	0.8
	ZWE	Zimbabwe	Yes	MR	10	2037	2034	0.8
**Upper middle** (n = 15)	BIH	Bosnia and Herzegovina	No	MMR	1	2032	2031	0
	BRA	Brazil	No	MMR	1	2036	2037	0
	CHN	China	No	MMR	1	2030	2033	0
	GNQ	Equatorial Guinea	No	MR	10	2035	2036	0.8
	GAB	Gabon	No	MR	10	2035	2036	0.8
	IRQ	Iraq	No	MMR	10	2030	2031	0
	LBN	Lebanon	No	MMR	1	2034	2034	0
	LBY	Libya	No	MMR	1	2036	2035	0
	NAM	Namibia	No	MR	10	2037	2039	0.8
	ROU	Romania	No	MMR	1	2034	2035	0
	RUS	Russian Federation	No	MMR	1	2036	2035	0
	SRB	Serbia	No	MMR	1	2040	2038	0
	ZAF	South Africa	No	MR	10	2030	2030	0.8
	TUR	Turkey	No	MMR	1	2030	2031	0
	VEN	Venezuela	No	MMR	5	2036	2038	0

Table B. DynaMICE model parameters

Key parameters for measles transmission and vaccination used in the DynaMICE model are listed, adapted from Fu et al.[24] Abbreviations: DynaMICE- dynamic model of immunization calculation engine. SIA- supplementary immunisation activity.

Parameters	Value	References and notes
Age-dependent contact matrix between a susceptible and an infectious case	country-specific	Prem et al.[25]
Basic reproduction number	15.9	Guerra et al.[26]
Measles recovering rate	1/14 d^{-1}	Strebel et al.[27]
Maternal immunity waning rate	1/0.5 y^{-1}	Assumed to last for an average of 6 months
Amplification factor for seasonality	0.05	Assumed
Two-dose vaccine efficacy	0.98	Sudfeld, Navar, and Halsey[28]
Age-dependent first-dose vaccine efficacy	0.64598+0.01485* age in months	Hughes et al.[29] Determined by a linear function and capped at 98%
Proportion of children with zero MCV dose (unvaccinated)	0.077	Cata-Preta et al.[30] Used to allocate SIA doses by vaccination states in the target population

Table C. Incremental cost (in million US$) of introducing MR-MAPs by income groups, coverage assumptions, and the MR-MAP prices

Numbers represent the total undiscounted incremental costs by income groups, coverage assumptions, and MR-MAP prices. Negative values indicate savings following the introduction of MR-MAPs, in consideration of costs for vaccine procurement, vaccine delivery, and measles treatment. Abbreviations: MR-MAP-measles-rubella microarray patch.

Projection assumption	Higher coverage				Lower coverage			
Introduction strategy	Sequential		Accelerated		Sequential		Accelerated	
MR-MAP price	Lower	Upper	Lower	Upper	Lower	Upper	Lower	Upper
Low income	390	1167	455	1348	181	827	222	970
Lower middle income	453	1986	787	2912	-1140	208	-1531	359
Upper middle income	41.3	176	2.08	124	-75.7	45.8	-55.2	53.9
Total	884	3329	1244	4384	-1034	1081	-1364	1383

Table D. Number of countries where introducing MR-MAPs is cost-effective using differential discounting

Introducing MR-MAPs is considered cost-effective if a country-specific ICER of introduction is below its country-specific threshold (Figure A in online supplemental file S1). Differential discounting that assumes 3% of annual discount rate on costs and 0% on health outcomes was applied. Abbreviations: ICER-incremental cost-effectiveness ratio, MR-MAP-measles-rubella microarray patch.

Projection assumption	Higher coverage				Lower coverage			
Introduction strategy	Sequential		Accelerated		Sequential		Accelerated	
MR-MAP price	Lower	Upper	Lower	Upper	Lower	Upper	Lower	Upper
Low income	5/20	1/20	6/20	2/20	18/20	14/20	18/20	15/20
Lower middle income	10/35	7/35	10/35	7/35	26/35	22/35	28/35	24/35
Upper middle income	6/15	5/15	7/15	7/15	11/15	11/15	11/15	11/15
Total	21/70	13/70	23/70	16/70	55/70	47/70	57/70	50/70

Figure A. Health opportunity costs in 70 low- and middle-income countries.
Circles denote country-level health opportunity costs extracted from and Ochalek et al.[31] and used as cost-effectiveness thresholds in this study. Horizontal bars denote the income-level health opportunity costs weighted by population size in 2020.[32]

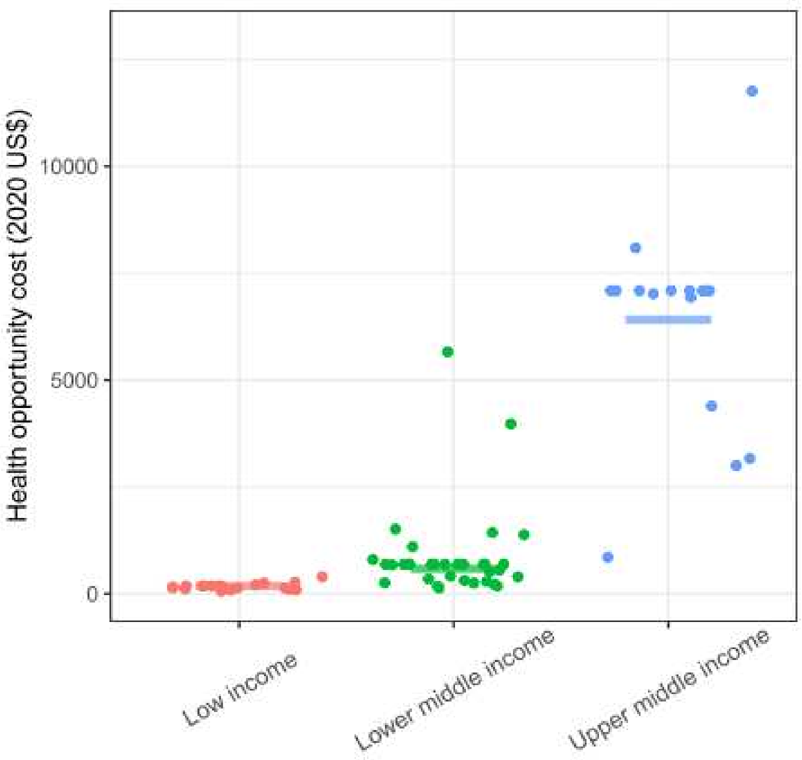

Figure B. Net health benefits of introducing MR-MAPs in 70 countries using equal discounting
To visualise the cost-effectiveness of MR-MAPs introduction for individual countries with different health opportunity costs, we calculated the net health benefits by subtracting the number of averted DALYs by the ratio of incremental cost to the country-specific health opportunity costs per DALY. Data points denote the net health benefits under equal discounting rates at the country level, presented by the assumptions of higher (panel A) and lower (panel B) coverage projection, sequential (green) and accelerated (red) introduction strategies, and lower (circles) and upper (triangles) MR-MAP prices. Values above zero, denoted by the horizontal grey dashed lines, indicate the introduction of MR-MAPs to be cost-effective based on country-specific thresholds. Abbreviations: DALY-disability-adjusted life year, MR-MAP-measles-rubella microarray patch.

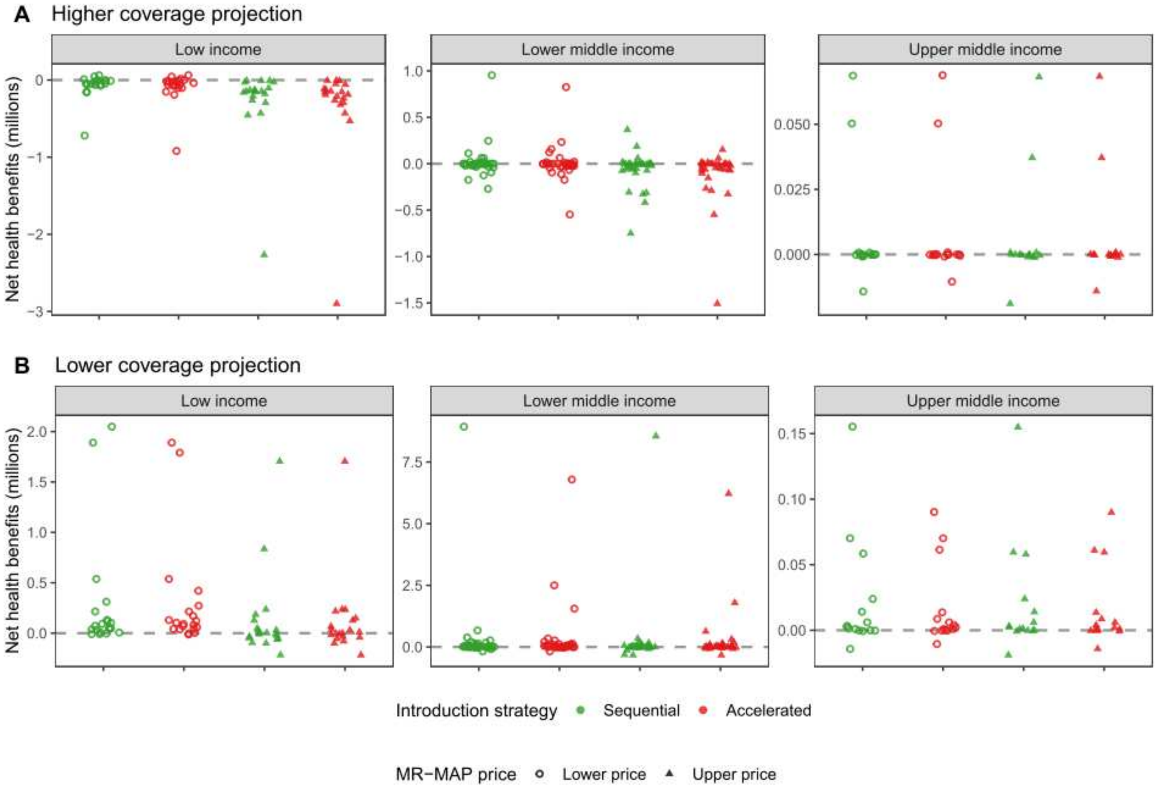

Fu H, *et al. BMJ Glob Health* 2023; 8:e012204. doi: 10.1136/bmjgh-2023-012204

References

1. Gavi, the Vaccine Alliance. Measles and rubella vaccine (MR) price data. 2021.
2. World Health Organization. MI4A Vaccine Purchase Database. [dataset] https://cdn.who.int/media/docs/default-source/immunization/mi4a/mi4a_public_database.xlsx?sfvrsn=1adc791a_11. Accessed 6 December 2021.
3. Pan American Health Organization. PAHO Revolving Fund Vaccine Prices for 2020. 2020.
4. The World Bank. Official exchange rate (LCU per US$, period average). [dataset] https://data.worldbank.org/indicator/PA.NUS.FCRF. Accessed 20 October 2022.
5. The World Bank. GDP deflator (base year varies by country). [dataset] https://data.worldbank.org/indicator/NY.GDP.DEFL.ZS. Accessed 30 November 2021.
6. Acharya A, Diaz-Ortega JL, Tambini G, et al. Cost-effectiveness of measles elimination in Latin America and the Caribbean: a prospective analysis. Vaccine. 2002;20(27-28):3332-41. doi: 10.1016/s0264-410x(02)00296-7
7. Levin A, Burgess C, Garrison LP, Jr., et al. Global eradication of measles: an epidemiologic and economic evaluation. J Infect Dis. 2011;204 Suppl 1:S98-106. doi: 10.1093/infdis/jir096
8. Dayan GH, Cairns L, Sangrujee N, et al. Cost-effectiveness of three different vaccination strategies against measles in Zambian children. Vaccine. 2004;22(3-4):475-84. doi: 10.1016/j.vaccine.2003.07.007
9. Stack ML, Ozawa S, Bishai DM, et al. Estimated economic benefits during the 'decade of vaccines' include treatment savings, gains in labor productivity. Health Aff (Millwood). 2011;30(6):1021-8. doi: 10.1377/hlthaff.2011.0382
10. Wallace AS, Masresha BG, Grant G, et al. Evaluation of economic costs of a measles outbreak and outbreak response activities in Keffa Zone, Ethiopia. Vaccine. 2014;32(35):4505-14. doi: 10.1016/j.vaccine.2014.06.035
11. Doshi RH, Eckhoff P, Cheng A, et al. Assessing the cost-effectiveness of different measles vaccination strategies for children in the Democratic Republic of Congo. Vaccine. 2017;35(45):6187-94. doi: 10.1016/j.vaccine.2017.09.038
12. Sodjinou VD, Nimpa MM, Douba A, et al. Challenges related to resources mobilization for measles outbreak response: Madagascar experience during the 2018-2019 measles outbreak. Pan Afr Med J. 2020;36:304. doi: 10.11604/pamj.2020.36.304.24514
13. Zhang SJ, Wang CF, Qi YY, et al. Clinical observation on treatment of 32 patients with measles by qingzhen decoction. Chin J Integr Med. 2009;15(5):389-92. doi: 10.1007/s11655-009-0389-0
14. Pike J, Tippins A, Nyaku M, et al. Cost of a measles outbreak in a remote island economy: 2014 Federated States of Micronesia measles outbreak. Vaccine. 2017;35(43):5905-11. doi: 10.1016/j.vaccine.2017.08.075
15. Megiddo I, Colson AR, Nandi A, et al. Analysis of the Universal Immunization Programme and introduction of a rotavirus vaccine in India with IndiaSim. Vaccine. 2014;32 Suppl 1:A151-61. doi: 10.1016/j.vaccine.2014.04.080
16. Bettampadi D, Boulton ML, Power LE, et al. Are community health workers cost-effective for childhood vaccination in India? Vaccine. 2019;37(22):2942-51. doi: 10.1016/j.vaccine.2019.04.038
17. de Broucker G, Ahmed S, Hasan MZ, et al. The economic burden of measles in children under five in Bangladesh. BMC Health Serv Res. 2020;20(1):1026. doi: 10.1186/s12913-020-05880-5
18. Uzicanin A, Zhou F, Eggers R, et al. Economic analysis of the 1996-1997 mass measles immunization campaigns in South Africa. Vaccine. 2004;22(25-26):3419-26. doi: 10.1016/j.vaccine.2004.02.042
19. Njau J, Janta D, Stanescu A, et al. Assessment of Economic Burden of Concurrent Measles and Rubella Outbreaks, Romania, 2011-2012. Emerging infectious diseases. 2019;25(6):1101-9. doi: 10.3201/eid2506.180339
20. Ma R, Lu L, Suo L, et al. An expensive adult measles outbreak and response in office buildings during the era of accelerated measles elimination, Beijing, China. Vaccine. 2017;35(8):1117-23. doi: 10.1016/j.vaccine.2017.01.021
21. Deng X, He H, Zhou Y, et al. Economic burden and associated factors of measles patients in Zhejiang Province, China. Hum Vaccin Immunother. 2019;15(11):2571-7. doi: 10.1080/21645515.2019.1599673

22. Zhou R, Li L, Yuan S, et al. Health and economic costs of an import-initiated measles outbreak in an international border area of Yunnan Province. Hum Vaccin Immunother. 2021;17(5):1347-52. doi: 10.1080/21645515.2020.1815488
23. Chen J, Jia H, Cai Z, et al. Economic burden of measles and its influencing factors in Fujian, China. Hum Vaccin Immunother. 2021;17(12):5367-71. doi: 10.1080/21645515.2021.1989917
24. Fu H, Abbas K, Klepac P, et al. Effect of evidence updates on key determinants of measles vaccination impact: a DynaMICE modelling study in ten high-burden countries. BMC Med. 2021;19(1):281. doi: 10.1186/s12916-021-02157-4
25. Prem K, van Zandvoort K, Klepac P, et al. Projecting contact matrices in 177 geographical regions: an update and comparison with empirical data for the COVID-19 era. Plos Comput Bio. 2021;17(7):e1009098. doi: 10.1371/journal.pcbi.1009098
26. Guerra FM, Bolotin S, Lim G, et al. The basic reproduction number (R0) of measles: a systematic review. Lancet Infect Dis. 2017;17(12):e420-e8. doi: 10.1016/S1473-3099(17)30307-9
27. Strebel PM, Papania MJ, Gastañaduy PA, et al. 37 - Measles Vaccines. In: Plotkin SA, Orenstein WA, Offit PA, Edwards KM, editors. Plotkin's Vaccines (Seventh Edition): Elsevier; 2018. p. 579-618.e21.
28. Sudfeld CR, Navar AM, Halsey NA. Effectiveness of measles vaccination and vitamin A treatment. Int J Epidemiol. 2010;39 Suppl 1:i48-55. doi: 10.1093/ije/dyq021
29. Hughes SL, Bolotin S, Khan S, et al. The effect of time since measles vaccination and age at first dose on measles vaccine effectiveness - A systematic review. Vaccine. 2020;38(3):460-9. doi: 10.1016/j.vaccine.2019.10.090
30. Cata-Preta BO, Santos TM, Mengistu T, et al. Zero-dose children and the immunisation cascade: Understanding immunisation pathways in low and middle-income countries. Vaccine. 2021;39(32):4564-70. doi: 10.1016/j.vaccine.2021.02.072
31. Ochalek J, Lomas J. Reflecting the Health Opportunity Costs of Funding Decisions Within Value Frameworks: Initial Estimates and the Need for Further Research. Clin Ther. 2020;42(1):44-59 e2. doi: 10.1016/j.clinthera.2019.12.002
32. United Nations Deparatment of Economic and Social Affairs. World Population Prospects. [dataset] 2019. https://population.un.org/wpp/. Accessed 1 March 2020.
