# Supplementary material for: Impact and cost-effectiveness of measles vaccination through microarray patches in 70 low-income and middle-income countries: mathematical modelling and early-stage economic evaluation
Source: BMJ Glob Health. 2023 Nov 10;8(11):e012204. doi: 10.1136/bmjgh-2023-012204 (PMC10649680; doi:10.1136/bmjgh-2023-012204)
Supplement: Supplementary data [file bmjgh-2023-012204supp002.pdf]

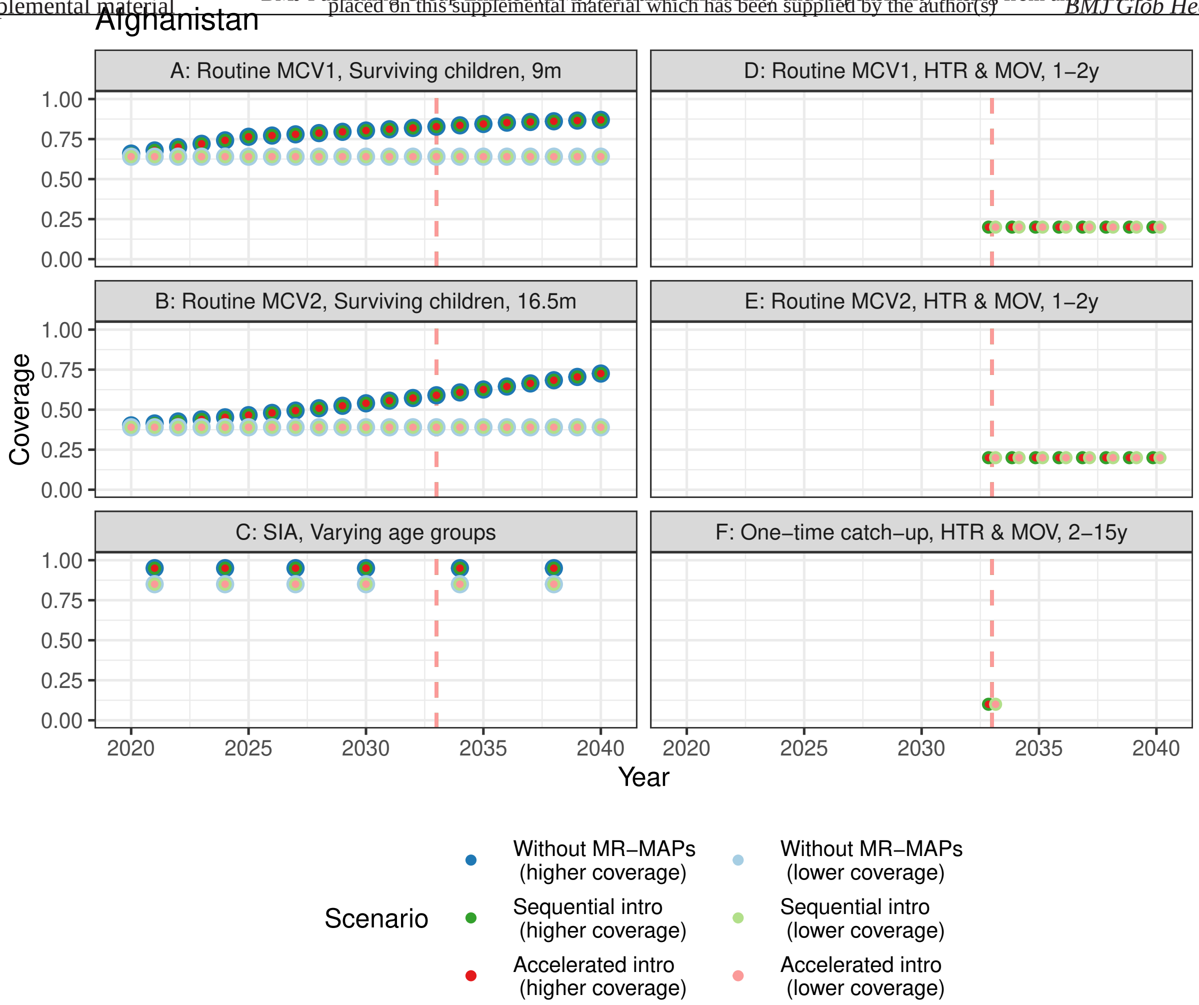
Afghanistan
A: Routine MCV1, Surviving children, 9m
D: Routine MCV1, HTR & MOV, 1−2y
B: Routine MCV2, Surviving children, 16.5m
E: Routine MCV2, HTR & MOV, 1−2y
C: SIA, Varying age groups
F: One−time catch−up, HTR & MOV, 2−15y
Coverage
1.00
0.75
0.50
0.25
0.00
2020
2025
2030
2035
2040
Year
Scenario
Without MR−MAPs (higher coverage)
Sequential intro (higher coverage)
Accelerated intro (higher coverage)
Without MR−MAPs (lower coverage)
Sequential intro (lower coverage)
Accelerated intro (lower coverage)

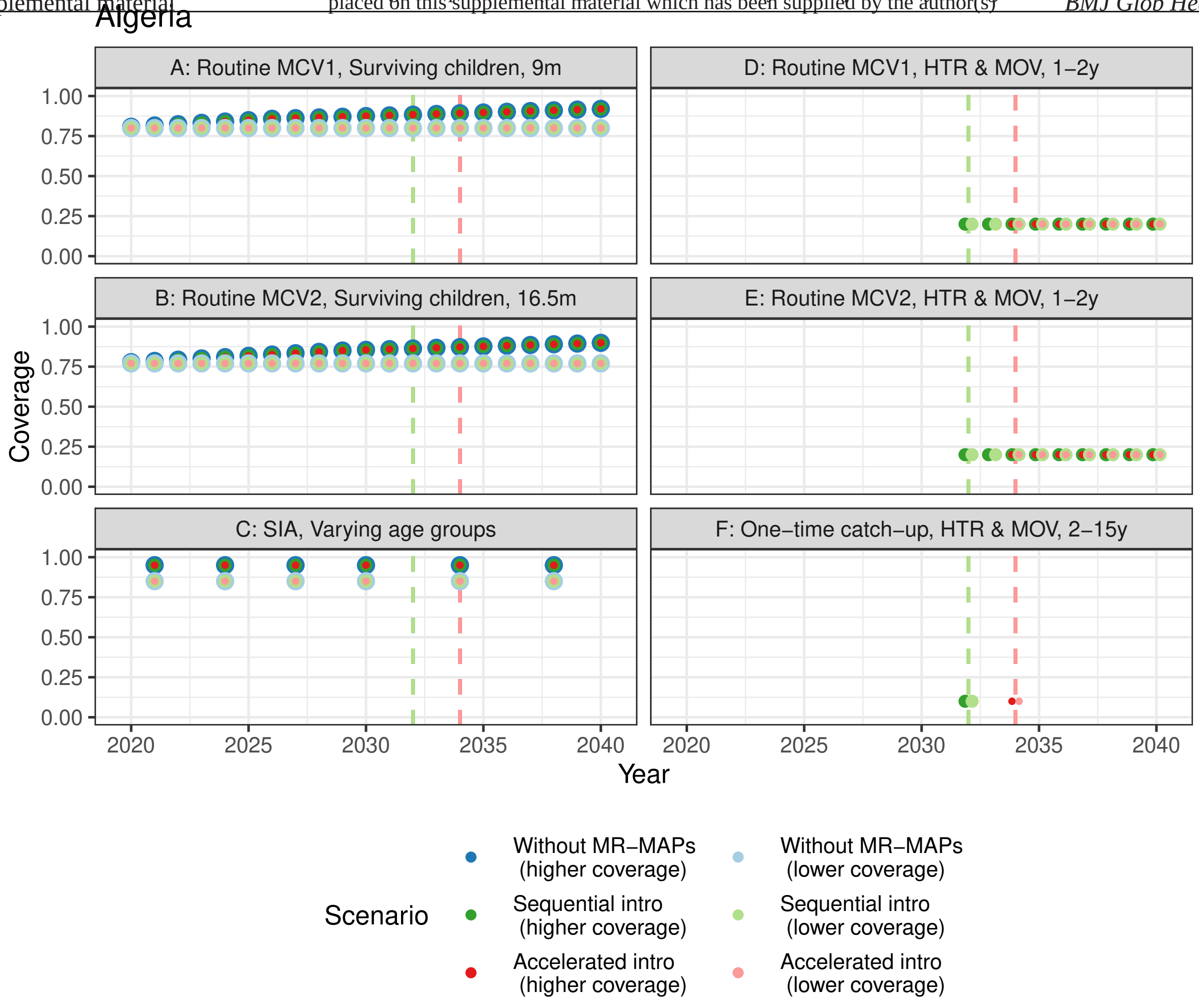

Algeria
A: Routine MCV1, Surviving children, 9m
D: Routine MCV1, HTR & MOV, 1−2y
B: Routine MCV2, Surviving children, 16.5m
E: Routine MCV2, HTR & MOV, 1−2y
C: SIA, Varying age groups
F: One−time catch−up, HTR & MOV, 2−15y
Coverage
1.00
0.75
0.50
0.25
0.00
2020
2025
2030
2035
2040
Year
Scenario
Without MR−MAPs (higher coverage)
Without MR−MAPs (lower coverage)
Sequential intro (higher coverage)
Sequential intro (lower coverage)
Accelerated intro (higher coverage)
Accelerated intro (lower coverage)

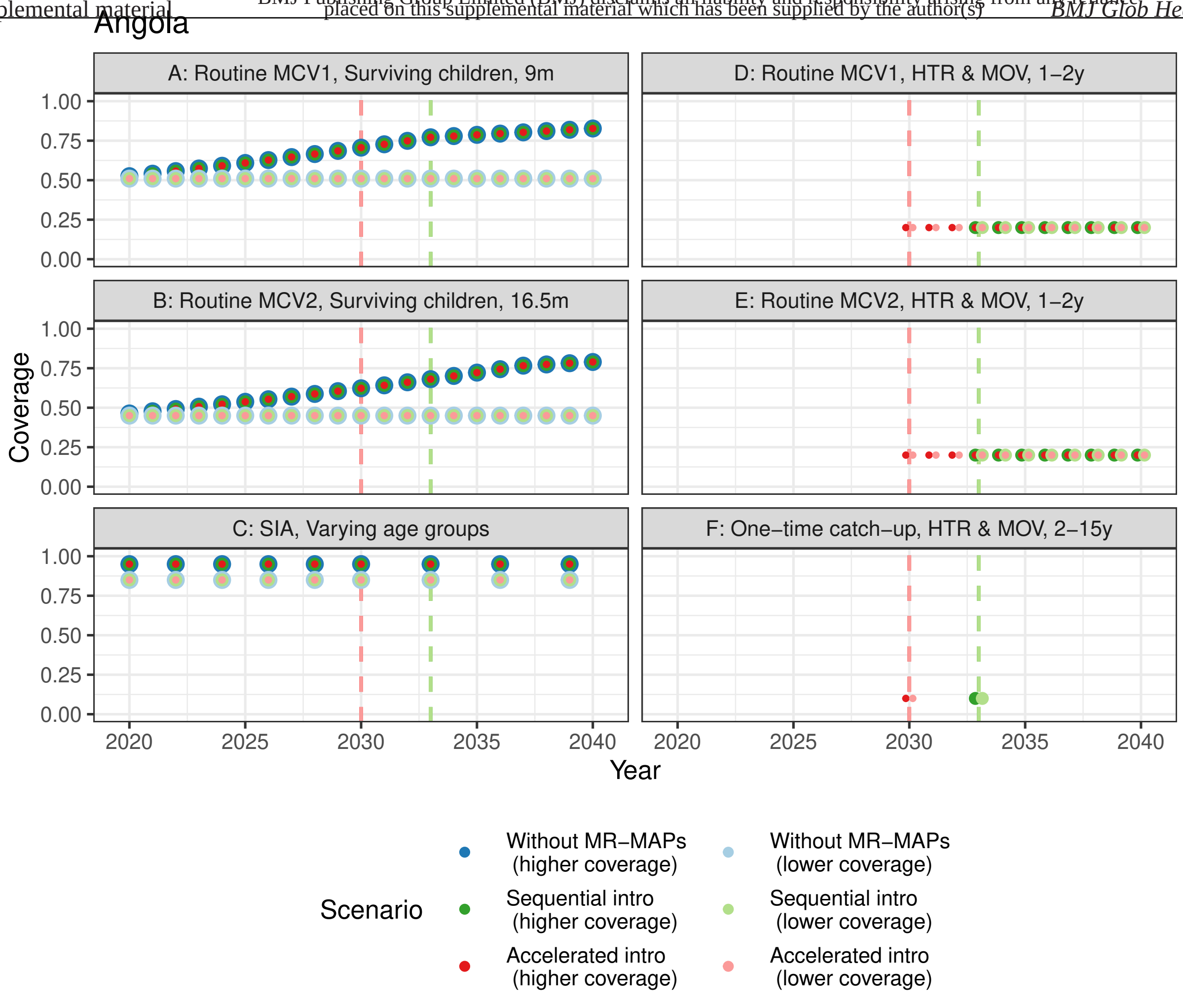
Angola
A: Routine MCV1, Surviving children, 9m
B: Routine MCV2, Surviving children, 16.5m
C: SIA, Varying age groups
D: Routine MCV1, HTR & MOV, 1−2y
E: Routine MCV2, HTR & MOV, 1−2y
F: One−time catch−up, HTR & MOV, 2−15y
Coverage
1.00
0.75
0.50
0.25
0.00
2020
2025
2030
2035
2040
Year
Scenario
Without MR−MAPs (higher coverage)
Sequential intro (higher coverage)
Accelerated intro (higher coverage)
Without MR−MAPs (lower coverage)
Sequential intro (lower coverage)
Accelerated intro (lower coverage)

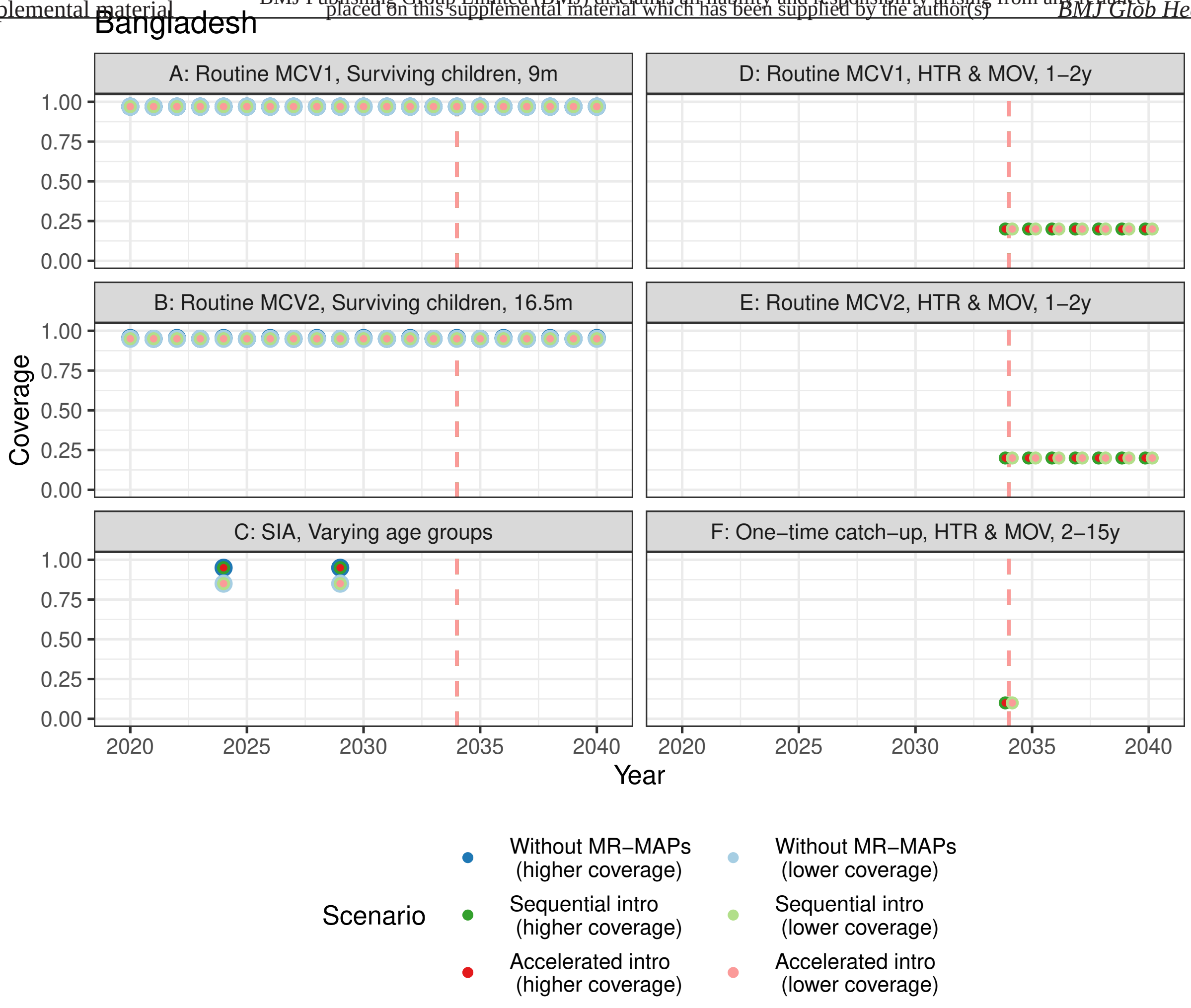
Bangladesh
A: Routine MCV1, Surviving children, 9m
D: Routine MCV1, HTR & MOV, 1–2y
B: Routine MCV2, Surviving children, 16.5m
E: Routine MCV2, HTR & MOV, 1–2y
C: SIA, Varying age groups
F: One–time catch–up, HTR & MOV, 2–15y
Coverage
1.00
0.75
0.50
0.25
0.00
2020
2025
2030
2035
2040
Year
Scenario
Without MR–MAPs (higher coverage)
Sequential intro (higher coverage)
Accelerated intro (higher coverage)
Without MR–MAPs (lower coverage)
Sequential intro (lower coverage)
Accelerated intro (lower coverage)

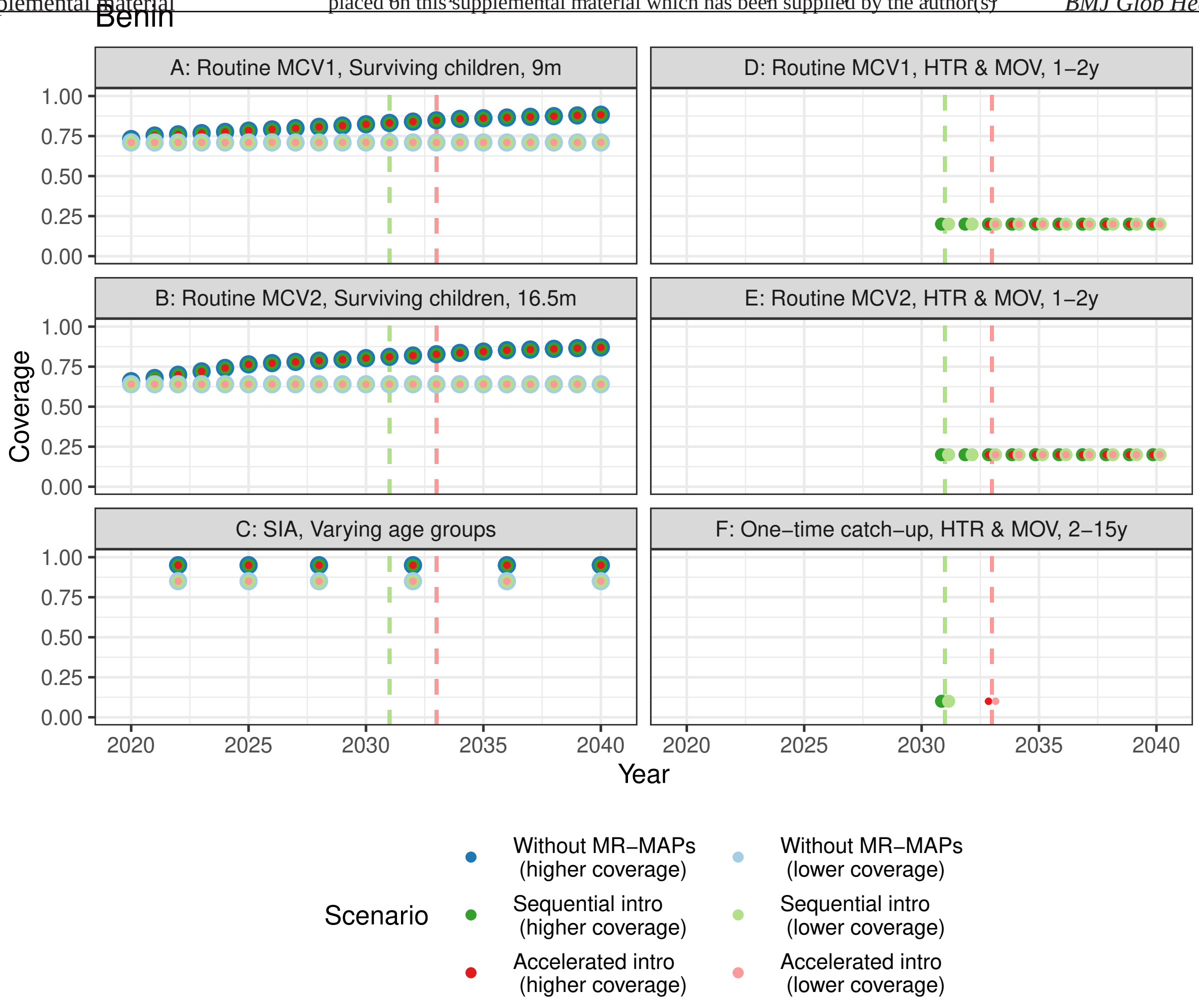
Benin
A: Routine MCV1, Surviving children, 9m
D: Routine MCV1, HTR & MOV, 1–2y
B: Routine MCV2, Surviving children, 16.5m
E: Routine MCV2, HTR & MOV, 1–2y
C: SIA, Varying age groups
F: One–time catch–up, HTR & MOV, 2–15y
Coverage
1.00
0.75
0.50
0.25
0.00
2020
2025
2030
2035
2040
Year
Scenario
Without MR–MAPs (higher coverage)
Sequential intro (higher coverage)
Accelerated intro (higher coverage)
Without MR–MAPs (lower coverage)
Sequential intro (lower coverage)
Accelerated intro (lower coverage)

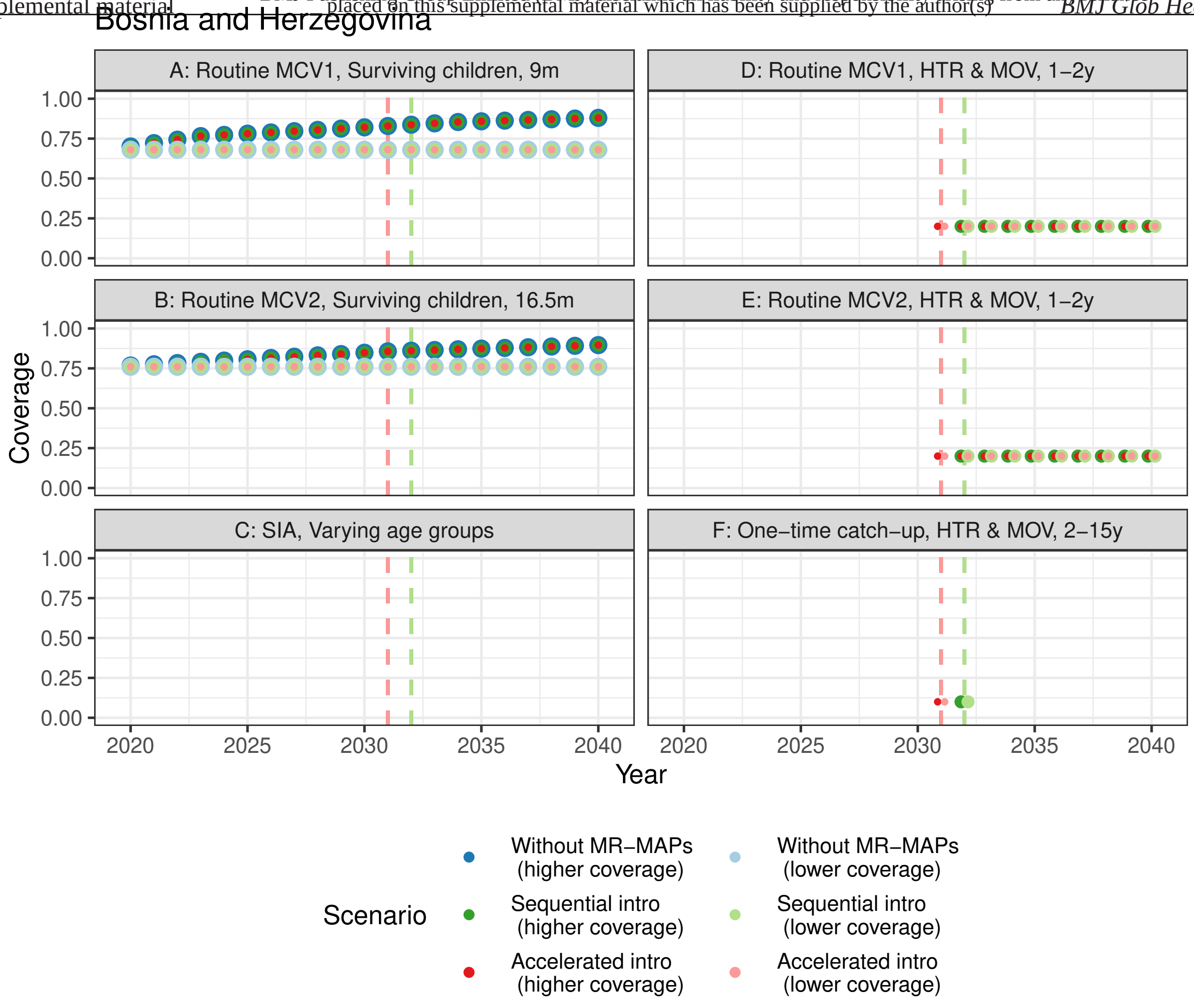
Bosnia and Herzegovina
A: Routine MCV1, Surviving children, 9m
D: Routine MCV1, HTR & MOV, 1−2y
B: Routine MCV2, Surviving children, 16.5m
E: Routine MCV2, HTR & MOV, 1−2y
C: SIA, Varying age groups
F: One−time catch−up, HTR & MOV, 2−15y
Coverage
1.00
0.75
0.50
0.25
0.00
2020
2025
2030
2035
2040
Year
Scenario
Without MR−MAPs (higher coverage)
Without MR−MAPs (lower coverage)
Sequential intro (higher coverage)
Sequential intro (lower coverage)
Accelerated intro (higher coverage)
Accelerated intro (lower coverage)

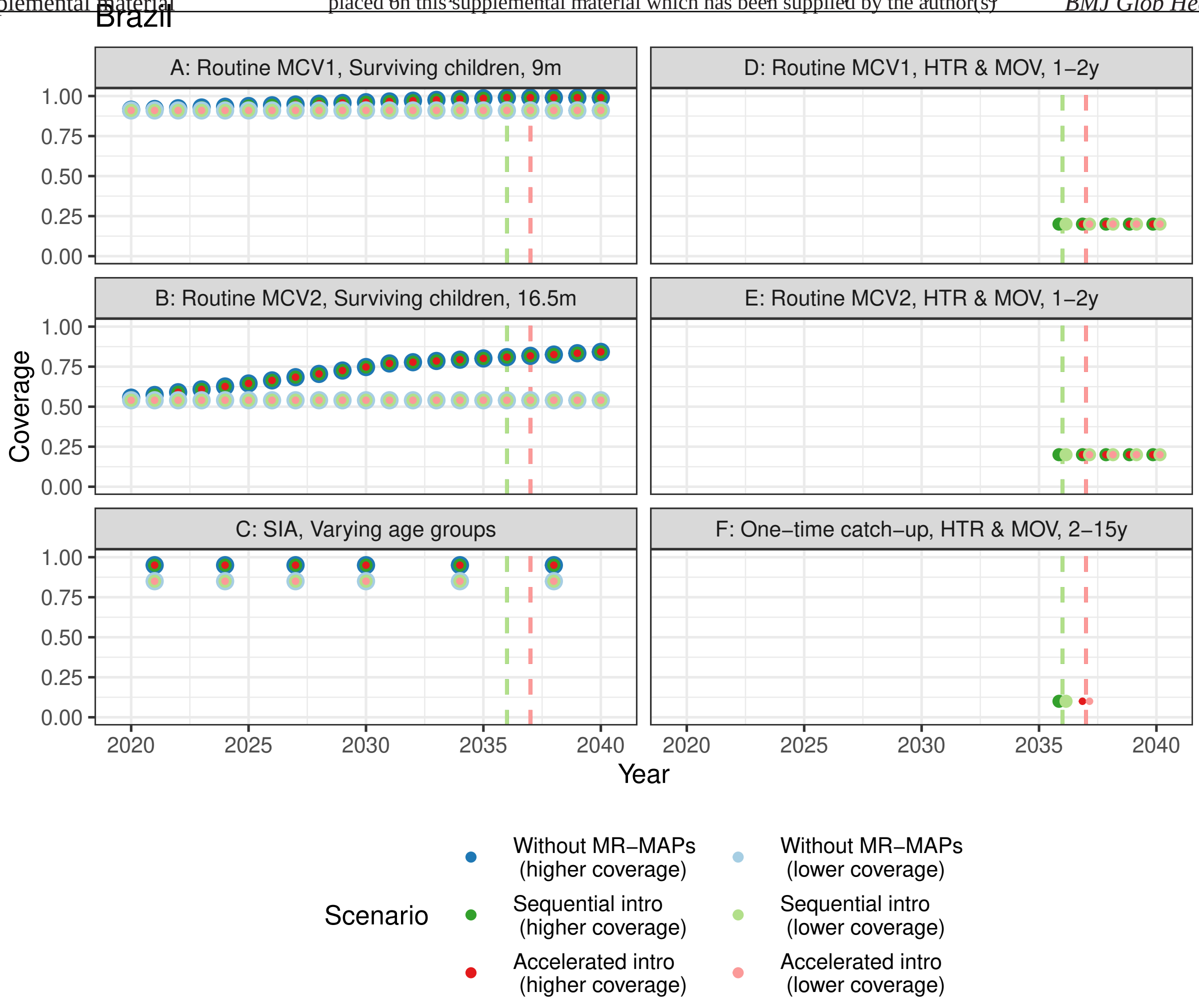
Brazil
A: Routine MCV1, Surviving children, 9m
D: Routine MCV1, HTR & MOV, 1–2y
B: Routine MCV2, Surviving children, 16.5m
E: Routine MCV2, HTR & MOV, 1–2y
C: SIA, Varying age groups
F: One–time catch–up, HTR & MOV, 2–15y
Coverage
1.00
0.75
0.50
0.25
0.00
2020
2025
2030
2035
2040
Year
Scenario
Without MR–MAPs (higher coverage)
Sequential intro (higher coverage)
Accelerated intro (higher coverage)
Without MR–MAPs (lower coverage)
Sequential intro (lower coverage)
Accelerated intro (lower coverage)

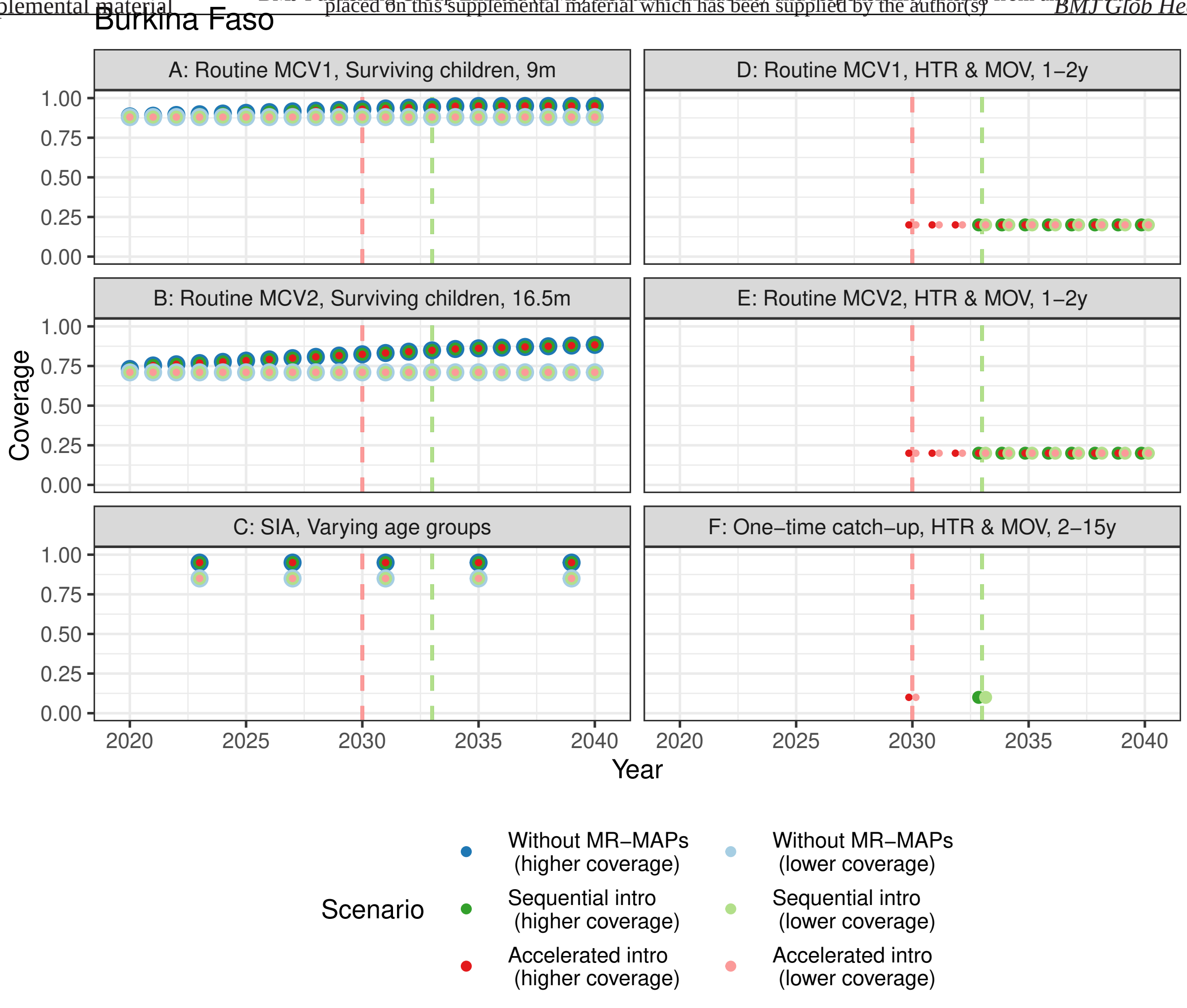
Burkina Faso
A: Routine MCV1, Surviving children, 9m
D: Routine MCV1, HTR & MOV, 1−2y
B: Routine MCV2, Surviving children, 16.5m
E: Routine MCV2, HTR & MOV, 1−2y
C: SIA, Varying age groups
F: One−time catch−up, HTR & MOV, 2−15y
Coverage
1.00
0.75
0.50
0.25
0.00
Year
2020
2025
2030
2035
2040
Scenario
Without MR−MAPs (higher coverage)
Sequential intro (higher coverage)
Accelerated intro (higher coverage)
Without MR−MAPs (lower coverage)
Sequential intro (lower coverage)
Accelerated intro (lower coverage)

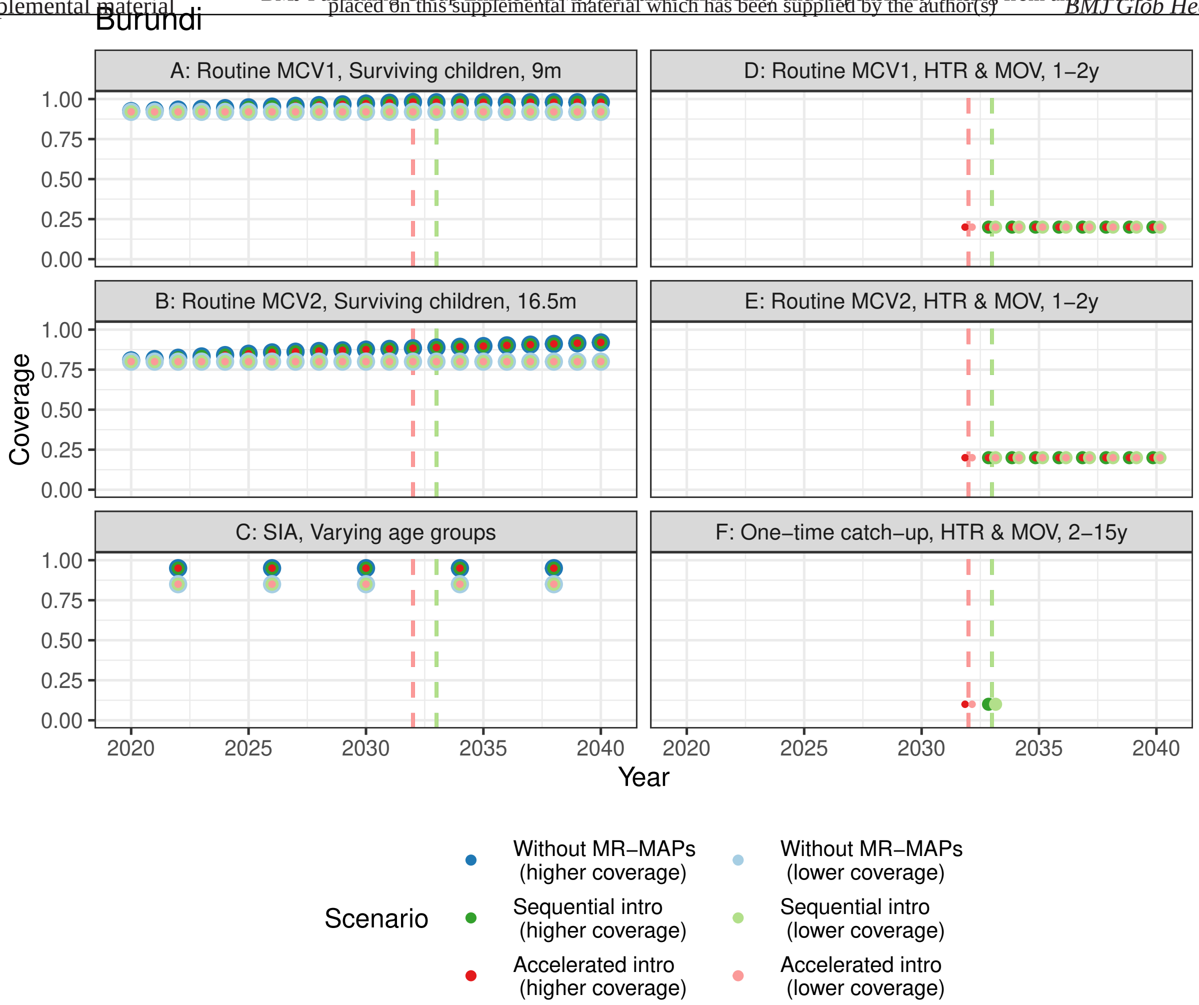
Burundi
A: Routine MCV1, Surviving children, 9m
D: Routine MCV1, HTR & MOV, 1−2y
B: Routine MCV2, Surviving children, 16.5m
E: Routine MCV2, HTR & MOV, 1−2y
C: SIA, Varying age groups
F: One−time catch−up, HTR & MOV, 2−15y
Coverage
1.00
0.75
0.50
0.25
0.00
2020
2025
2030
2035
2040
Year
Scenario
Without MR−MAPs (higher coverage)
Without MR−MAPs (lower coverage)
Sequential intro (higher coverage)
Sequential intro (lower coverage)
Accelerated intro (higher coverage)
Accelerated intro (lower coverage)

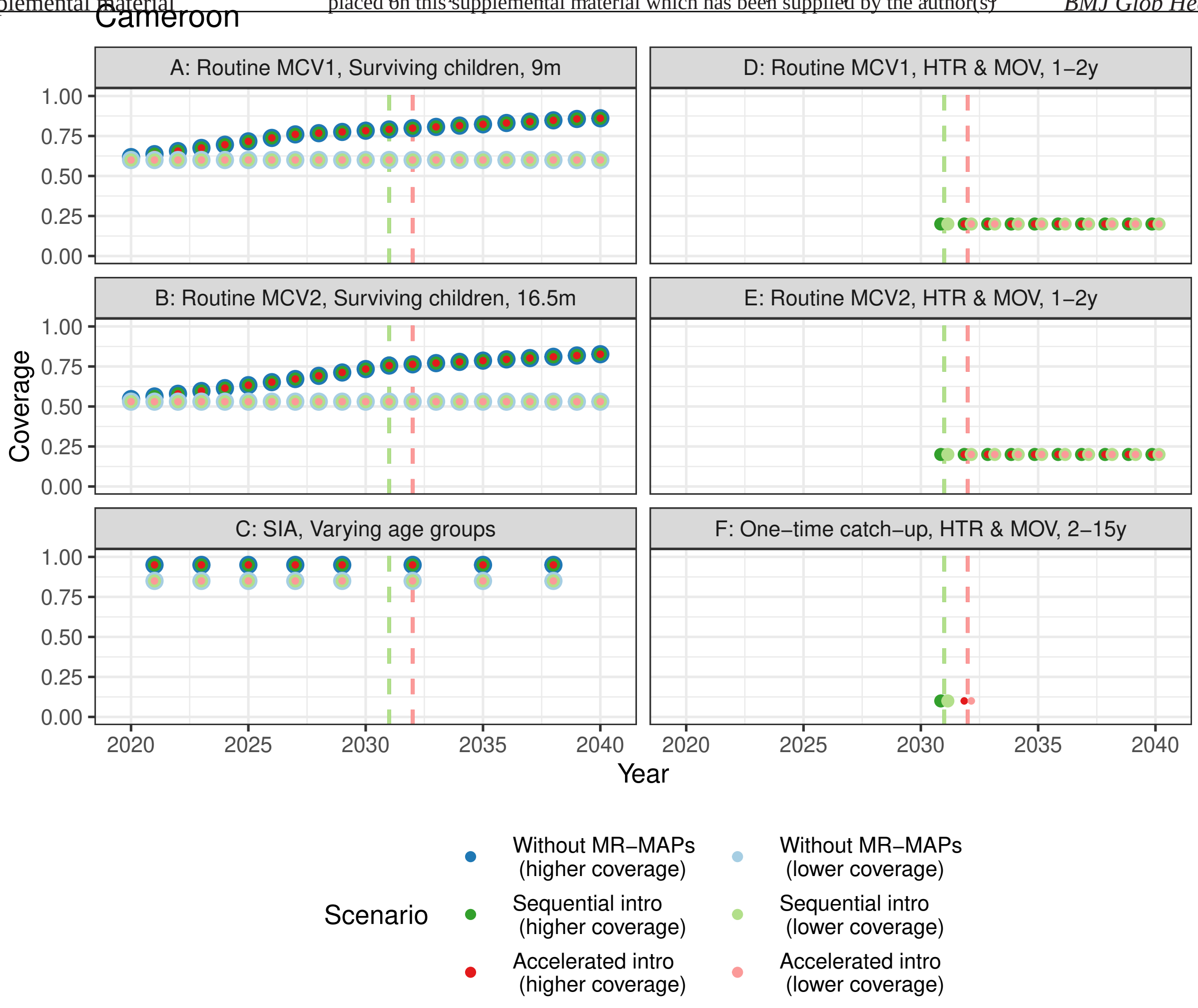
Cameroon
A: Routine MCV1, Surviving children, 9m
D: Routine MCV1, HTR & MOV, 1–2y
B: Routine MCV2, Surviving children, 16.5m
E: Routine MCV2, HTR & MOV, 1–2y
C: SIA, Varying age groups
F: One–time catch–up, HTR & MOV, 2–15y
Coverage
Year
1.00
0.75
0.50
0.25
0.00
2020
2025
2030
2035
2040
Scenario
Without MR–MAPs (higher coverage)
Without MR–MAPs (lower coverage)
Sequential intro (higher coverage)
Sequential intro (lower coverage)
Accelerated intro (higher coverage)
Accelerated intro (lower coverage)

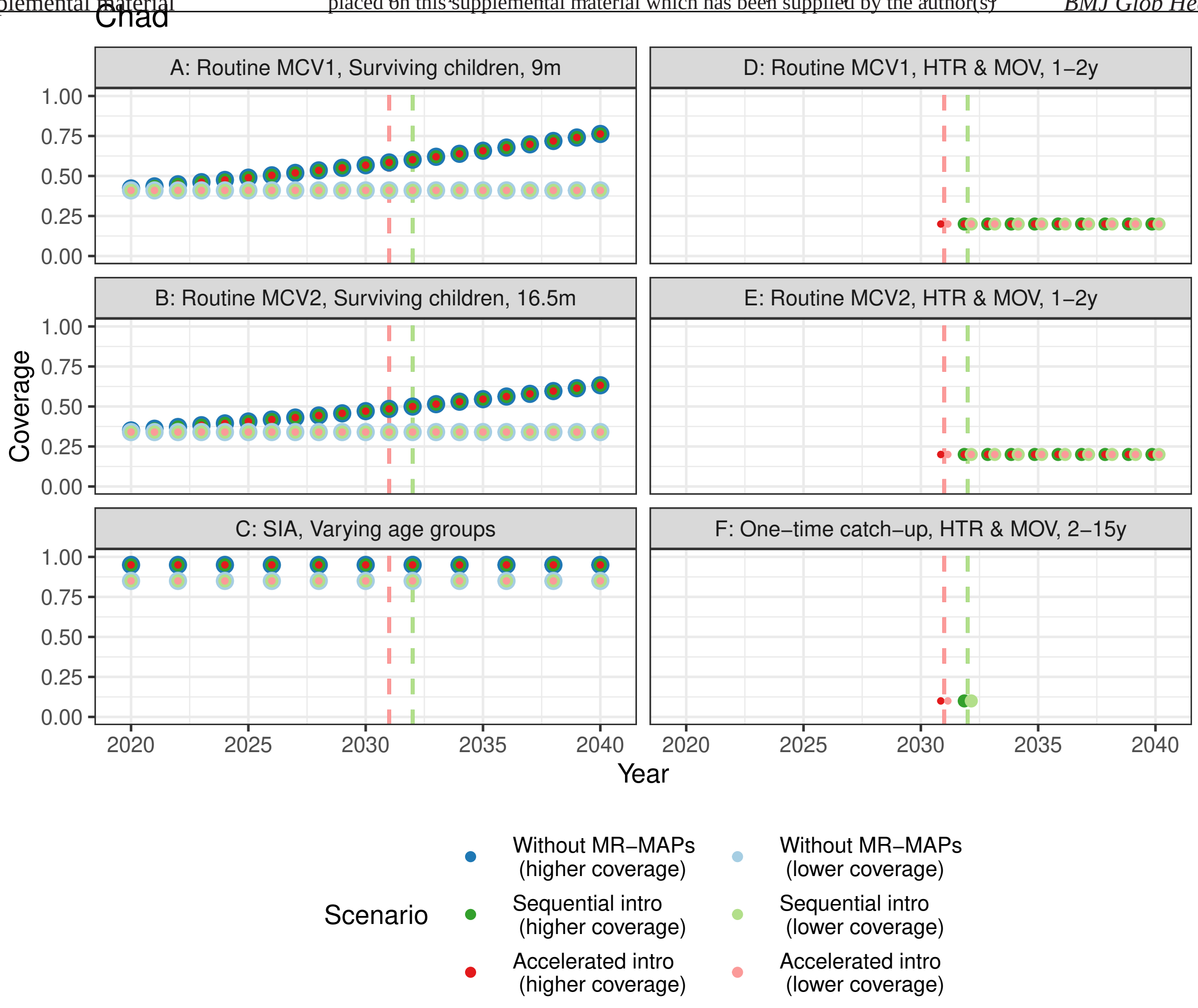
Chad
A: Routine MCV1, Surviving children, 9m
D: Routine MCV1, HTR & MOV, 1−2y
B: Routine MCV2, Surviving children, 16.5m
E: Routine MCV2, HTR & MOV, 1−2y
C: SIA, Varying age groups
F: One−time catch−up, HTR & MOV, 2−15y
Coverage
1.00
0.75
0.50
0.25
0.00
2020
2025
2030
2035
2040
Year
Scenario
Without MR−MAPs (higher coverage)
Without MR−MAPs (lower coverage)
Sequential intro (higher coverage)
Sequential intro (lower coverage)
Accelerated intro (higher coverage)
Accelerated intro (lower coverage)

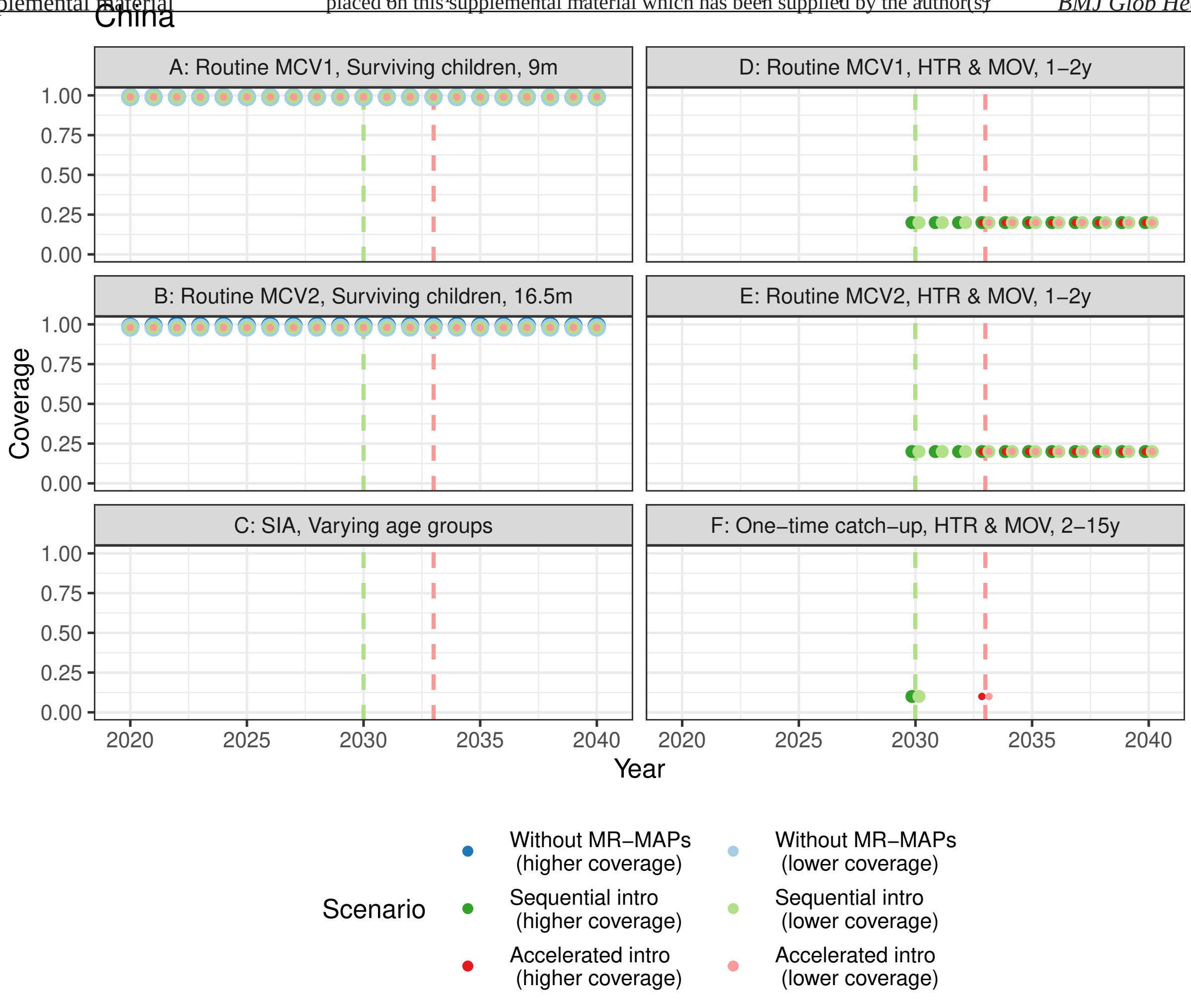
China
A: Routine MCV1, Surviving children, 9m
D: Routine MCV1, HTR & MOV, 1−2y
B: Routine MCV2, Surviving children, 16.5m
E: Routine MCV2, HTR & MOV, 1−2y
C: SIA, Varying age groups
F: One−time catch−up, HTR & MOV, 2−15y
Coverage
1.00
0.75
0.50
0.25
0.00
2020
2025
2030
2035
2040
Year
Scenario
Without MR−MAPs (higher coverage)
Without MR−MAPs (lower coverage)
Sequential intro (higher coverage)
Sequential intro (lower coverage)
Accelerated intro (higher coverage)
Accelerated intro (lower coverage)

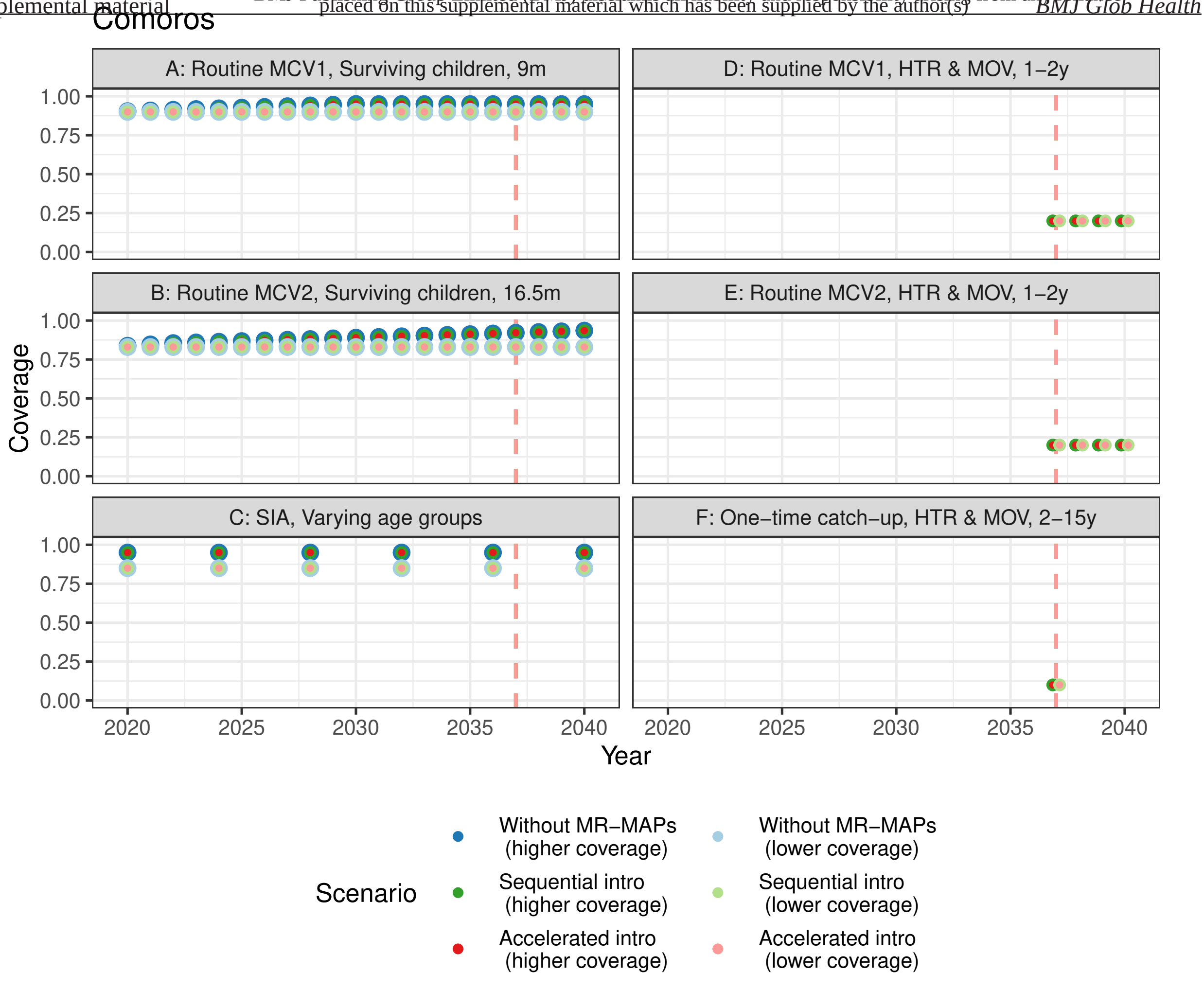
Comoros
A: Routine MCV1, Surviving children, 9m
D: Routine MCV1, HTR & MOV, 1−2y
B: Routine MCV2, Surviving children, 16.5m
E: Routine MCV2, HTR & MOV, 1−2y
C: SIA, Varying age groups
F: One−time catch−up, HTR & MOV, 2−15y
Coverage
1.00
0.75
0.50
0.25
0.00
2020
2025
2030
2035
2040
Year
Scenario
Without MR−MAPs (higher coverage)
Sequential intro (higher coverage)
Accelerated intro (higher coverage)
Without MR−MAPs (lower coverage)
Sequential intro (lower coverage)
Accelerated intro (lower coverage)

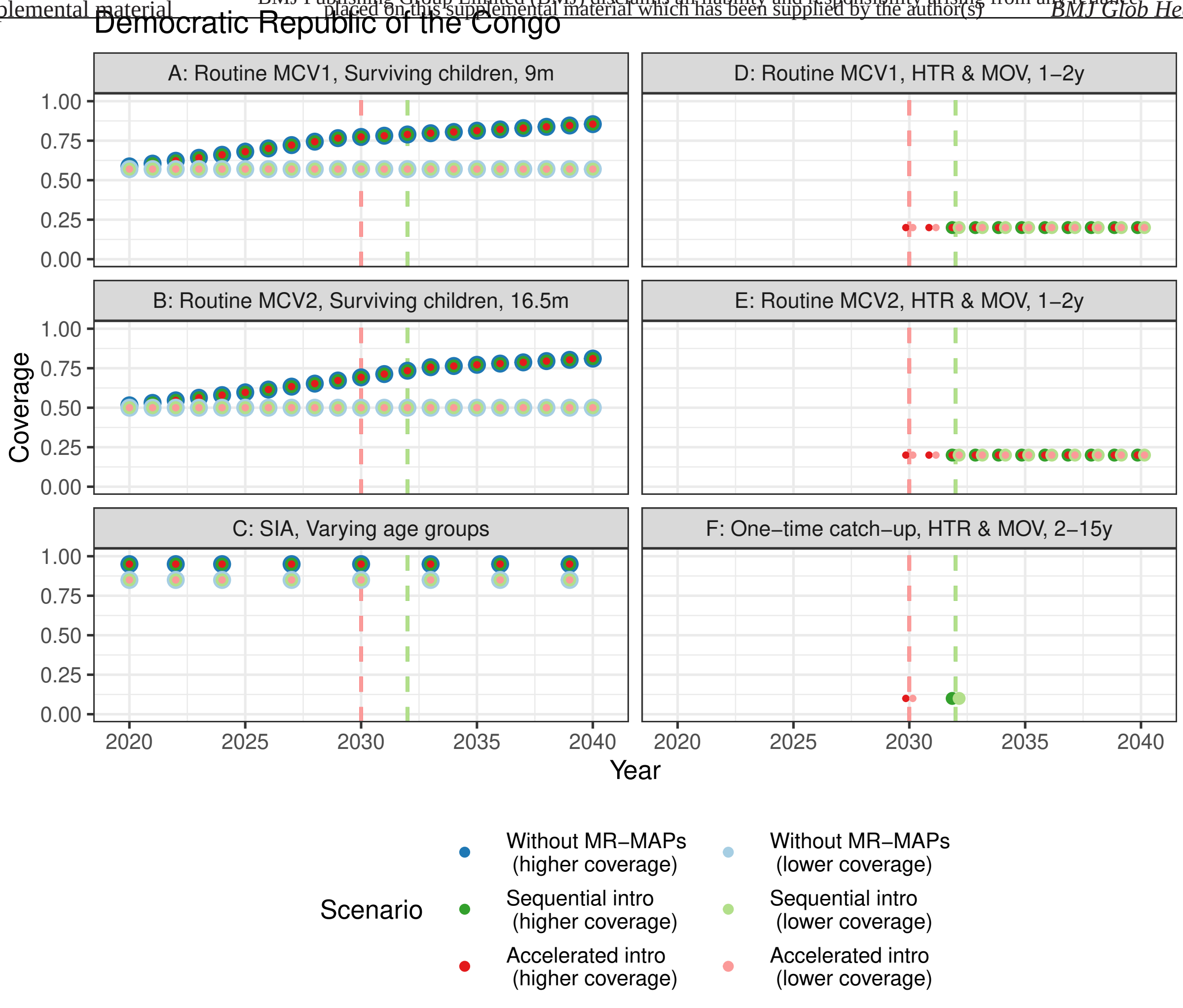
Democratic Republic of the Congo
A: Routine MCV1, Surviving children, 9m
D: Routine MCV1, HTR & MOV, 1−2y
B: Routine MCV2, Surviving children, 16.5m
E: Routine MCV2, HTR & MOV, 1−2y
C: SIA, Varying age groups
F: One−time catch−up, HTR & MOV, 2−15y
Coverage
1.00
0.75
0.50
0.25
0.00
Year
2020
2025
2030
2035
2040
Scenario
Without MR−MAPs (higher coverage)
Sequential intro (higher coverage)
Accelerated intro (higher coverage)
Without MR−MAPs (lower coverage)
Sequential intro (lower coverage)
Accelerated intro (lower coverage)

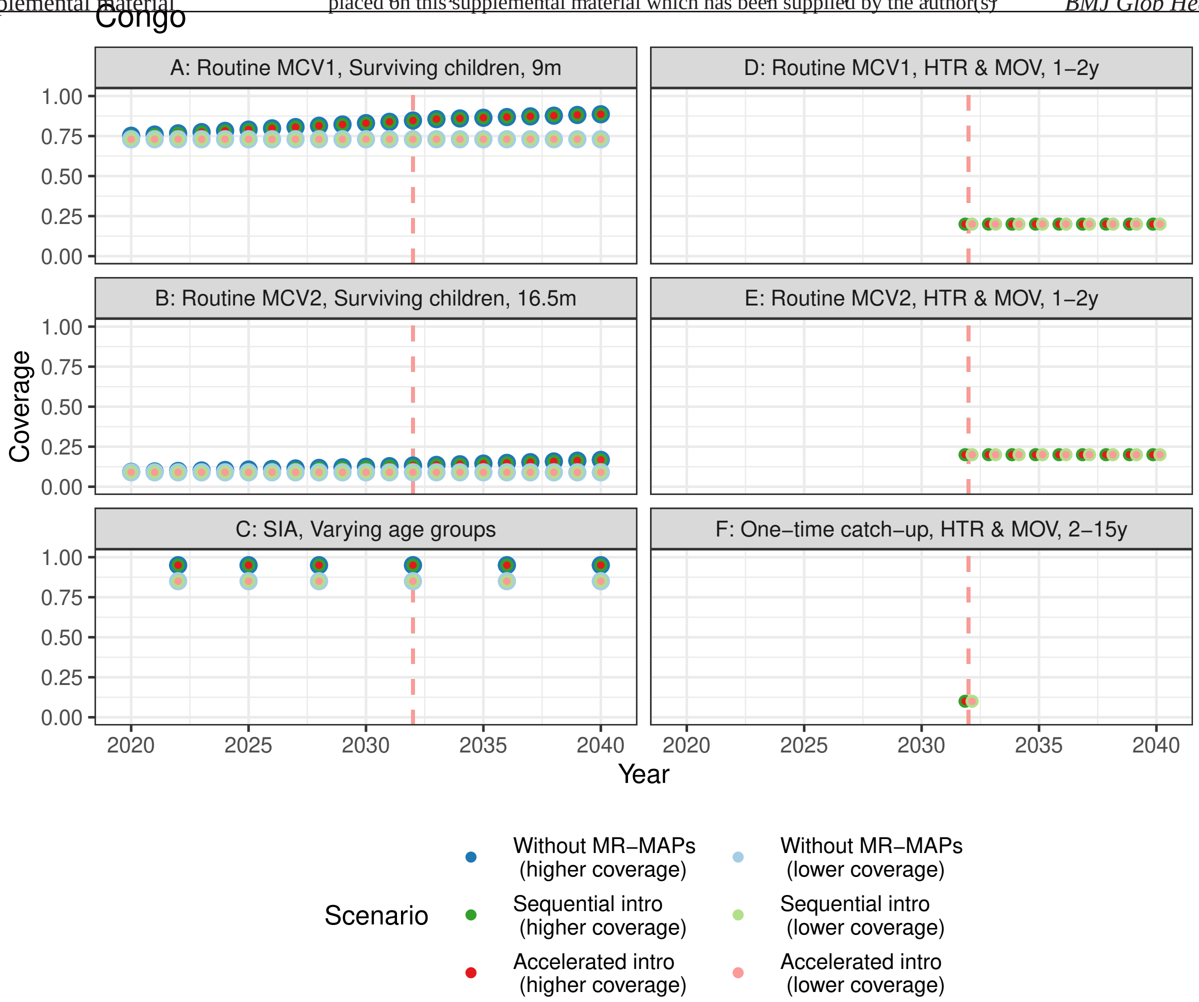
Congo
A: Routine MCV1, Surviving children, 9m
D: Routine MCV1, HTR & MOV, 1–2y
B: Routine MCV2, Surviving children, 16.5m
E: Routine MCV2, HTR & MOV, 1–2y
C: SIA, Varying age groups
F: One–time catch–up, HTR & MOV, 2–15y
Coverage
1.00
0.75
0.50
0.25
0.00
2020
2025
2030
2035
2040
Year
Scenario
Without MR–MAPs (higher coverage)
Sequential intro (higher coverage)
Accelerated intro (higher coverage)
Without MR–MAPs (lower coverage)
Sequential intro (lower coverage)
Accelerated intro (lower coverage)

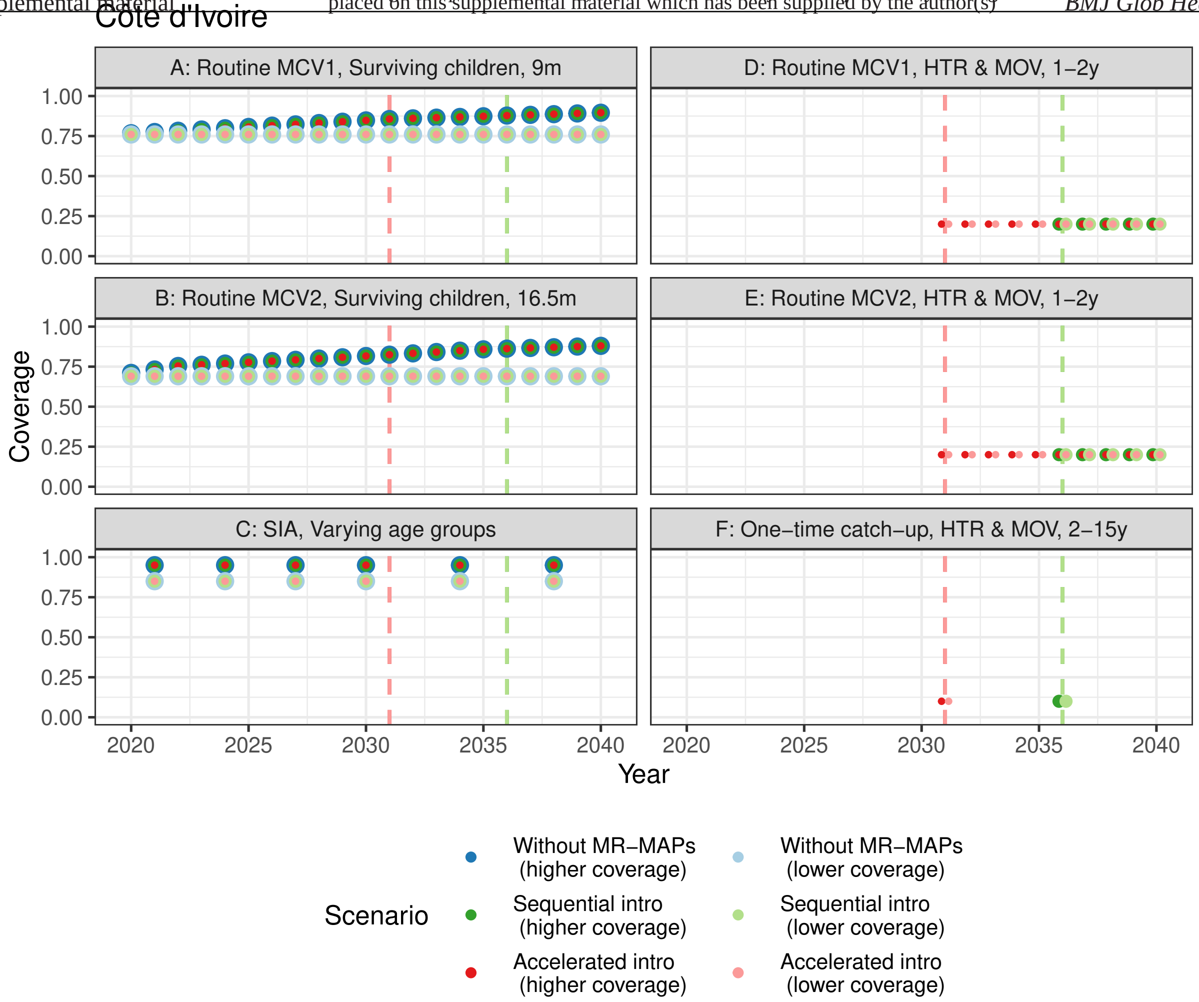
Côte d'Ivoire
A: Routine MCV1, Surviving children, 9m
D: Routine MCV1, HTR & MOV, 1−2y
B: Routine MCV2, Surviving children, 16.5m
E: Routine MCV2, HTR & MOV, 1−2y
C: SIA, Varying age groups
F: One−time catch−up, HTR & MOV, 2−15y
Coverage
1.00
0.75
0.50
0.25
0.00
2020
2025
2030
2035
2040
Year
Scenario
Without MR−MAPs (higher coverage)
Sequential intro (higher coverage)
Accelerated intro (higher coverage)
Without MR−MAPs (lower coverage)
Sequential intro (lower coverage)
Accelerated intro (lower coverage)

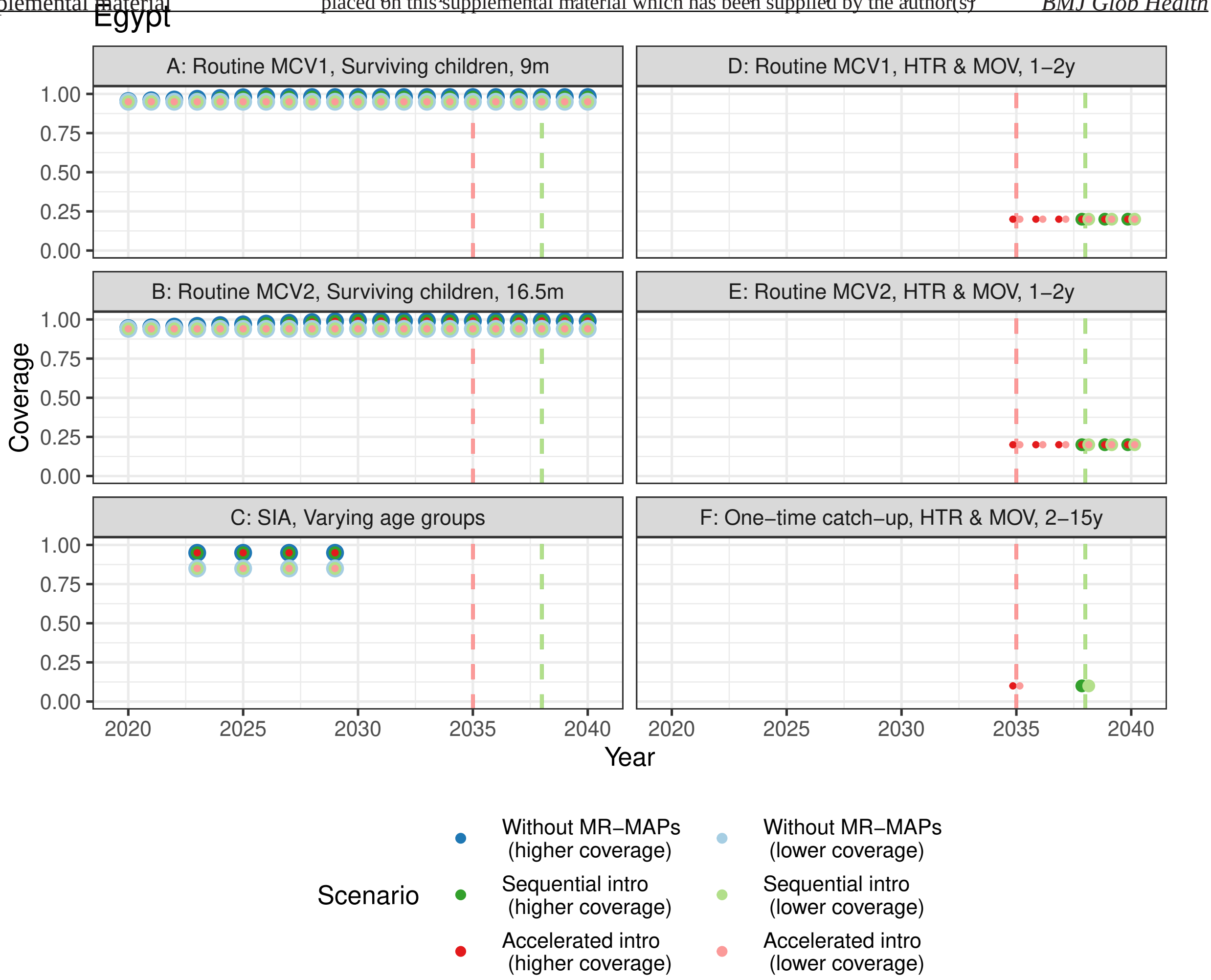
Egypt
A: Routine MCV1, Surviving children, 9m
D: Routine MCV1, HTR & MOV, 1−2y
B: Routine MCV2, Surviving children, 16.5m
E: Routine MCV2, HTR & MOV, 1−2y
C: SIA, Varying age groups
F: One−time catch−up, HTR & MOV, 2−15y
Coverage
1.00
0.75
0.50
0.25
0.00
2020
2025
2030
2035
2040
Year
Scenario
Without MR−MAPs (higher coverage)
Without MR−MAPs (lower coverage)
Sequential intro (higher coverage)
Sequential intro (lower coverage)
Accelerated intro (higher coverage)
Accelerated intro (lower coverage)

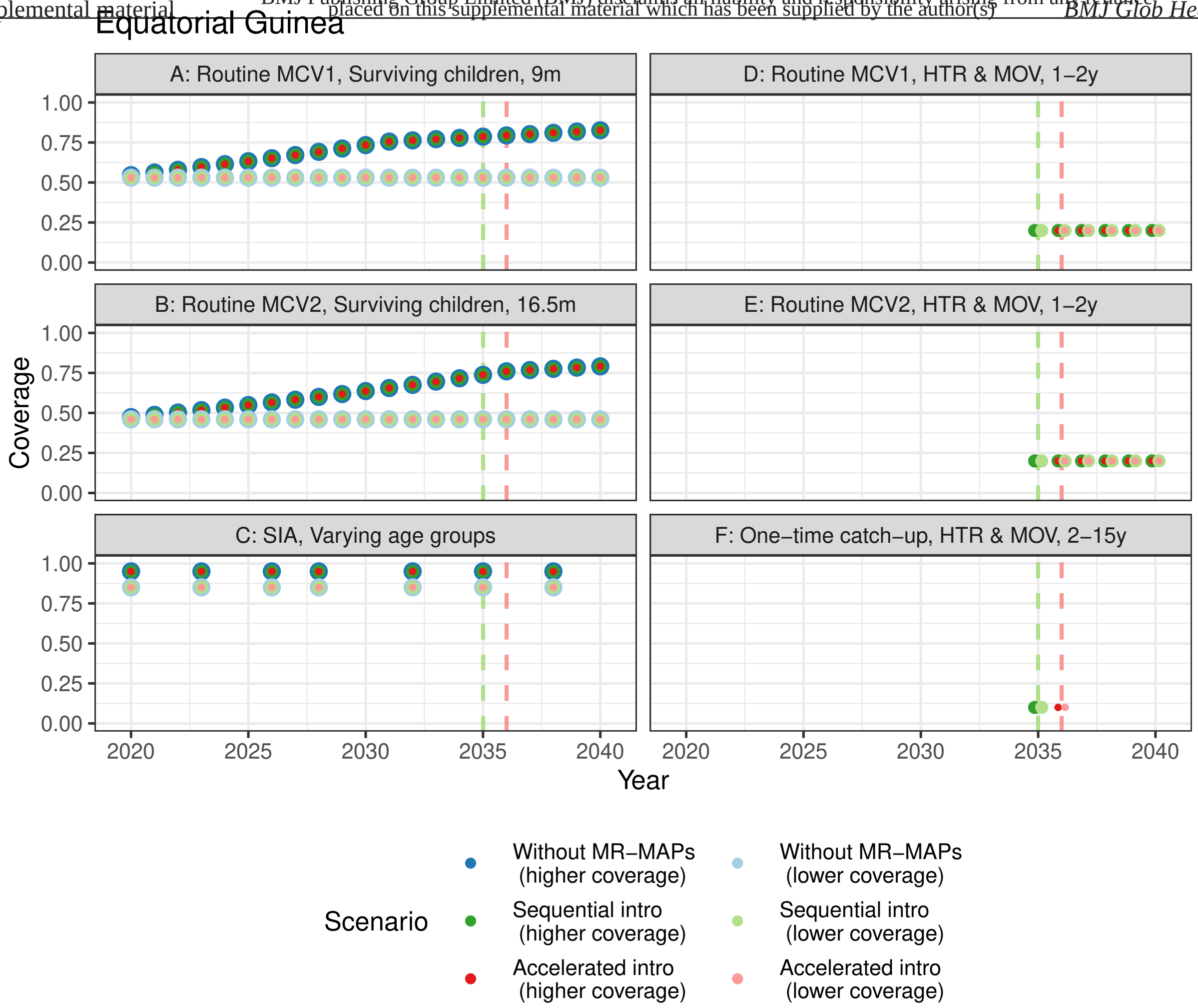
Equatorial Guinea
A: Routine MCV1, Surviving children, 9m
D: Routine MCV1, HTR & MOV, 1−2y
B: Routine MCV2, Surviving children, 16.5m
E: Routine MCV2, HTR & MOV, 1−2y
C: SIA, Varying age groups
F: One−time catch−up, HTR & MOV, 2−15y
Coverage
1.00
0.75
0.50
0.25
0.00
2020
2025
2030
2035
2040
Year
Scenario
Without MR−MAPs (higher coverage)
Without MR−MAPs (lower coverage)
Sequential intro (higher coverage)
Sequential intro (lower coverage)
Accelerated intro (higher coverage)
Accelerated intro (lower coverage)

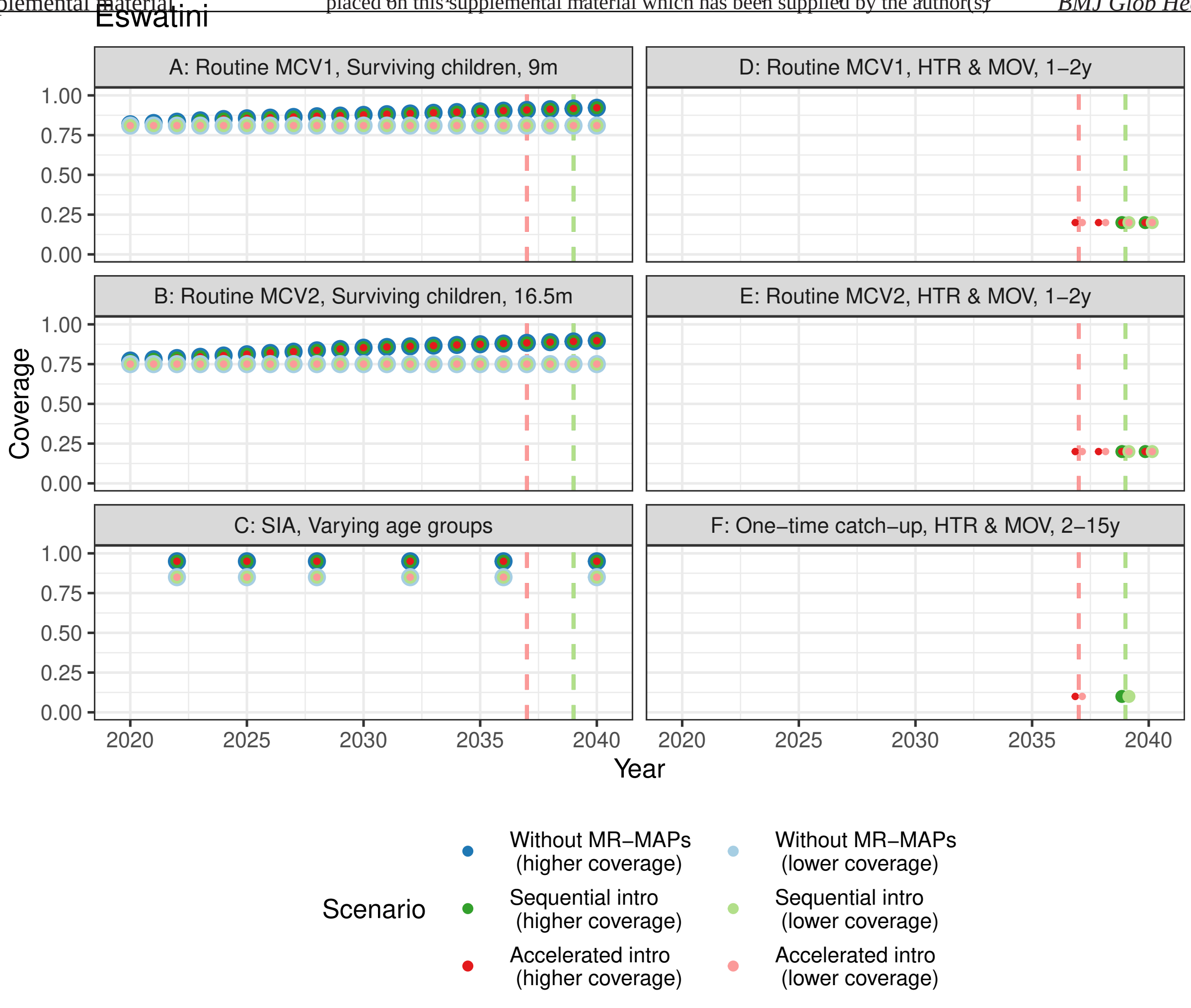
Eswatini
A: Routine MCV1, Surviving children, 9m
D: Routine MCV1, HTR & MOV, 1−2y
B: Routine MCV2, Surviving children, 16.5m
E: Routine MCV2, HTR & MOV, 1−2y
C: SIA, Varying age groups
F: One−time catch−up, HTR & MOV, 2−15y
Coverage
1.00
0.75
0.50
0.25
0.00
2020
2025
2030
2035
2040
Year
Scenario
Without MR−MAPs (higher coverage)
Without MR−MAPs (lower coverage)
Sequential intro (higher coverage)
Sequential intro (lower coverage)
Accelerated intro (higher coverage)
Accelerated intro (lower coverage)

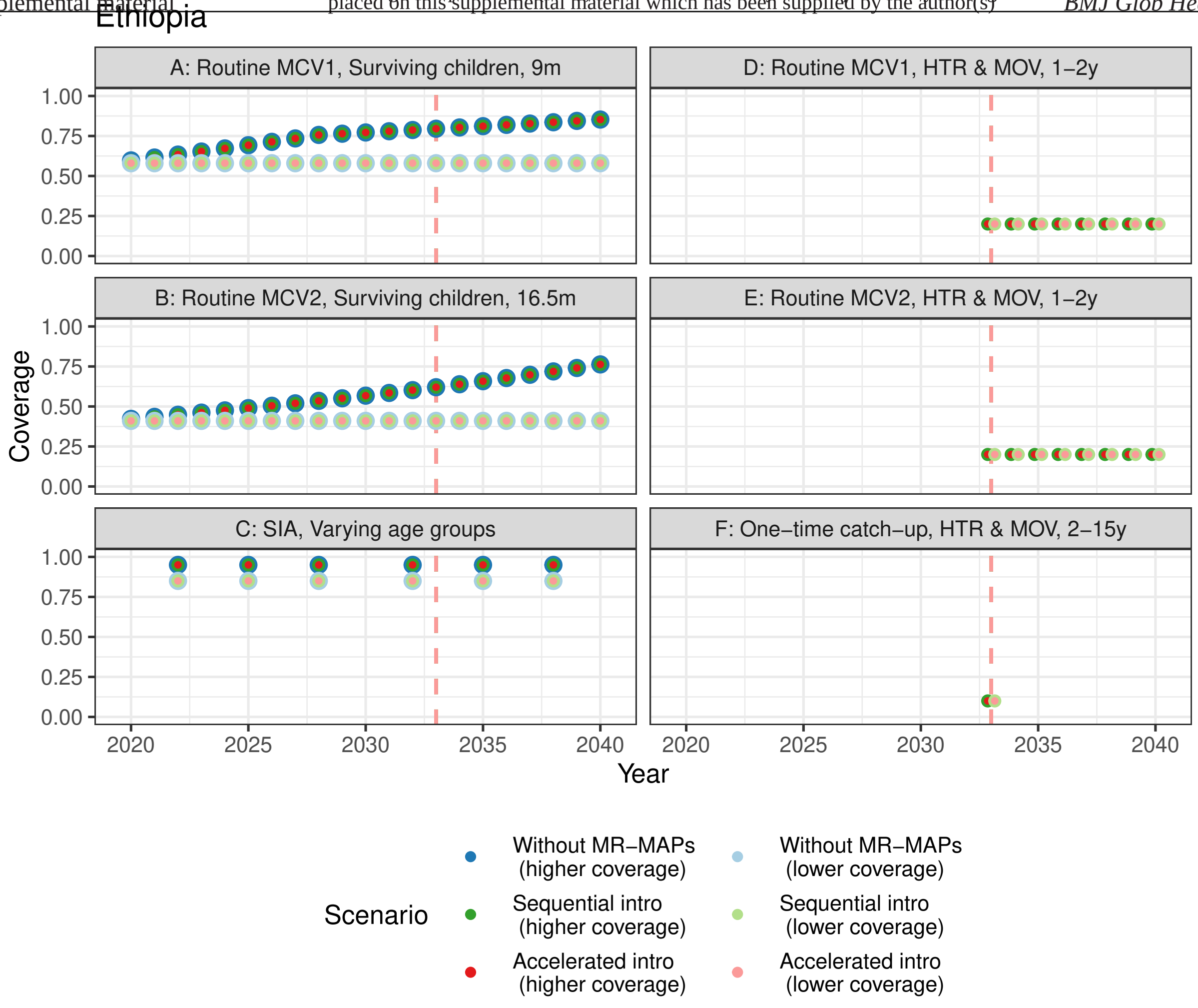
Ethiopia
A: Routine MCV1, Surviving children, 9m
D: Routine MCV1, HTR & MOV, 1−2y
B: Routine MCV2, Surviving children, 16.5m
E: Routine MCV2, HTR & MOV, 1−2y
C: SIA, Varying age groups
F: One−time catch−up, HTR & MOV, 2−15y
Coverage
1.00
0.75
0.50
0.25
0.00
2020
2025
2030
2035
2040
Year
Scenario
Without MR−MAPs (higher coverage)
Sequential intro (higher coverage)
Accelerated intro (higher coverage)
Without MR−MAPs (lower coverage)
Sequential intro (lower coverage)
Accelerated intro (lower coverage)

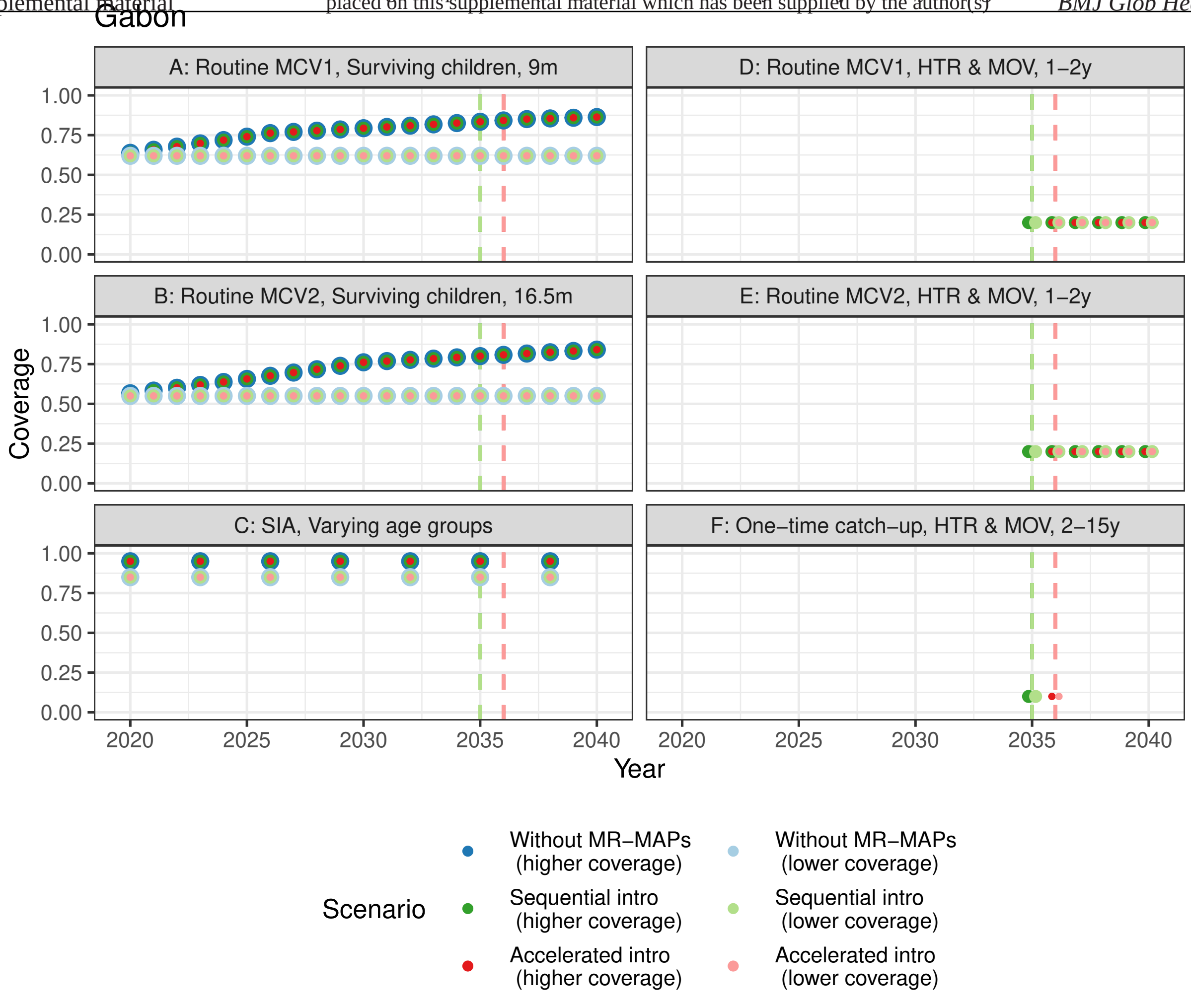
Gabon
A: Routine MCV1, Surviving children, 9m
D: Routine MCV1, HTR & MOV, 1−2y
B: Routine MCV2, Surviving children, 16.5m
E: Routine MCV2, HTR & MOV, 1−2y
C: SIA, Varying age groups
F: One−time catch−up, HTR & MOV, 2−15y
Coverage
1.00
0.75
0.50
0.25
0.00
2020
2025
2030
2035
2040
Year
Scenario
Without MR−MAPs (higher coverage)
Without MR−MAPs (lower coverage)
Sequential intro (higher coverage)
Sequential intro (lower coverage)
Accelerated intro (higher coverage)
Accelerated intro (lower coverage)

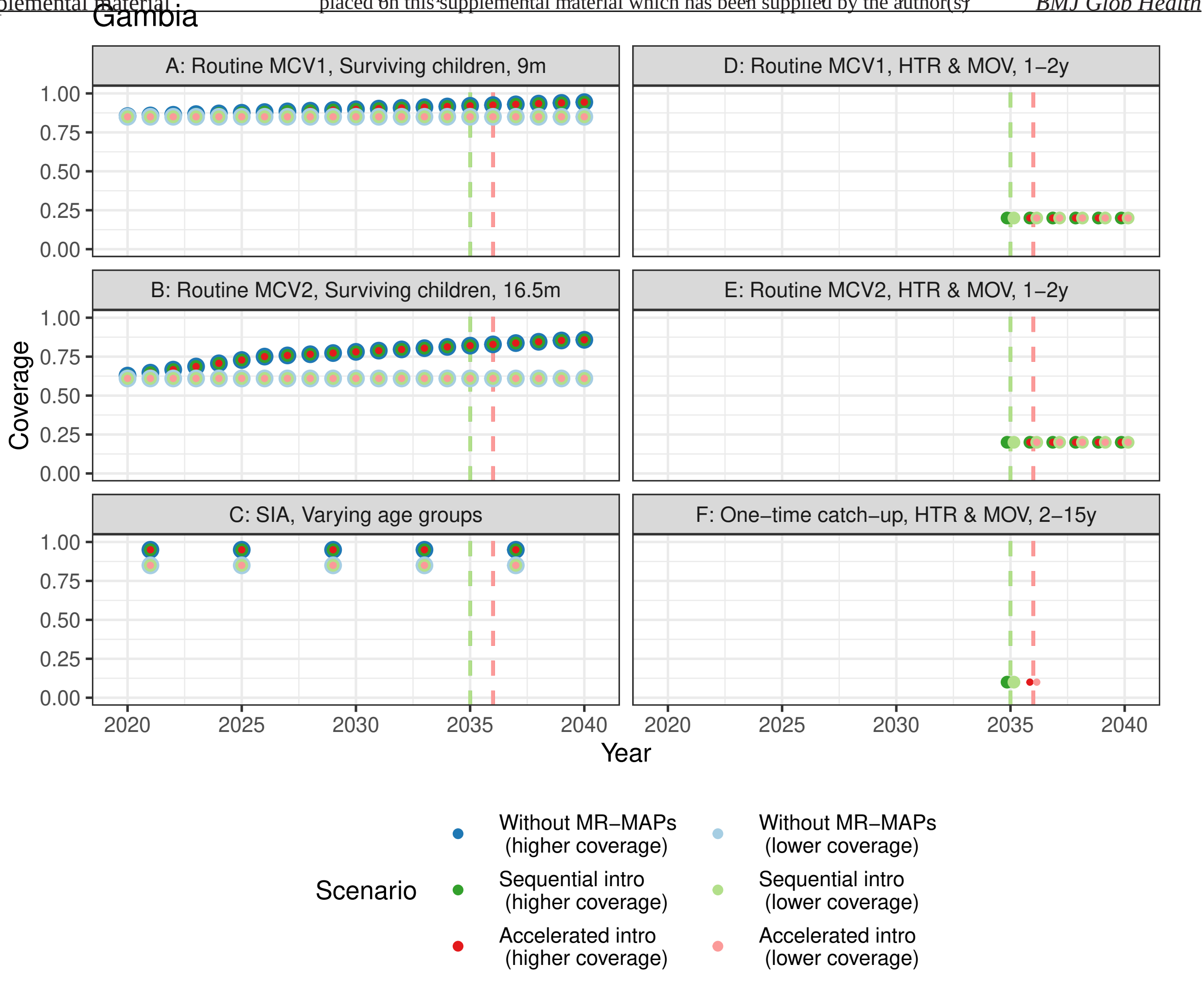
Gambia
A: Routine MCV1, Surviving children, 9m
D: Routine MCV1, HTR & MOV, 1−2y
B: Routine MCV2, Surviving children, 16.5m
E: Routine MCV2, HTR & MOV, 1−2y
C: SIA, Varying age groups
F: One−time catch−up, HTR & MOV, 2−15y
Coverage
1.00
0.75
0.50
0.25
0.00
2020
2025
2030
2035
2040
Year
Scenario
Without MR−MAPs (higher coverage)
Without MR−MAPs (lower coverage)
Sequential intro (higher coverage)
Sequential intro (lower coverage)
Accelerated intro (higher coverage)
Accelerated intro (lower coverage)

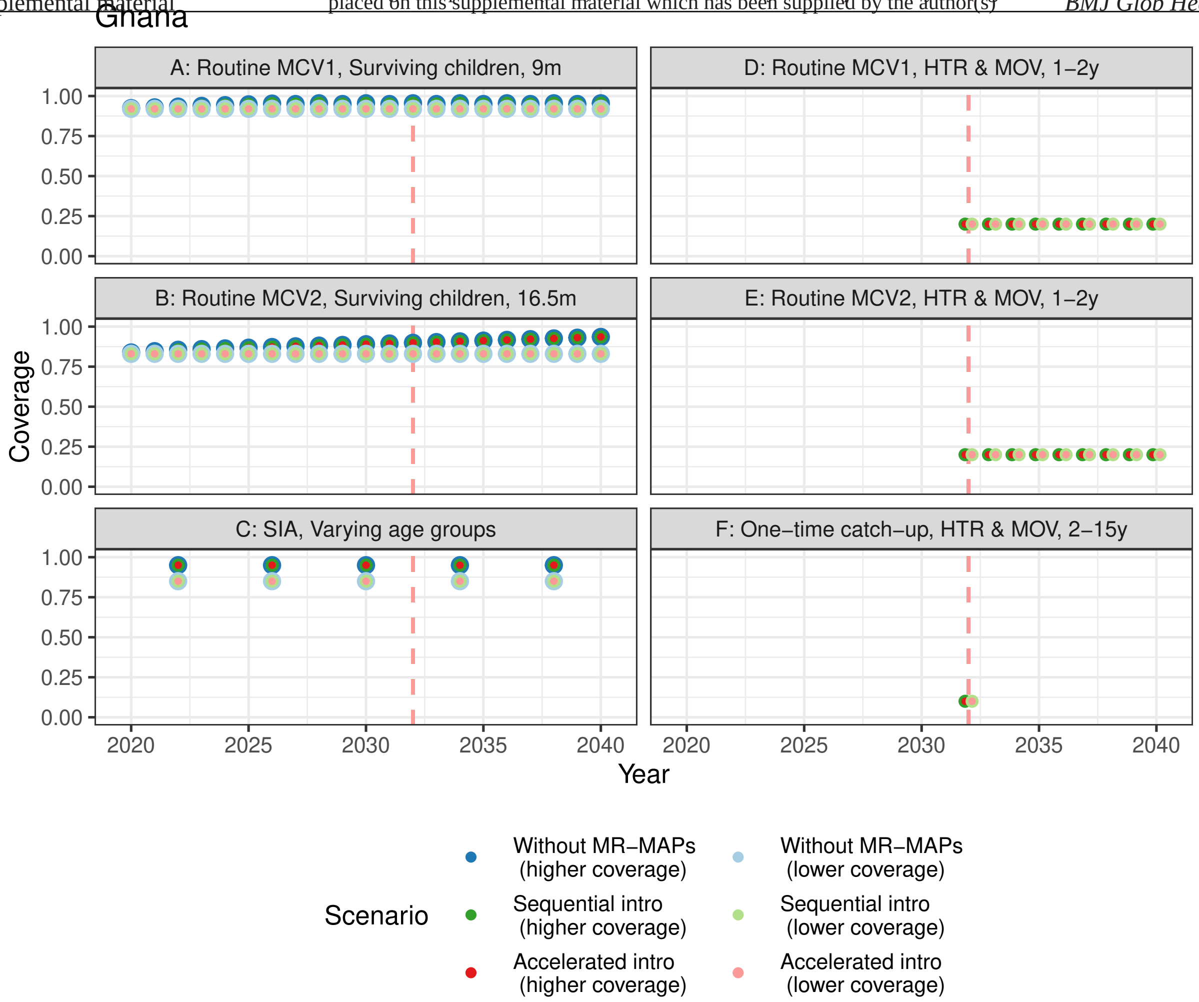
Ghana
A: Routine MCV1, Surviving children, 9m
D: Routine MCV1, HTR & MOV, 1−2y
B: Routine MCV2, Surviving children, 16.5m
E: Routine MCV2, HTR & MOV, 1−2y
C: SIA, Varying age groups
F: One−time catch−up, HTR & MOV, 2−15y
Coverage
1.00
0.75
0.50
0.25
0.00
2020
2025
2030
2035
2040
Year
Scenario
Without MR−MAPs (higher coverage)
Sequential intro (higher coverage)
Accelerated intro (higher coverage)
Without MR−MAPs (lower coverage)
Sequential intro (lower coverage)
Accelerated intro (lower coverage)

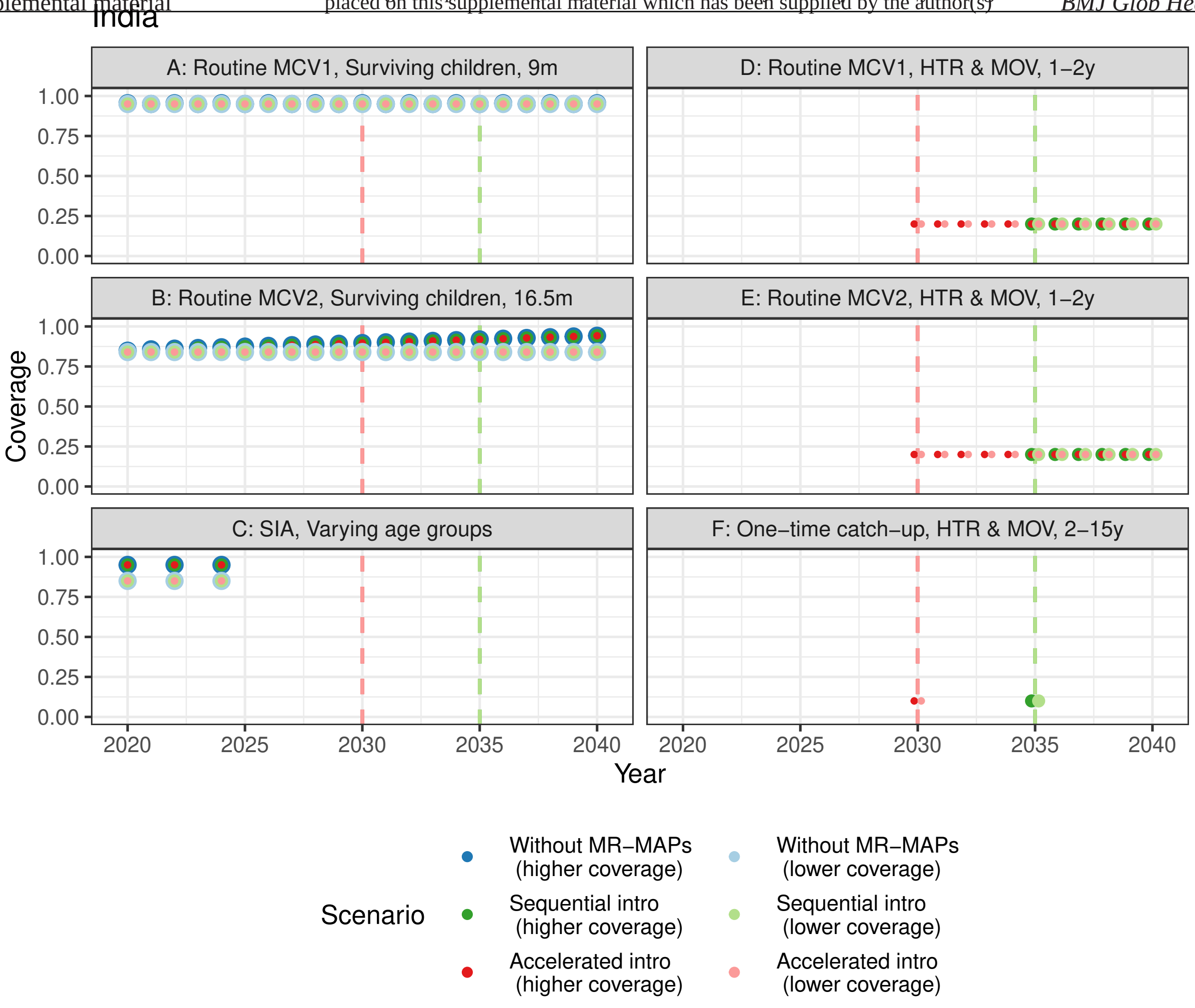
India
A: Routine MCV1, Surviving children, 9m
D: Routine MCV1, HTR & MOV, 1–2y
B: Routine MCV2, Surviving children, 16.5m
E: Routine MCV2, HTR & MOV, 1–2y
C: SIA, Varying age groups
F: One−time catch−up, HTR & MOV, 2–15y
Coverage
1.00
0.75
0.50
0.25
0.00
2020
2025
2030
2035
2040
Year
Scenario
Without MR−MAPs (higher coverage)
Without MR−MAPs (lower coverage)
Sequential intro (higher coverage)
Sequential intro (lower coverage)
Accelerated intro (higher coverage)
Accelerated intro (lower coverage)

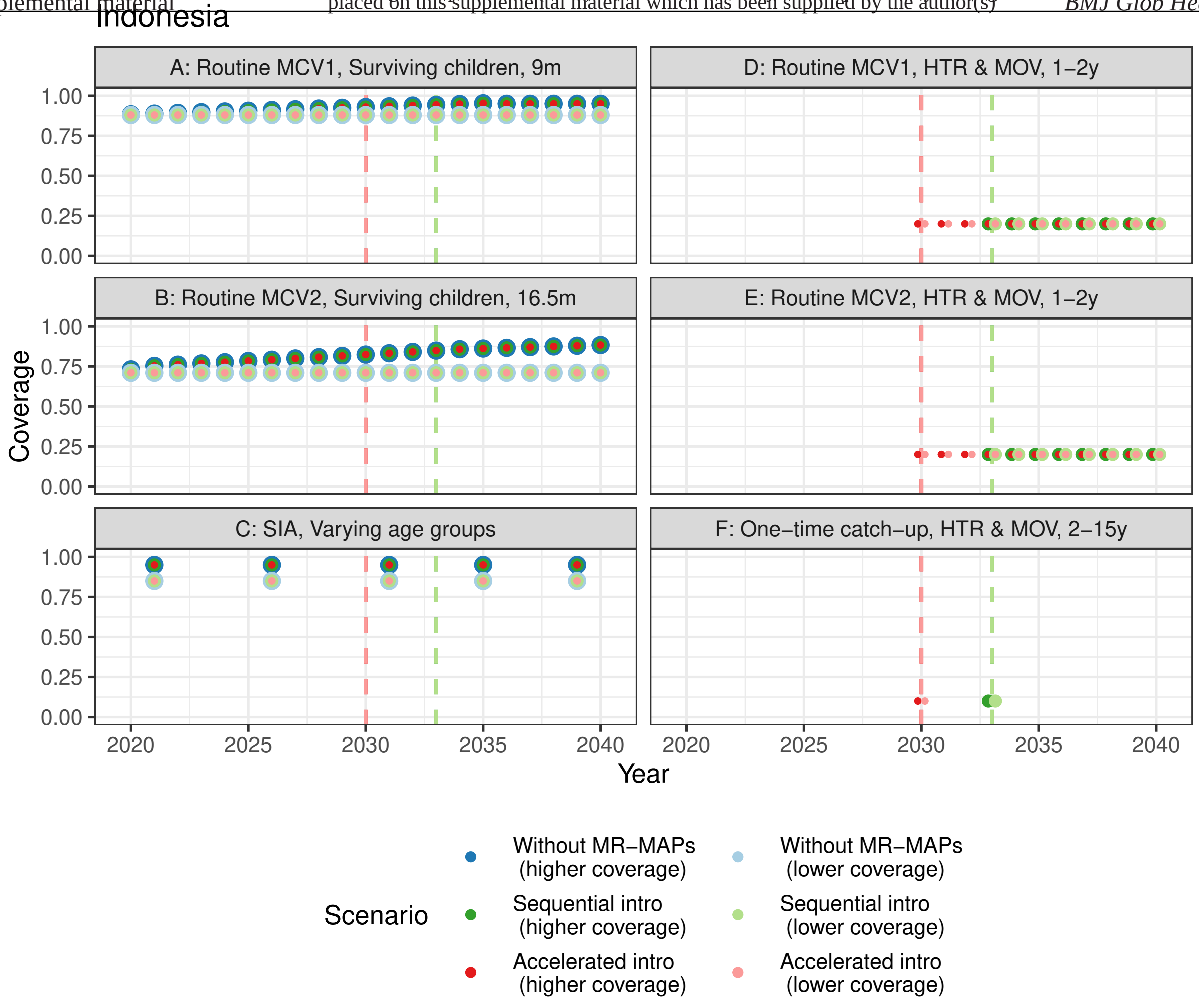
Indonesia
A: Routine MCV1, Surviving children, 9m
D: Routine MCV1, HTR & MOV, 1−2y
B: Routine MCV2, Surviving children, 16.5m
E: Routine MCV2, HTR & MOV, 1−2y
C: SIA, Varying age groups
F: One−time catch−up, HTR & MOV, 2−15y
Coverage
1.00
0.75
0.50
0.25
0.00
2020
2025
2030
2035
2040
Year
Scenario
Without MR−MAPs (higher coverage)
Sequential intro (higher coverage)
Accelerated intro (higher coverage)
Without MR−MAPs (lower coverage)
Sequential intro (lower coverage)
Accelerated intro (lower coverage)

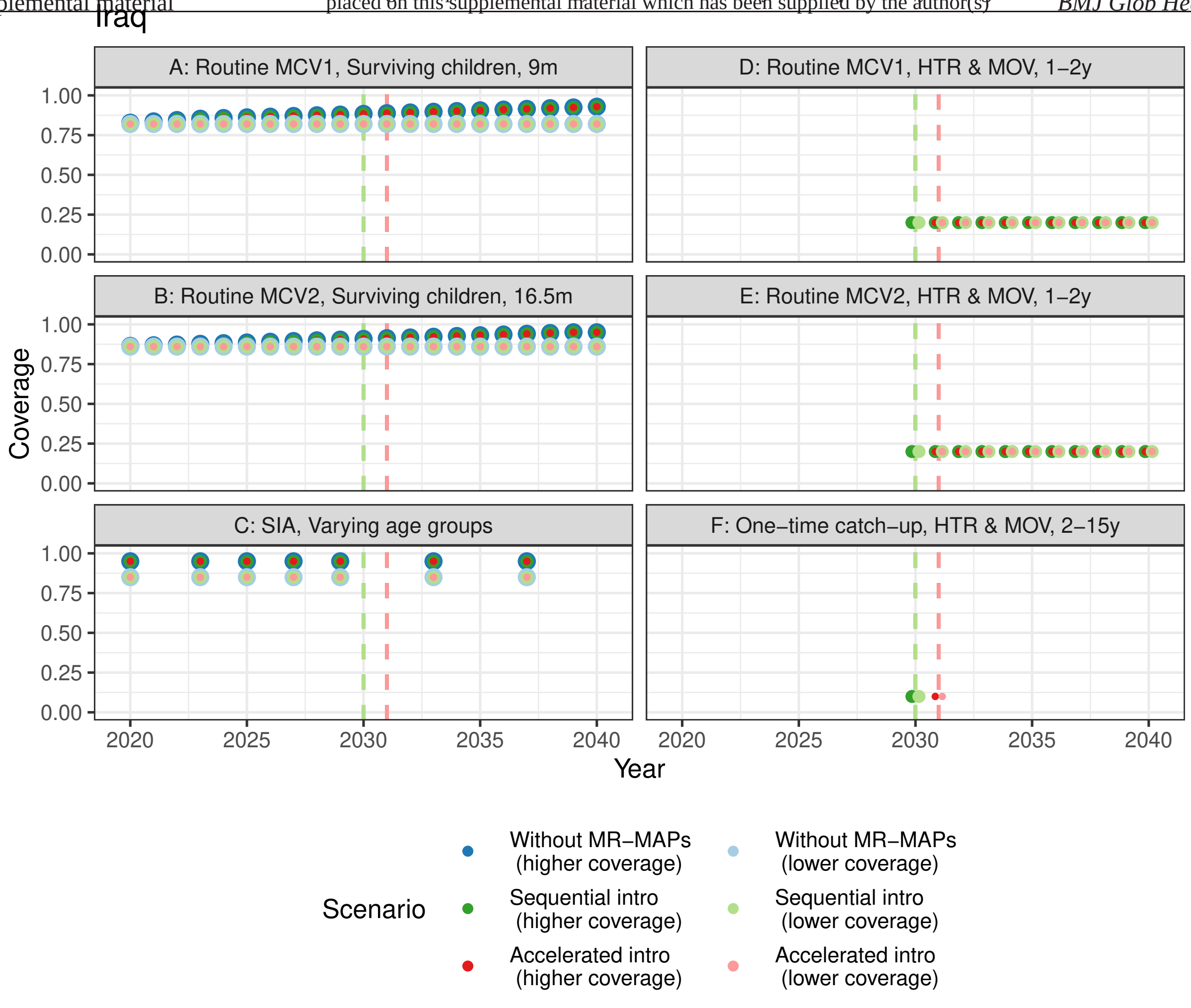
Iraq
A: Routine MCV1, Surviving children, 9m
D: Routine MCV1, HTR & MOV, 1−2y
B: Routine MCV2, Surviving children, 16.5m
E: Routine MCV2, HTR & MOV, 1−2y
C: SIA, Varying age groups
F: One−time catch−up, HTR & MOV, 2−15y
Coverage
1.00
0.75
0.50
0.25
0.00
2020
2025
2030
2035
2040
Year
Scenario
Without MR−MAPs (higher coverage)
Without MR−MAPs (lower coverage)
Sequential intro (higher coverage)
Sequential intro (lower coverage)
Accelerated intro (higher coverage)
Accelerated intro (lower coverage)

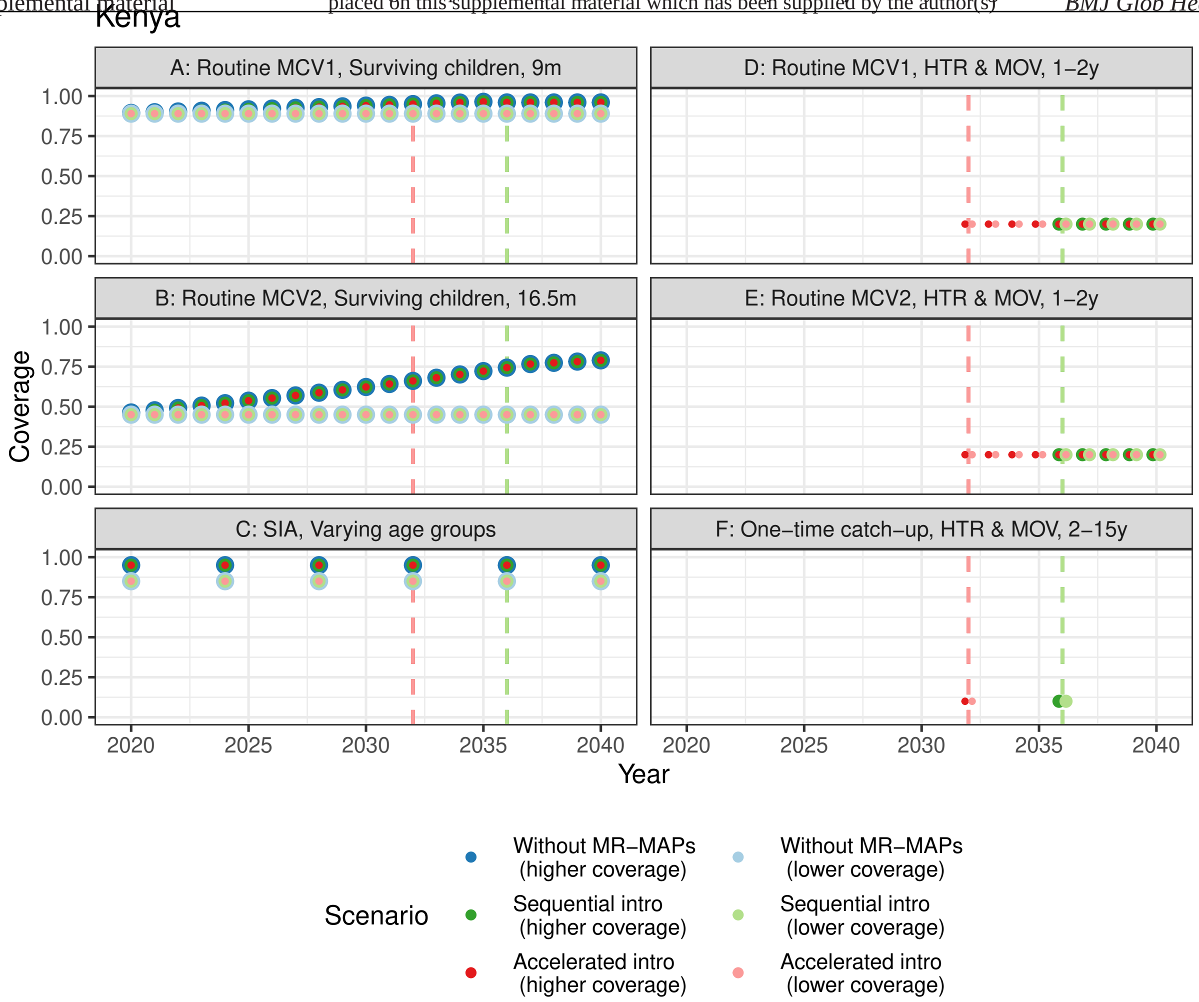
Kenya
A: Routine MCV1, Surviving children, 9m
D: Routine MCV1, HTR & MOV, 1−2y
B: Routine MCV2, Surviving children, 16.5m
E: Routine MCV2, HTR & MOV, 1−2y
C: SIA, Varying age groups
F: One−time catch−up, HTR & MOV, 2−15y
Coverage
1.00
0.75
0.50
0.25
0.00
2020
2025
2030
2035
2040
Year
Scenario
Without MR−MAPs (higher coverage)
Without MR−MAPs (lower coverage)
Sequential intro (higher coverage)
Sequential intro (lower coverage)
Accelerated intro (higher coverage)
Accelerated intro (lower coverage)

Kiribati

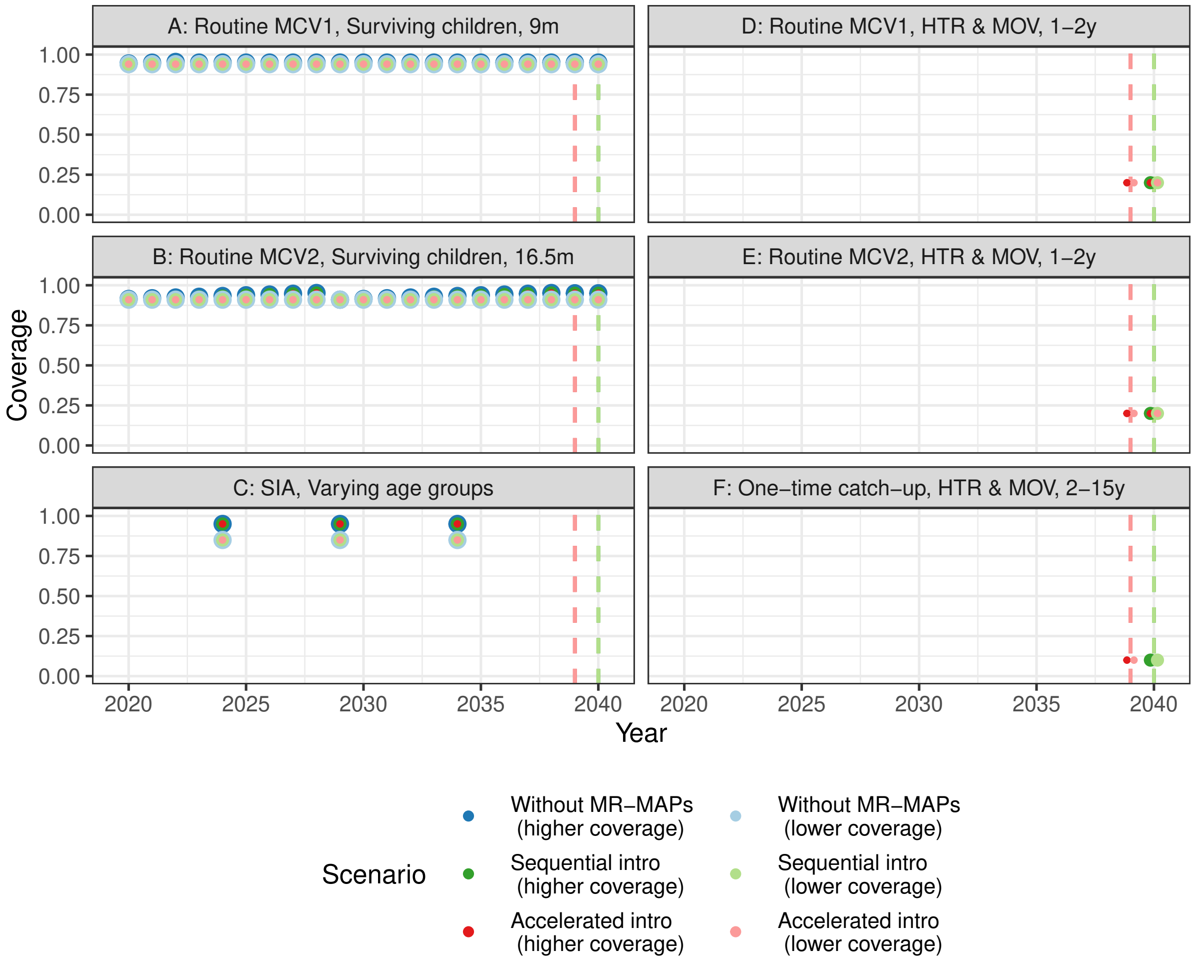
A: Routine MCV1, Surviving children, 9m
D: Routine MCV1, HTR & MOV, 1−2y
B: Routine MCV2, Surviving children, 16.5m
E: Routine MCV2, HTR & MOV, 1−2y
C: SIA, Varying age groups
F: One−time catch−up, HTR & MOV, 2−15y
Coverage
1.00
0.75
0.50
0.25
0.00
2020
2025
2030
2035
2040
Year
Scenario
Without MR−MAPs (higher coverage)
Without MR−MAPs (lower coverage)
Sequential intro (higher coverage)
Sequential intro (lower coverage)
Accelerated intro (higher coverage)
Accelerated intro (lower coverage)

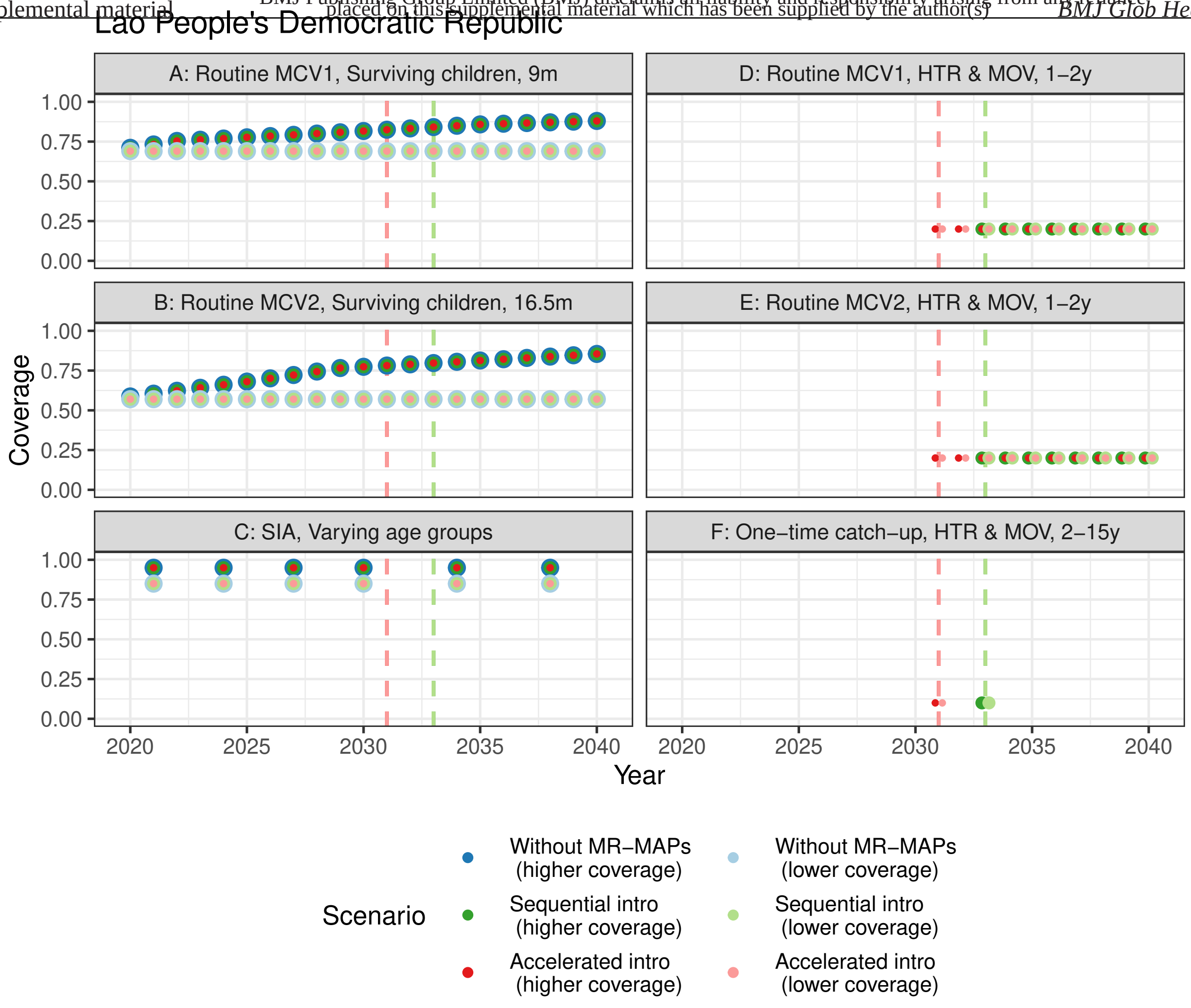
Lao People's Democratic Republic
A: Routine MCV1, Surviving children, 9m
D: Routine MCV1, HTR & MOV, 1−2y
B: Routine MCV2, Surviving children, 16.5m
E: Routine MCV2, HTR & MOV, 1−2y
C: SIA, Varying age groups
F: One−time catch−up, HTR & MOV, 2−15y
Coverage
1.00
0.75
0.50
0.25
0.00
2020
2025
2030
2035
2040
Year
Scenario
Without MR−MAPs (higher coverage)
Without MR−MAPs (lower coverage)
Sequential intro (higher coverage)
Sequential intro (lower coverage)
Accelerated intro (higher coverage)
Accelerated intro (lower coverage)

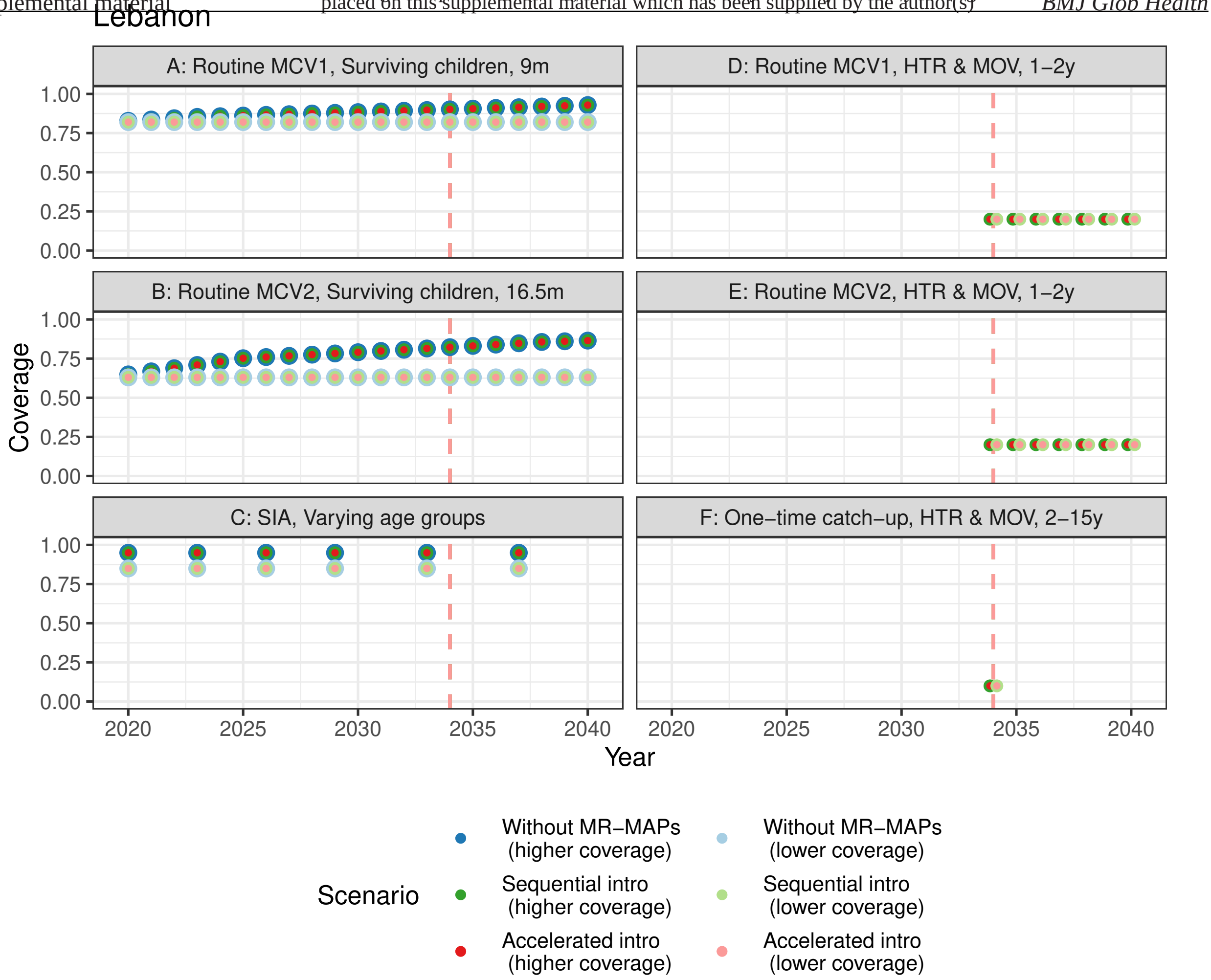
Lebanon
A: Routine MCV1, Surviving children, 9m
D: Routine MCV1, HTR & MOV, 1–2y
B: Routine MCV2, Surviving children, 16.5m
E: Routine MCV2, HTR & MOV, 1–2y
C: SIA, Varying age groups
F: One–time catch–up, HTR & MOV, 2–15y
Coverage
1.00
0.75
0.50
0.25
0.00
Year
2020
2025
2030
2035
2040
Scenario
Without MR–MAPs (higher coverage)
Sequential intro (higher coverage)
Accelerated intro (higher coverage)
Without MR–MAPs (lower coverage)
Sequential intro (lower coverage)
Accelerated intro (lower coverage)

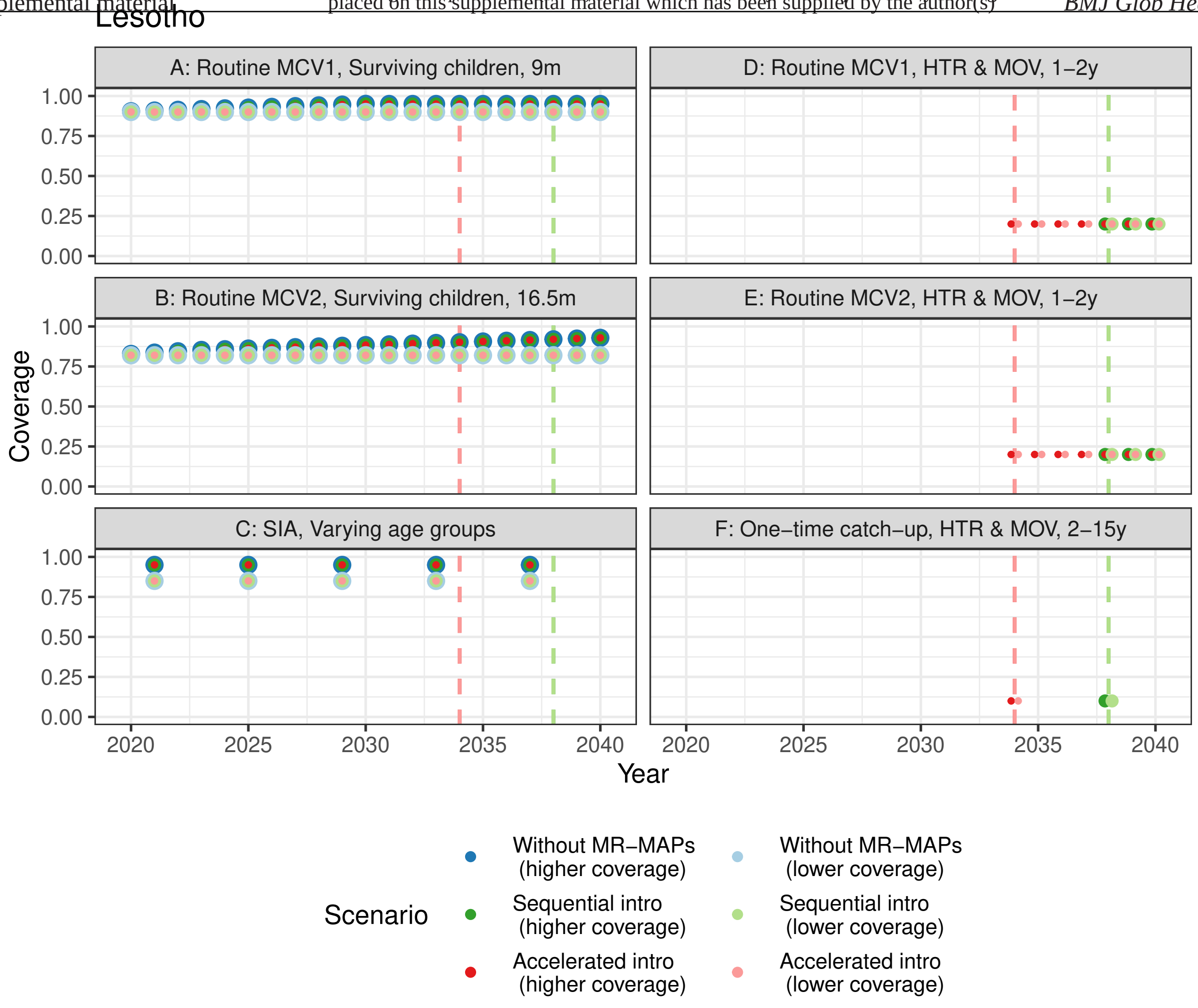
Lesotho
A: Routine MCV1, Surviving children, 9m
B: Routine MCV2, Surviving children, 16.5m
C: SIA, Varying age groups
D: Routine MCV1, HTR & MOV, 1–2y
E: Routine MCV2, HTR & MOV, 1–2y
F: One–time catch–up, HTR & MOV, 2–15y
Coverage
1.00
0.75
0.50
0.25
0.00
2020
2025
2030
2035
2040
Year
Scenario
Without MR–MAPs (higher coverage)
Sequential intro (higher coverage)
Accelerated intro (higher coverage)
Without MR–MAPs (lower coverage)
Sequential intro (lower coverage)
Accelerated intro (lower coverage)

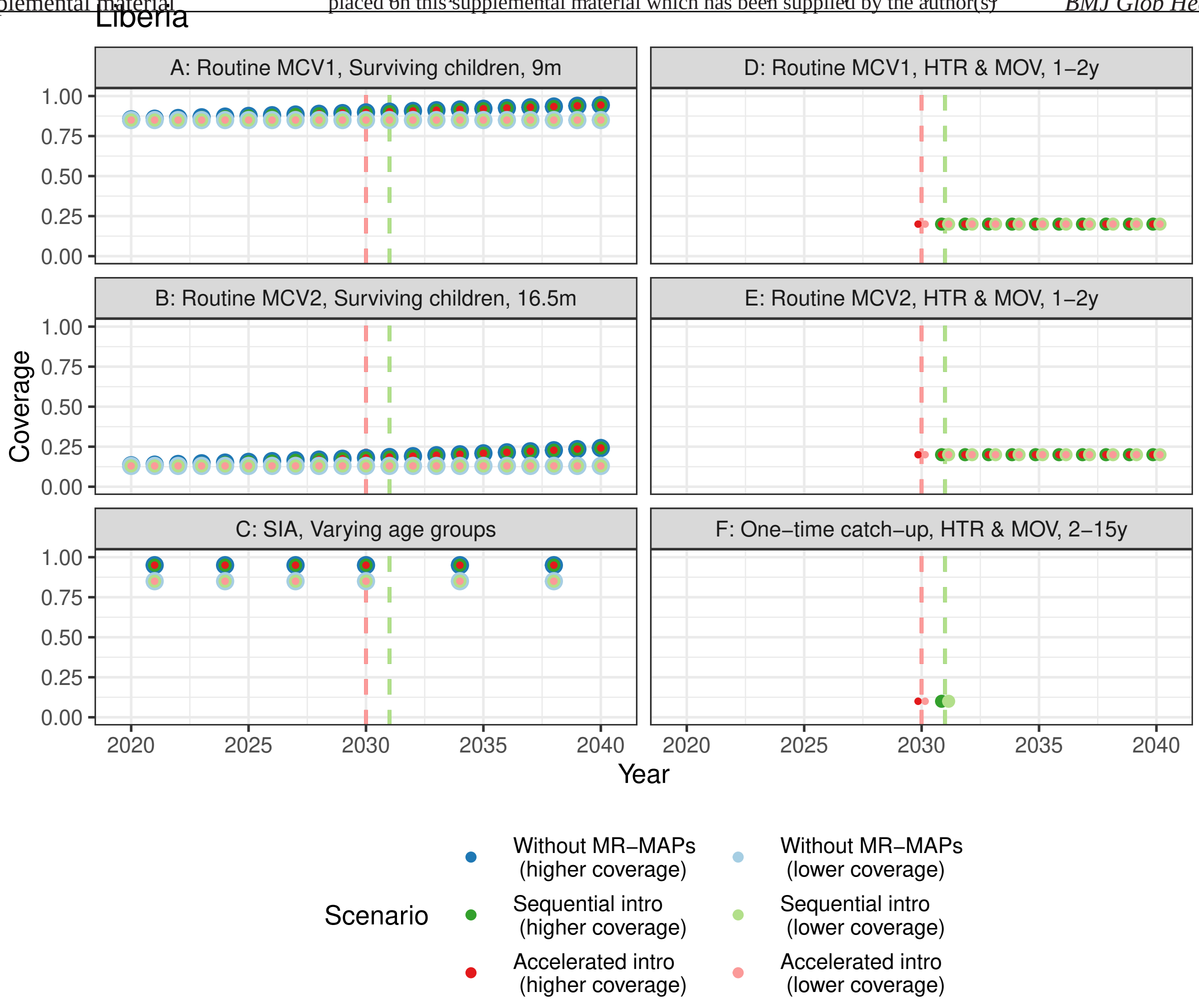
Liberia
A: Routine MCV1, Surviving children, 9m
D: Routine MCV1, HTR & MOV, 1–2y
B: Routine MCV2, Surviving children, 16.5m
E: Routine MCV2, HTR & MOV, 1–2y
C: SIA, Varying age groups
F: One−time catch−up, HTR & MOV, 2–15y
Coverage
1.00
0.75
0.50
0.25
0.00
2020
2025
2030
2035
2040
Year
Scenario
Without MR−MAPs (higher coverage)
Sequential intro (higher coverage)
Accelerated intro (higher coverage)
Without MR−MAPs (lower coverage)
Sequential intro (lower coverage)
Accelerated intro (lower coverage)

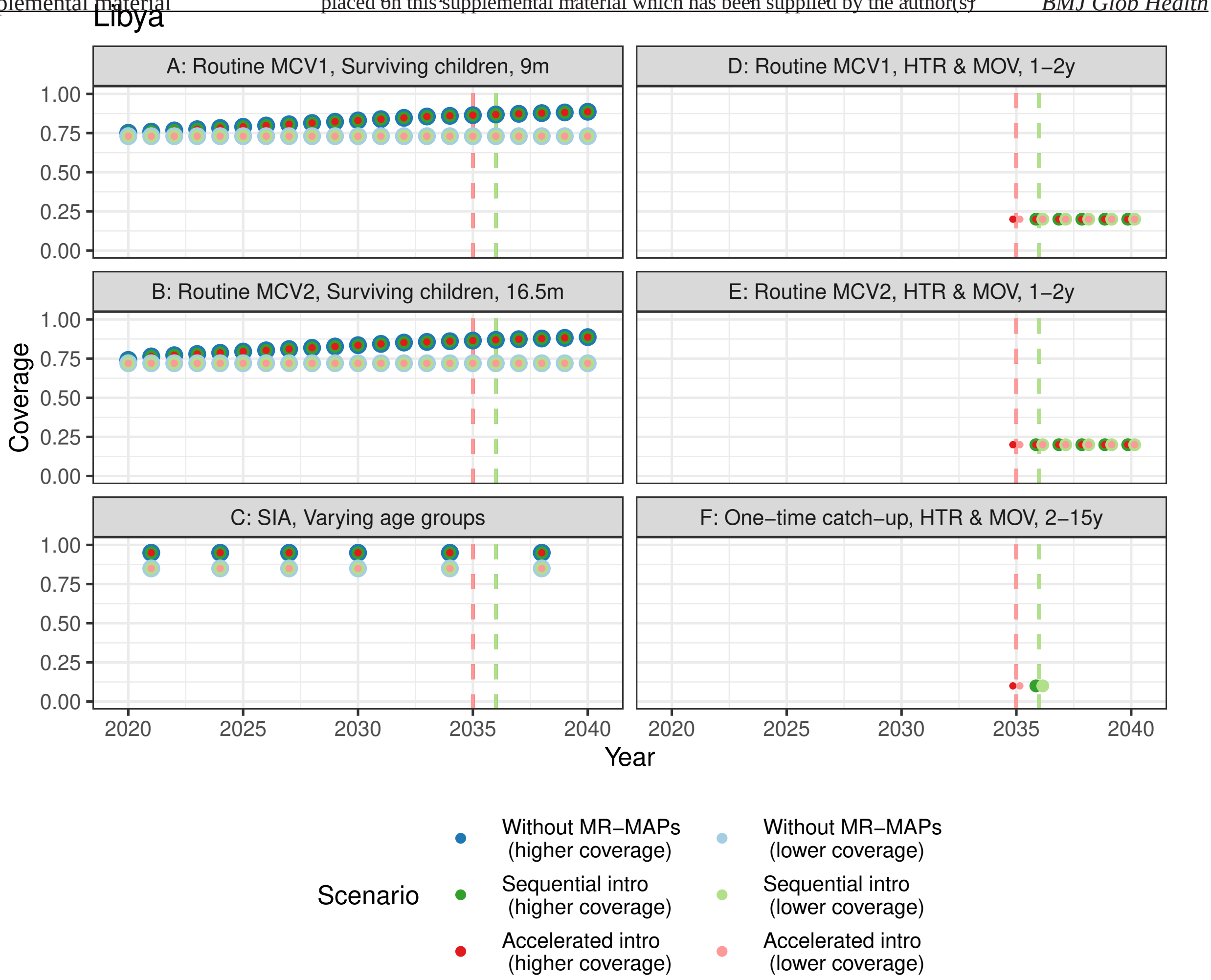
Libya
A: Routine MCV1, Surviving children, 9m
D: Routine MCV1, HTR & MOV, 1−2y
B: Routine MCV2, Surviving children, 16.5m
E: Routine MCV2, HTR & MOV, 1−2y
C: SIA, Varying age groups
F: One−time catch−up, HTR & MOV, 2−15y
Coverage
1.00
0.75
0.50
0.25
0.00
2020
2025
2030
2035
2040
Year
Scenario
Without MR−MAPs (higher coverage)
Sequential intro (higher coverage)
Accelerated intro (higher coverage)
Without MR−MAPs (lower coverage)
Sequential intro (lower coverage)
Accelerated intro (lower coverage)

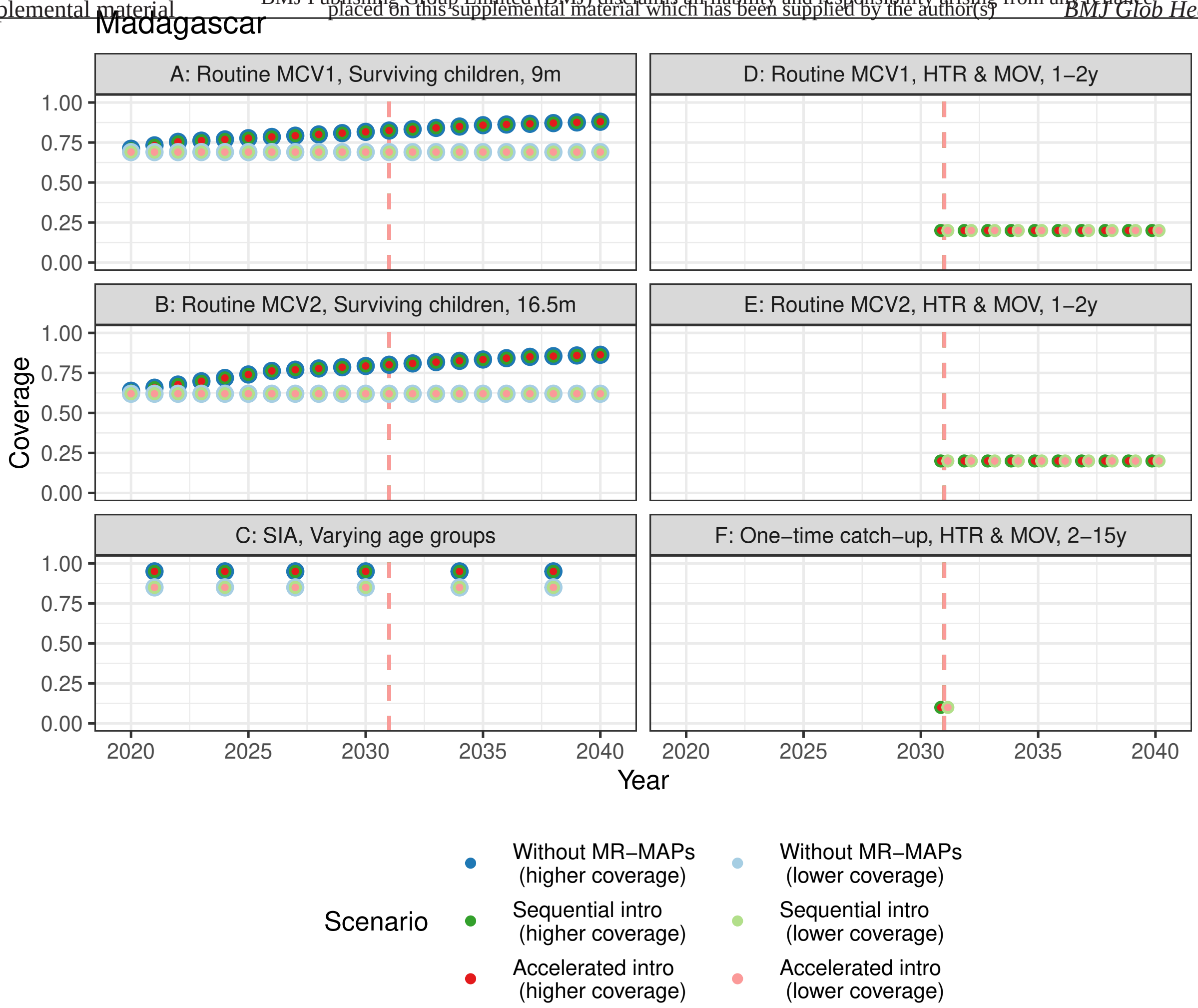
Madagascar
A: Routine MCV1, Surviving children, 9m
D: Routine MCV1, HTR & MOV, 1–2y
B: Routine MCV2, Surviving children, 16.5m
E: Routine MCV2, HTR & MOV, 1–2y
C: SIA, Varying age groups
F: One–time catch–up, HTR & MOV, 2–15y
Coverage
1.00
0.75
0.50
0.25
0.00
2020
2025
2030
2035
2040
Year
Scenario
Without MR–MAPs (higher coverage)
Sequential intro (higher coverage)
Accelerated intro (higher coverage)
Without MR–MAPs (lower coverage)
Sequential intro (lower coverage)
Accelerated intro (lower coverage)

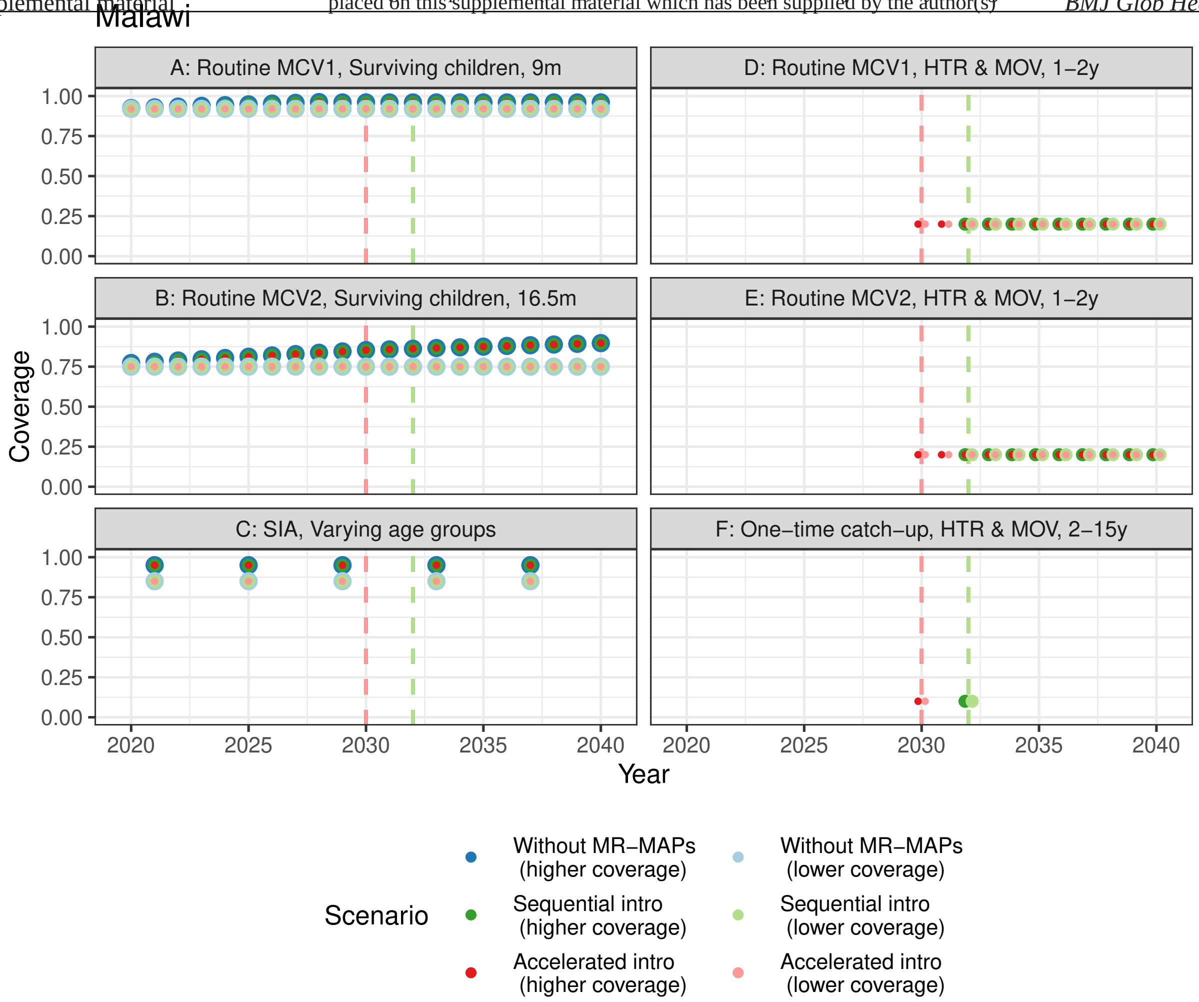
Malawi
A: Routine MCV1, Surviving children, 9m
D: Routine MCV1, HTR & MOV, 1–2y
B: Routine MCV2, Surviving children, 16.5m
E: Routine MCV2, HTR & MOV, 1–2y
C: SIA, Varying age groups
F: One–time catch–up, HTR & MOV, 2–15y
Coverage
1.00
0.75
0.50
0.25
0.00
2020
2025
2030
2035
2040
Year
Scenario
Without MR–MAPs (higher coverage)
Without MR–MAPs (lower coverage)
Sequential intro (higher coverage)
Sequential intro (lower coverage)
Accelerated intro (higher coverage)
Accelerated intro (lower coverage)

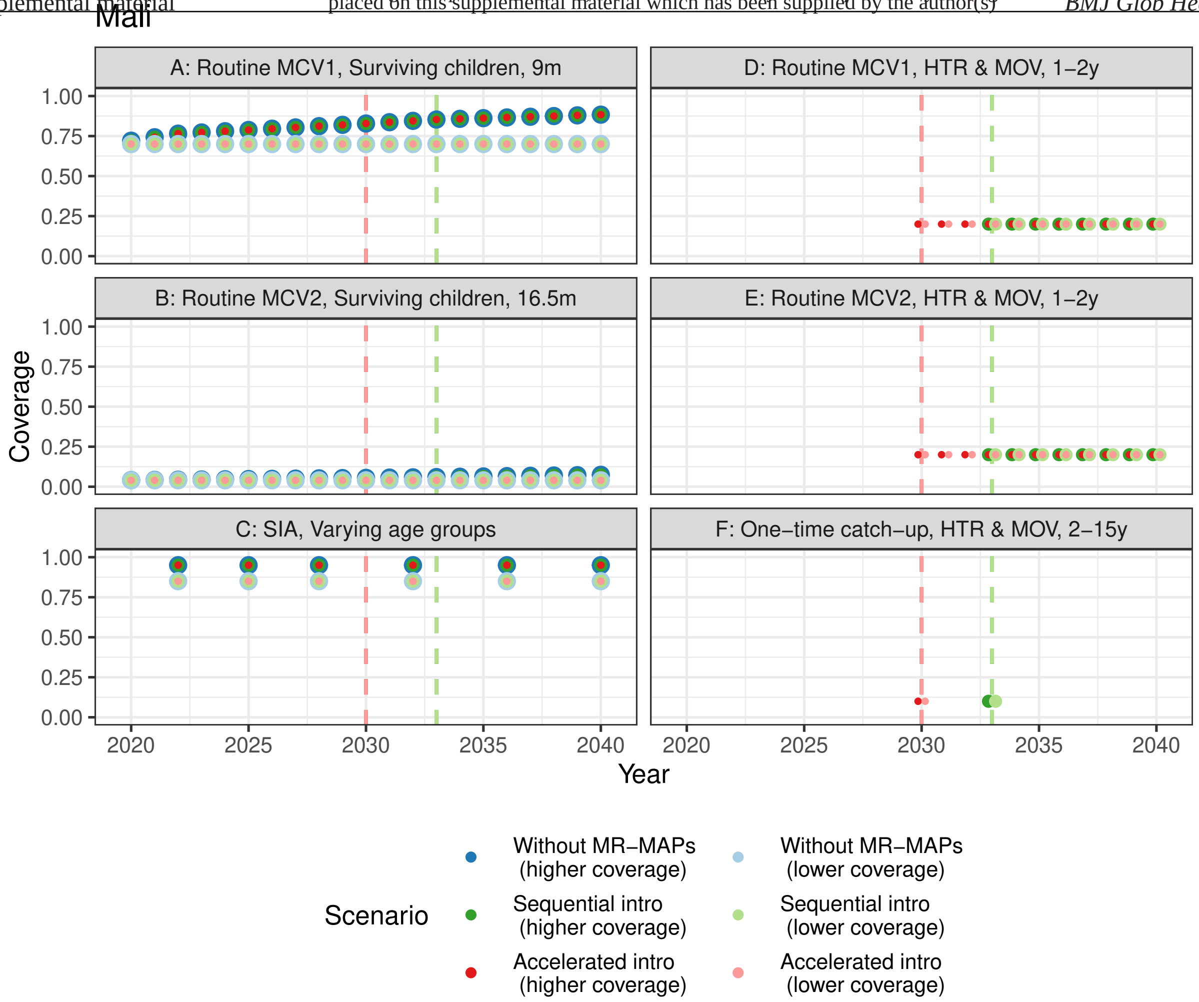
Mali
A: Routine MCV1, Surviving children, 9m
D: Routine MCV1, HTR & MOV, 1−2y
B: Routine MCV2, Surviving children, 16.5m
E: Routine MCV2, HTR & MOV, 1−2y
C: SIA, Varying age groups
F: One−time catch−up, HTR & MOV, 2−15y
Coverage
1.00
0.75
0.50
0.25
0.00
2020
2025
2030
2035
2040
Year
Scenario
Without MR−MAPs (higher coverage)
Sequential intro (higher coverage)
Accelerated intro (higher coverage)
Without MR−MAPs (lower coverage)
Sequential intro (lower coverage)
Accelerated intro (lower coverage)

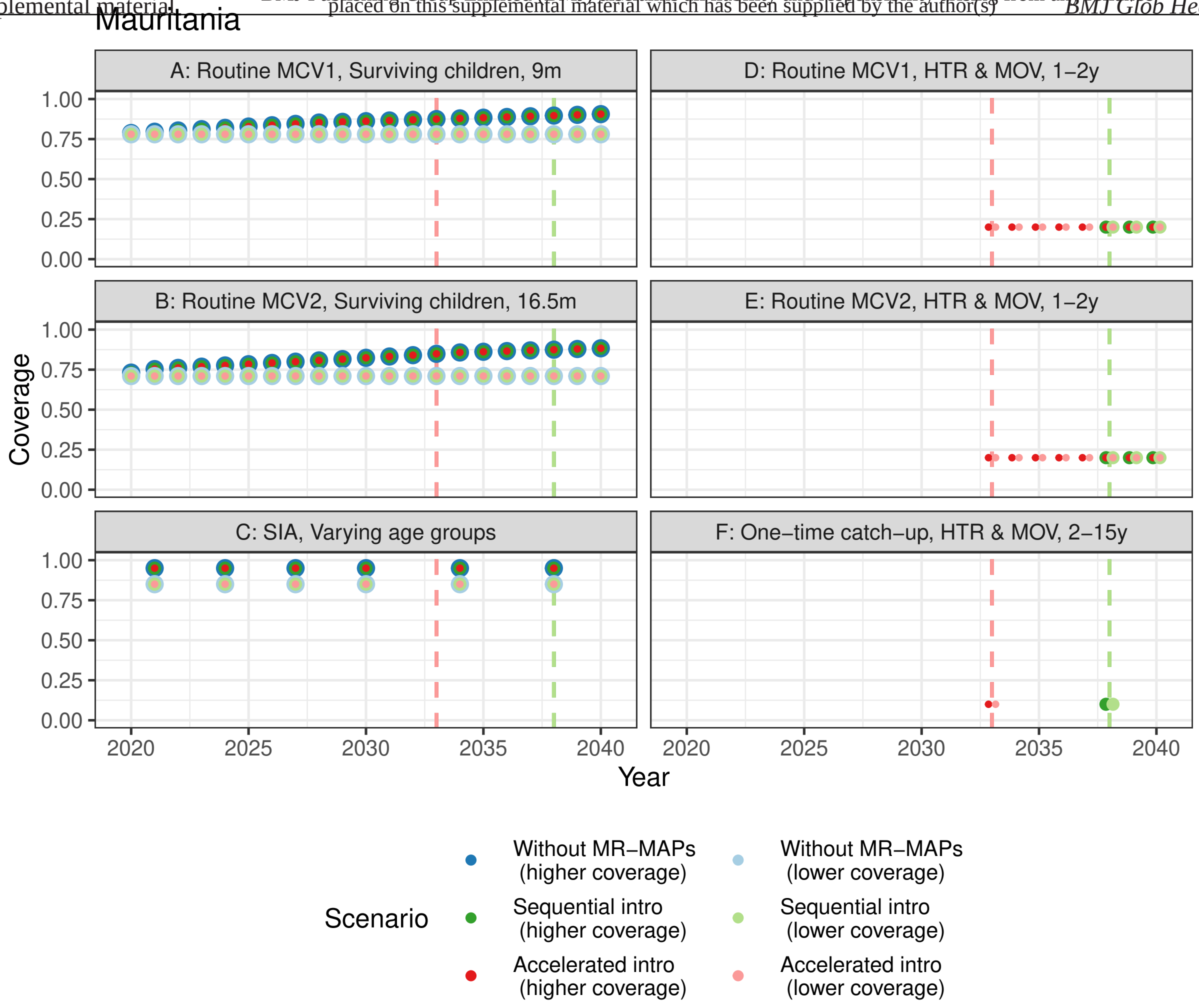
Mauritania
A: Routine MCV1, Surviving children, 9m
D: Routine MCV1, HTR & MOV, 1−2y
B: Routine MCV2, Surviving children, 16.5m
E: Routine MCV2, HTR & MOV, 1−2y
C: SIA, Varying age groups
F: One−time catch−up, HTR & MOV, 2−15y
Coverage
1.00
0.75
0.50
0.25
0.00
2020
2025
2030
2035
2040
Year
Scenario
Without MR−MAPs (higher coverage)
Without MR−MAPs (lower coverage)
Sequential intro (higher coverage)
Sequential intro (lower coverage)
Accelerated intro (higher coverage)
Accelerated intro (lower coverage)

Federated States of Micronesia

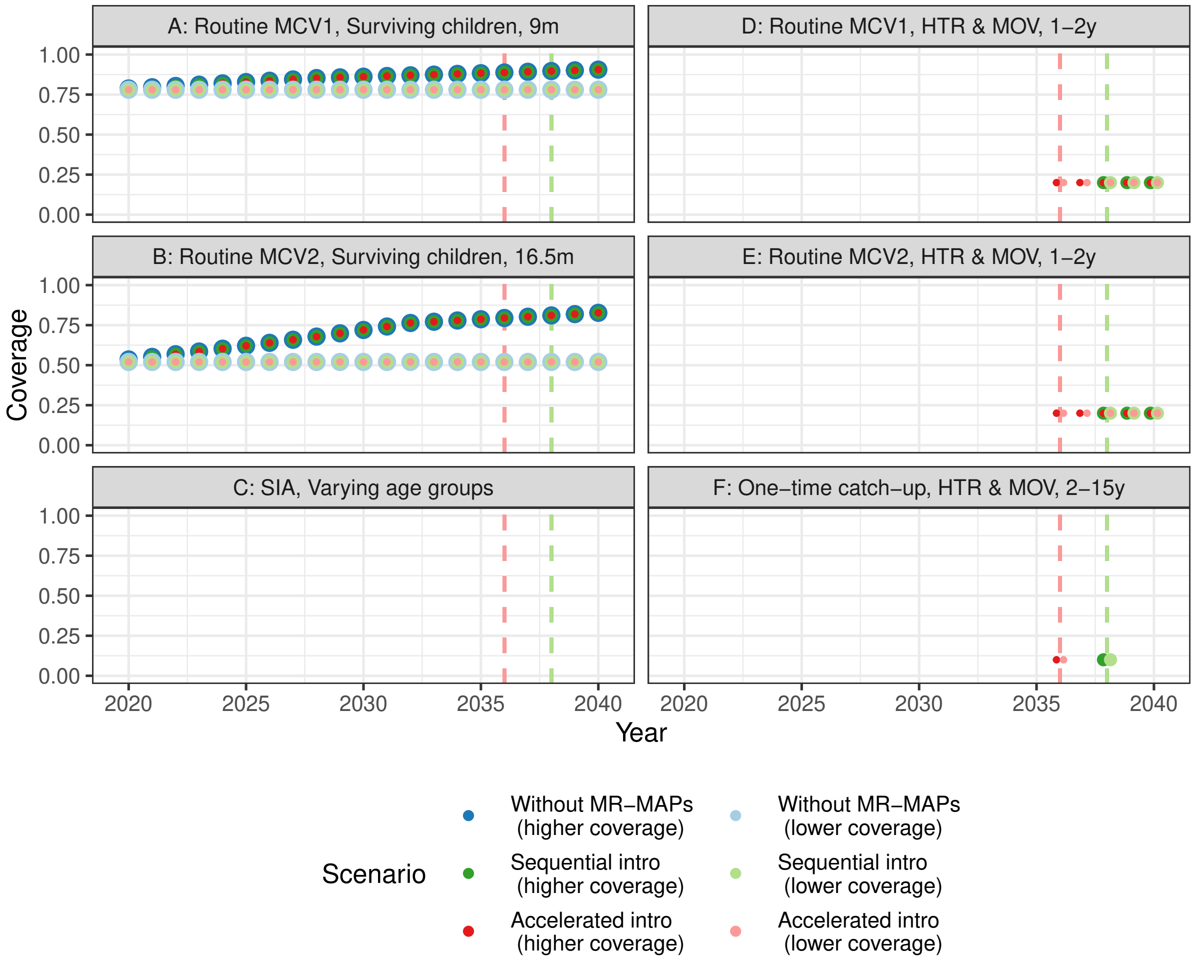

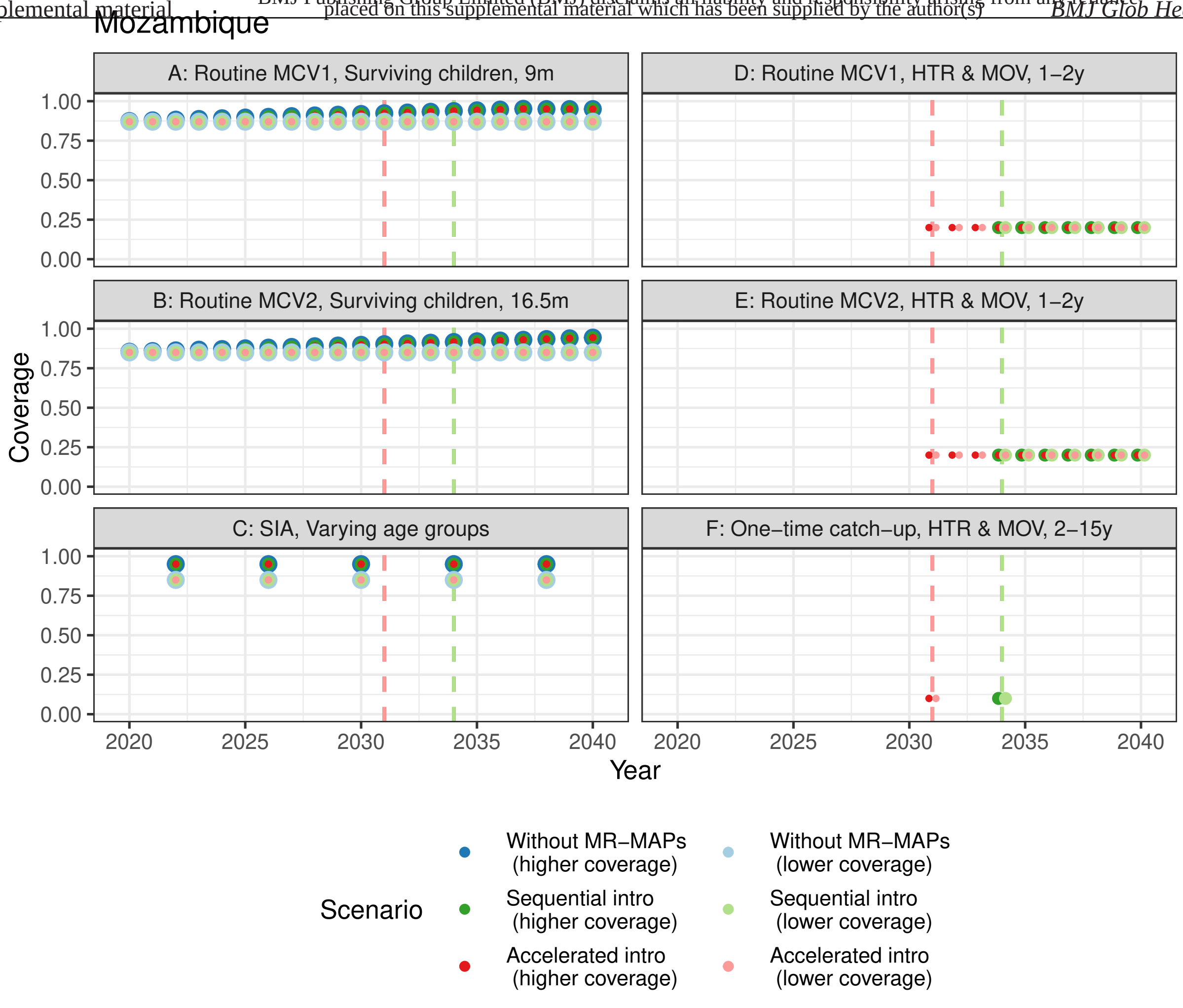
Mozambique
A: Routine MCV1, Surviving children, 9m
D: Routine MCV1, HTR & MOV, 1−2y
B: Routine MCV2, Surviving children, 16.5m
E: Routine MCV2, HTR & MOV, 1−2y
C: SIA, Varying age groups
F: One−time catch−up, HTR & MOV, 2−15y
Coverage
1.00
0.75
0.50
0.25
0.00
2020
2025
2030
2035
2040
Year
Scenario
Without MR−MAPs (higher coverage)
Sequential intro (higher coverage)
Accelerated intro (higher coverage)
Without MR−MAPs (lower coverage)
Sequential intro (lower coverage)
Accelerated intro (lower coverage)

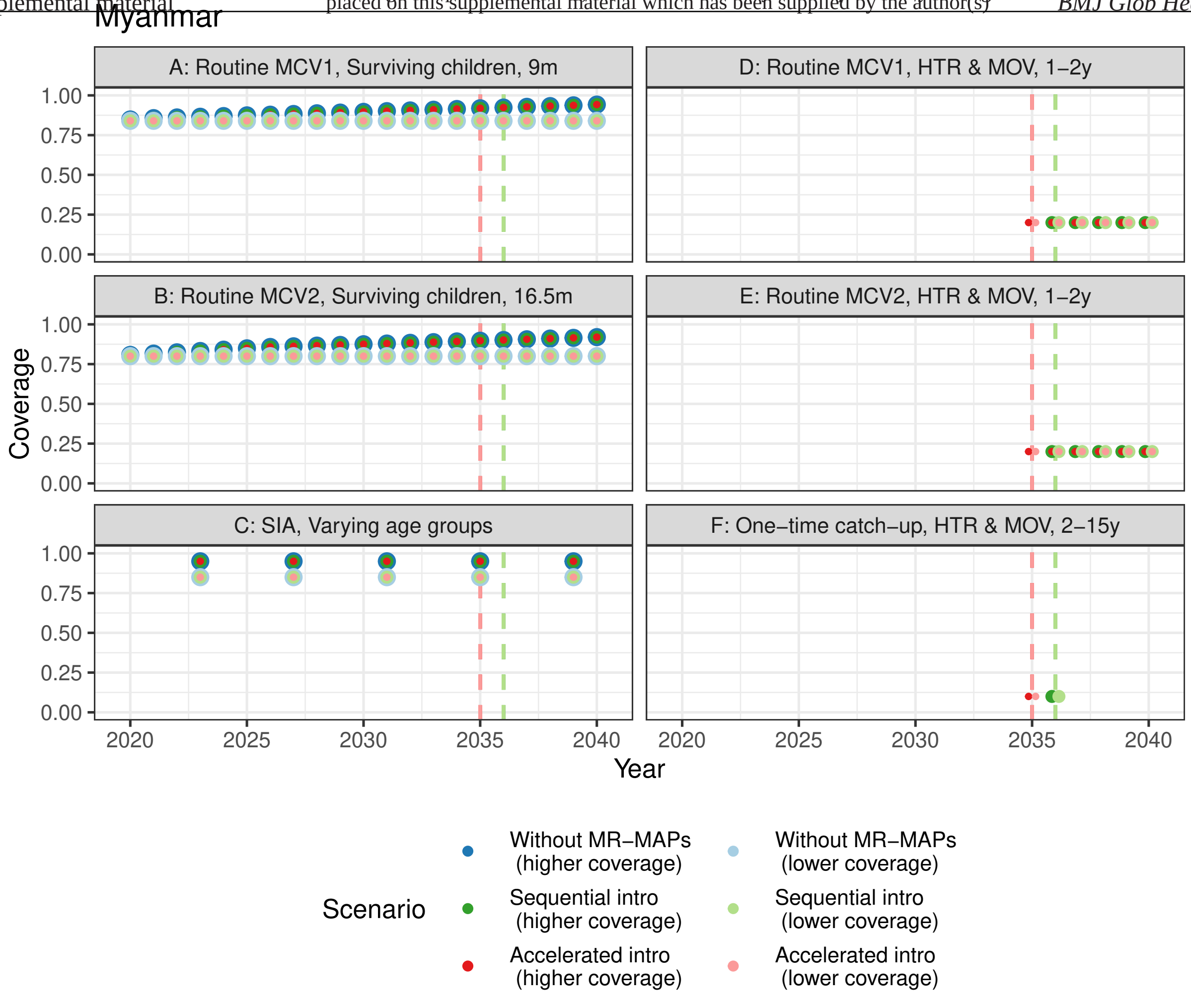
Myanmar
A: Routine MCV1, Surviving children, 9m
B: Routine MCV2, Surviving children, 16.5m
C: SIA, Varying age groups
D: Routine MCV1, HTR & MOV, 1−2y
E: Routine MCV2, HTR & MOV, 1−2y
F: One−time catch−up, HTR & MOV, 2−15y
Coverage
1.00
0.75
0.50
0.25
0.00
2020
2025
2030
2035
2040
Year
Scenario
Without MR−MAPs (higher coverage)
Sequential intro (higher coverage)
Accelerated intro (higher coverage)
Without MR−MAPs (lower coverage)
Sequential intro (lower coverage)
Accelerated intro (lower coverage)

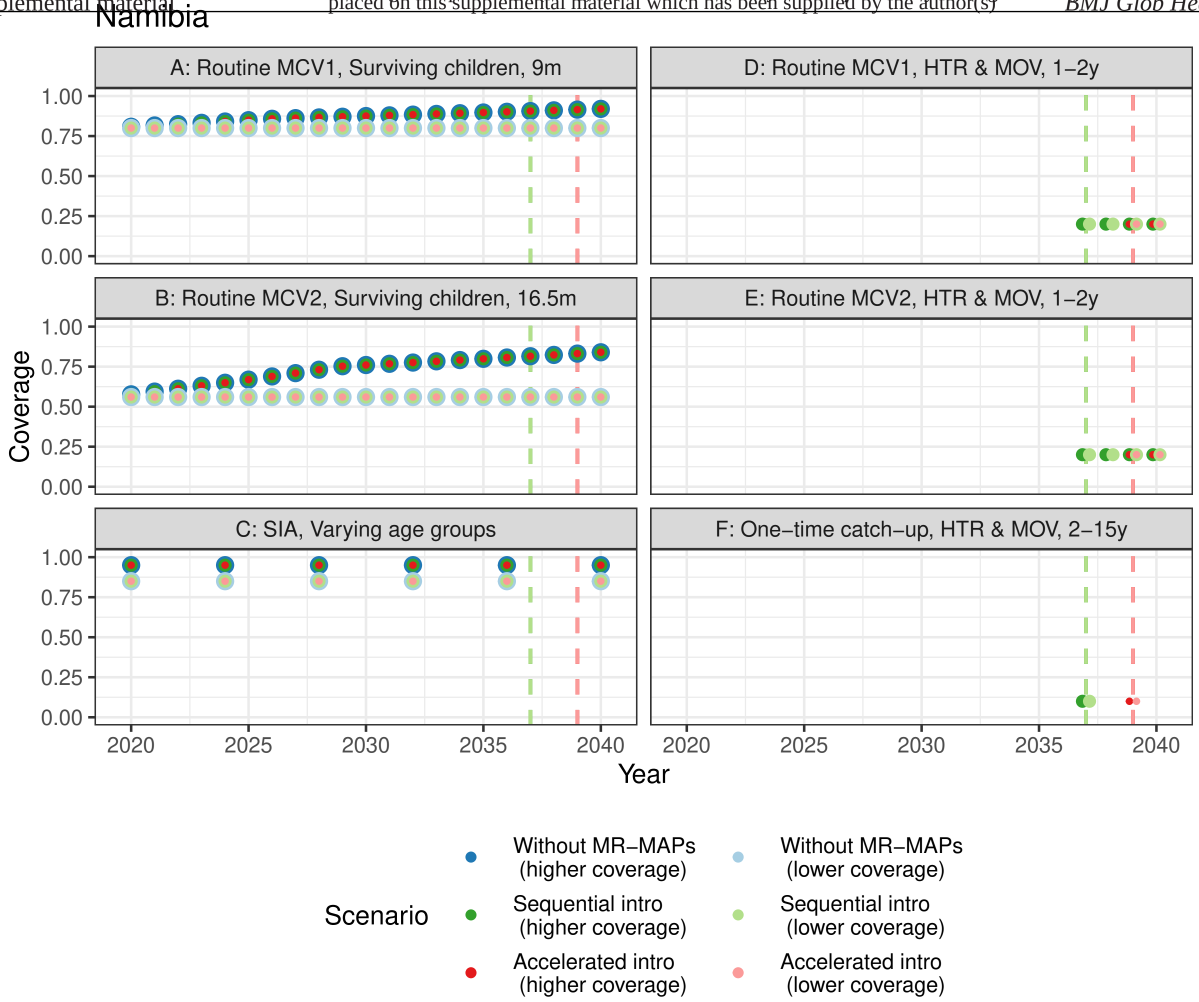
Namibia
A: Routine MCV1, Surviving children, 9m
B: Routine MCV2, Surviving children, 16.5m
C: SIA, Varying age groups
D: Routine MCV1, HTR & MOV, 1–2y
E: Routine MCV2, HTR & MOV, 1–2y
F: One–time catch–up, HTR & MOV, 2–15y
Coverage
1.00
0.75
0.50
0.25
0.00
2020
2025
2030
2035
2040
Year
Scenario
Without MR–MAPs (higher coverage)
Sequential intro (higher coverage)
Accelerated intro (higher coverage)
Without MR–MAPs (lower coverage)
Sequential intro (lower coverage)
Accelerated intro (lower coverage)

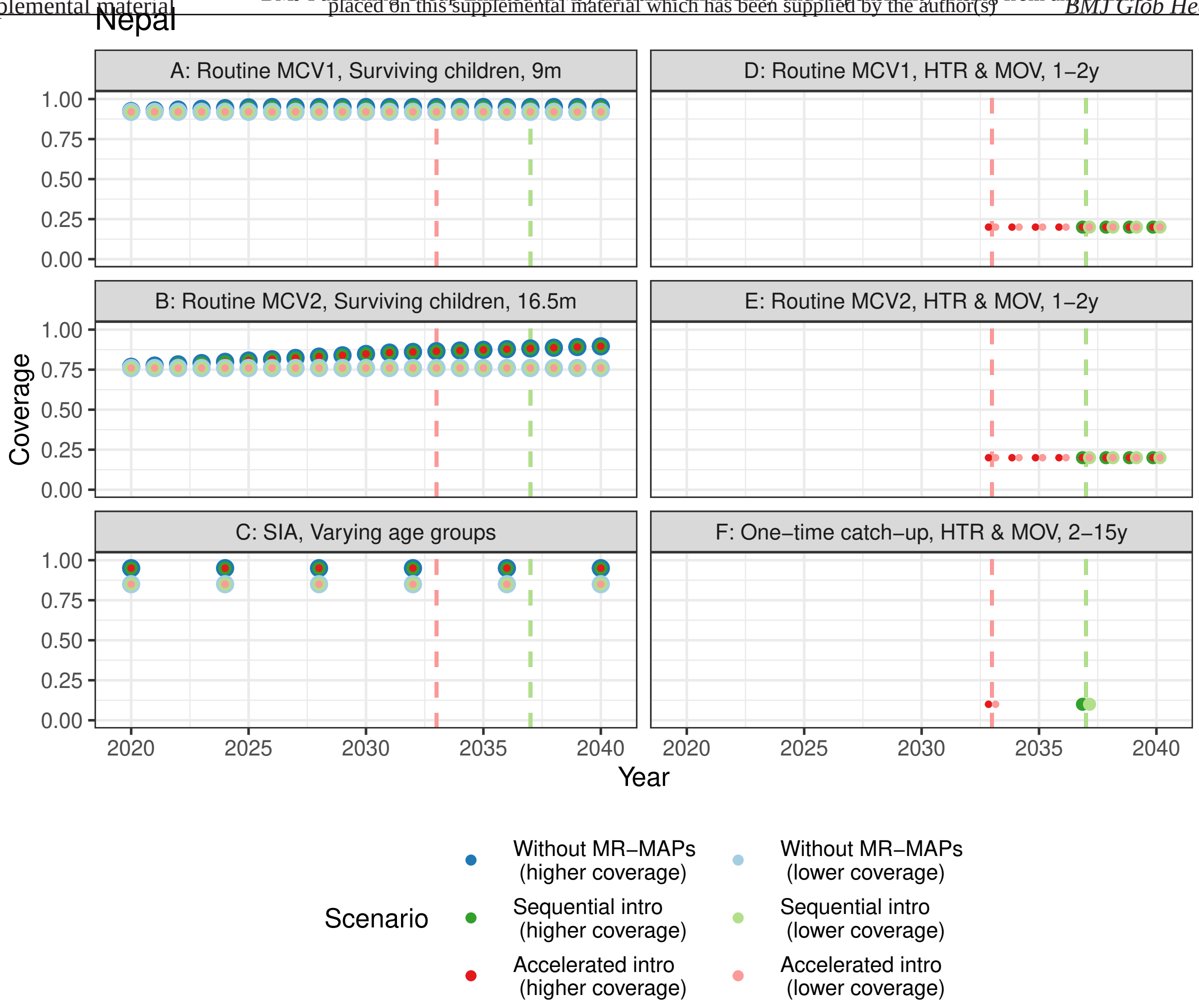
Nepal
A: Routine MCV1, Surviving children, 9m
D: Routine MCV1, HTR & MOV, 1−2y
B: Routine MCV2, Surviving children, 16.5m
E: Routine MCV2, HTR & MOV, 1−2y
C: SIA, Varying age groups
F: One−time catch−up, HTR & MOV, 2−15y
Coverage
1.00
0.75
0.50
0.25
0.00
2020
2025
2030
2035
2040
Year
Scenario
Without MR−MAPs (higher coverage)
Without MR−MAPs (lower coverage)
Sequential intro (higher coverage)
Sequential intro (lower coverage)
Accelerated intro (higher coverage)
Accelerated intro (lower coverage)

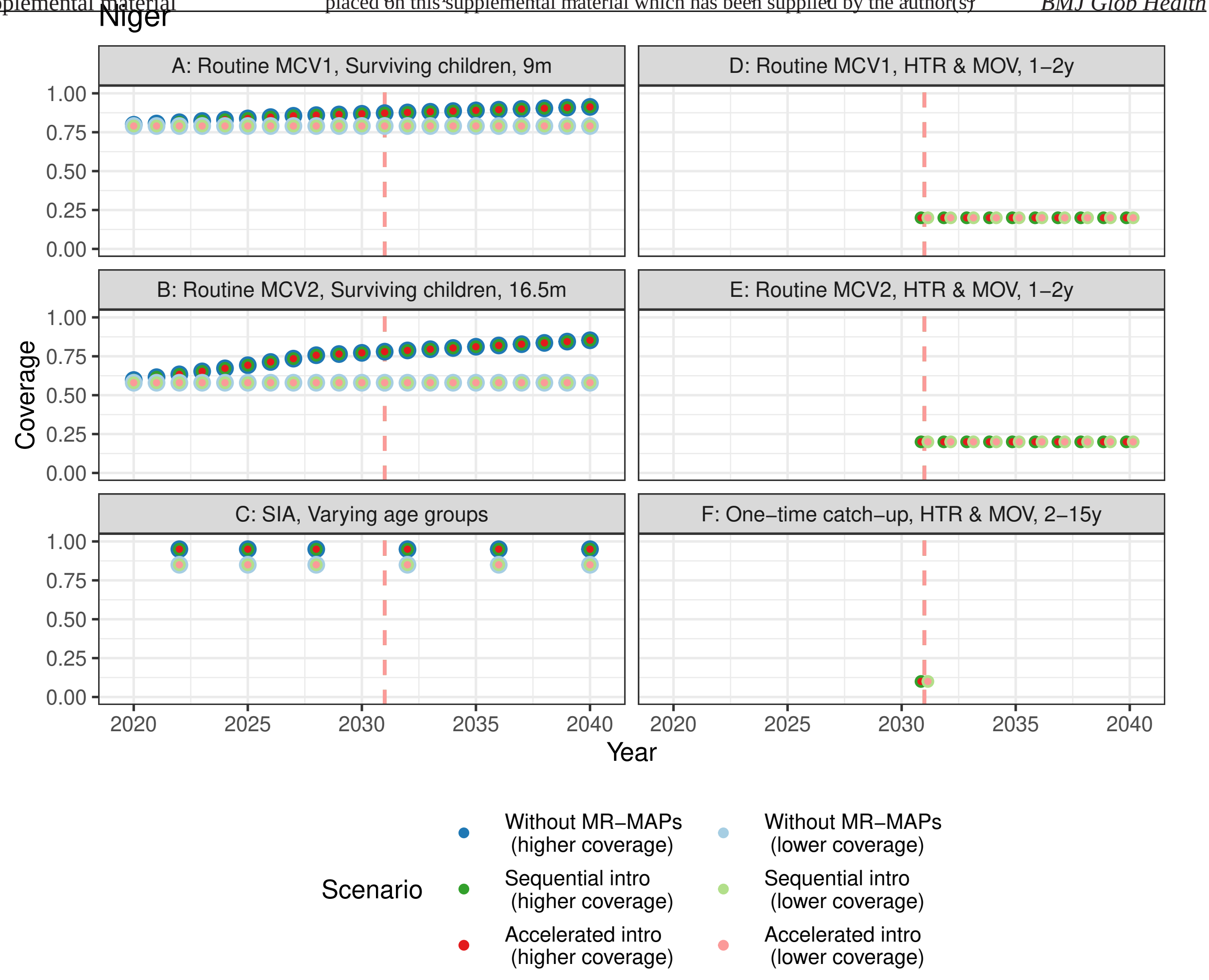
Niger
A: Routine MCV1, Surviving children, 9m
D: Routine MCV1, HTR & MOV, 1−2y
B: Routine MCV2, Surviving children, 16.5m
E: Routine MCV2, HTR & MOV, 1−2y
C: SIA, Varying age groups
F: One−time catch−up, HTR & MOV, 2−15y
Coverage
1.00
0.75
0.50
0.25
0.00
2020
2025
2030
2035
2040
Year
Scenario
Without MR−MAPs (higher coverage)
Sequential intro (higher coverage)
Accelerated intro (higher coverage)
Without MR−MAPs (lower coverage)
Sequential intro (lower coverage)
Accelerated intro (lower coverage)

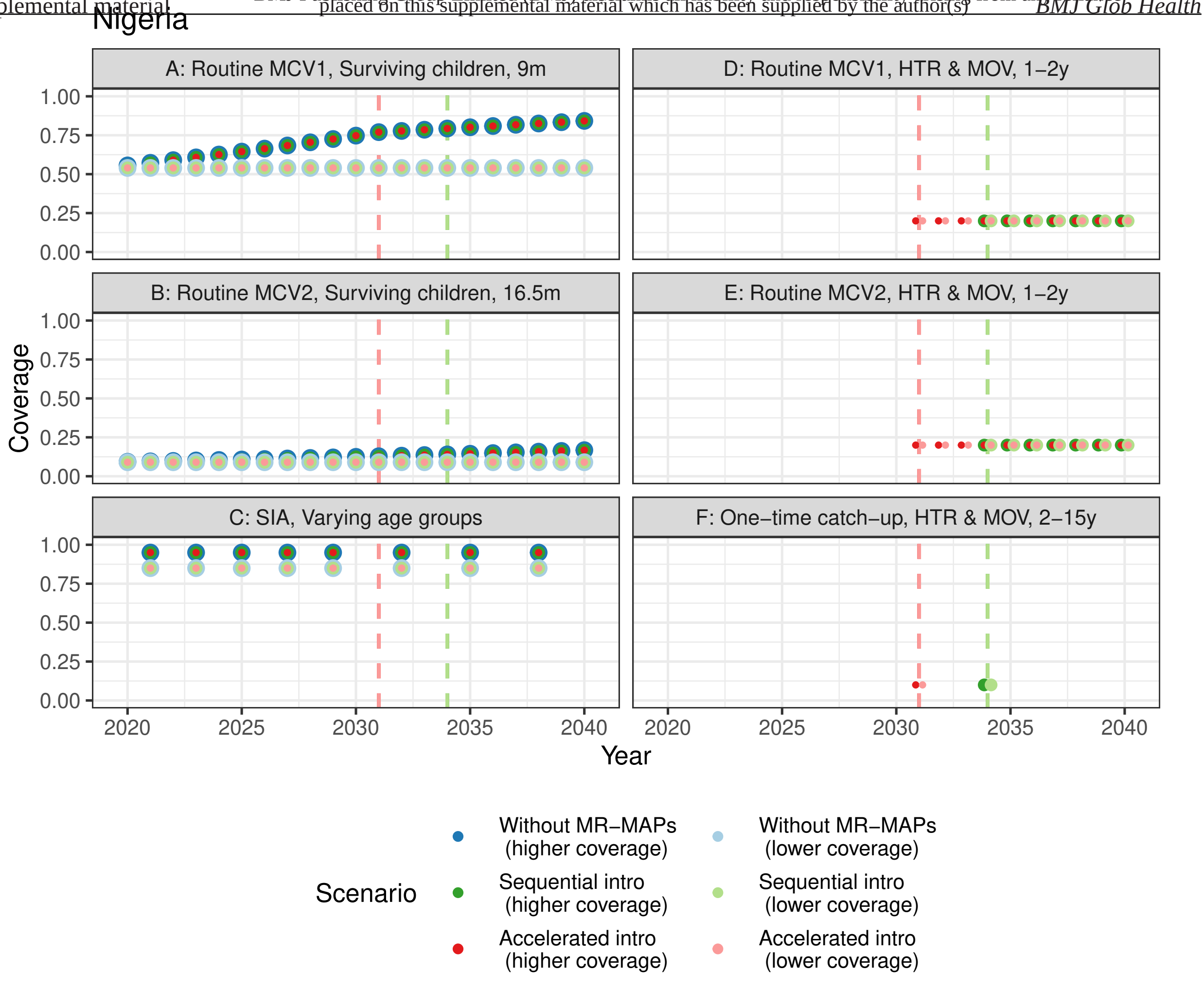
Nigeria
A: Routine MCV1, Surviving children, 9m
D: Routine MCV1, HTR & MOV, 1–2y
B: Routine MCV2, Surviving children, 16.5m
E: Routine MCV2, HTR & MOV, 1–2y
C: SIA, Varying age groups
F: One–time catch–up, HTR & MOV, 2–15y
Coverage
1.00
0.75
0.50
0.25
0.00
2020
2025
2030
2035
2040
Year
Scenario
Without MR–MAPs (higher coverage)
Sequential intro (higher coverage)
Accelerated intro (higher coverage)
Without MR–MAPs (lower coverage)
Sequential intro (lower coverage)
Accelerated intro (lower coverage)

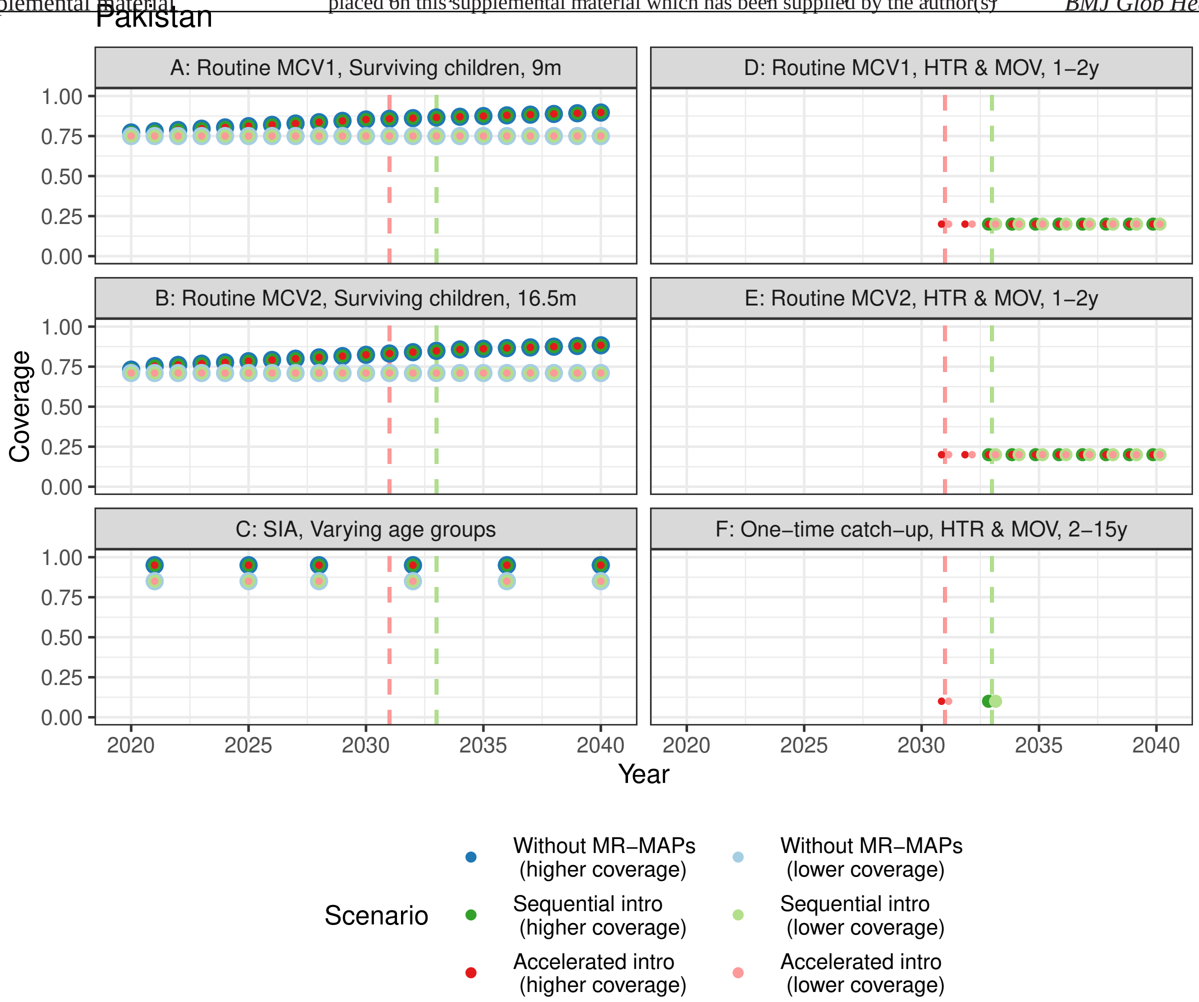
Pakistan
A: Routine MCV1, Surviving children, 9m
B: Routine MCV2, Surviving children, 16.5m
C: SIA, Varying age groups
D: Routine MCV1, HTR & MOV, 1−2y
E: Routine MCV2, HTR & MOV, 1−2y
F: One−time catch−up, HTR & MOV, 2−15y
Coverage
1.00
0.75
0.50
0.25
0.00
2020
2025
2030
2035
2040
Year
Scenario
Without MR−MAPs (higher coverage)
Without MR−MAPs (lower coverage)
Sequential intro (higher coverage)
Sequential intro (lower coverage)
Accelerated intro (higher coverage)
Accelerated intro (lower coverage)

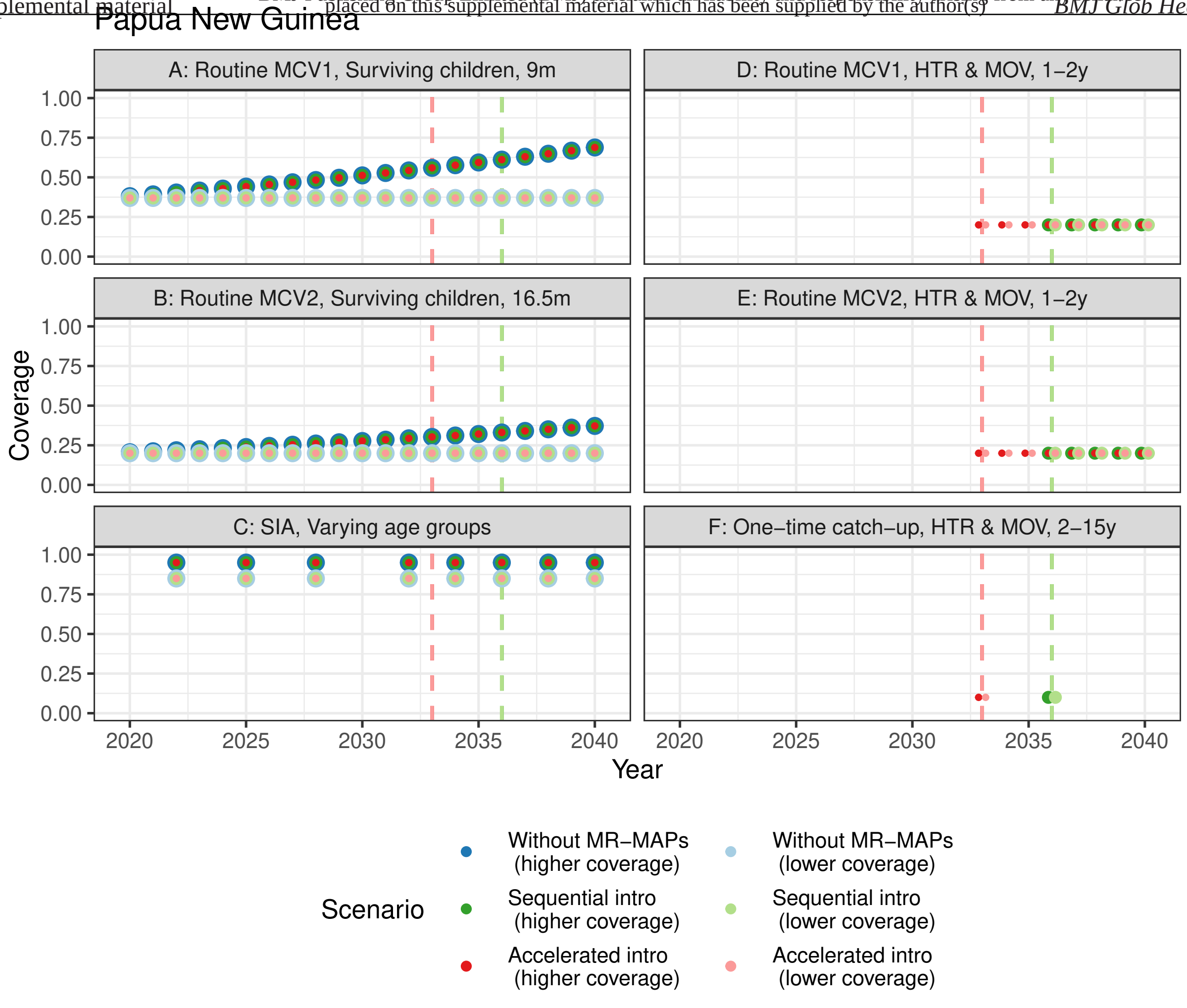
Papua New Guinea
A: Routine MCV1, Surviving children, 9m
D: Routine MCV1, HTR & MOV, 1−2y
B: Routine MCV2, Surviving children, 16.5m
E: Routine MCV2, HTR & MOV, 1−2y
C: SIA, Varying age groups
F: One−time catch−up, HTR & MOV, 2−15y
Coverage
1.00
0.75
0.50
0.25
0.00
2020
2025
2030
2035
2040
Year
Scenario
Without MR−MAPs (higher coverage)
Without MR−MAPs (lower coverage)
Sequential intro (higher coverage)
Sequential intro (lower coverage)
Accelerated intro (higher coverage)
Accelerated intro (lower coverage)

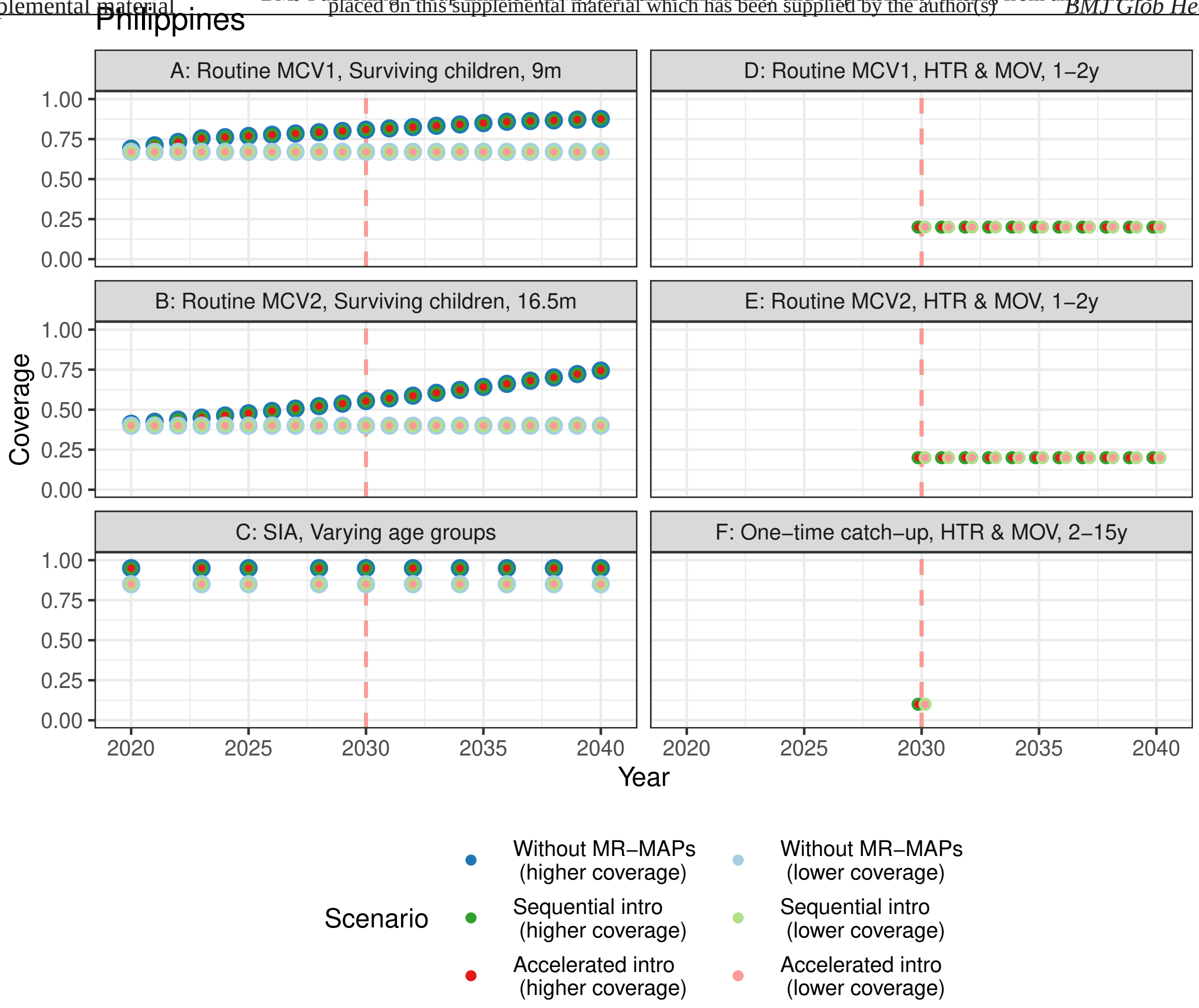

Philippines
A: Routine MCV1, Surviving children, 9m
D: Routine MCV1, HTR & MOV, 1−2y
B: Routine MCV2, Surviving children, 16.5m
E: Routine MCV2, HTR & MOV, 1−2y
C: SIA, Varying age groups
F: One−time catch−up, HTR & MOV, 2−15y
Coverage
1.00
0.75
0.50
0.25
0.00
2020
2025
2030
2035
2040
Year
Scenario
Without MR−MAPs (higher coverage)
Sequential intro (higher coverage)
Accelerated intro (higher coverage)
Without MR−MAPs (lower coverage)
Sequential intro (lower coverage)
Accelerated intro (lower coverage)

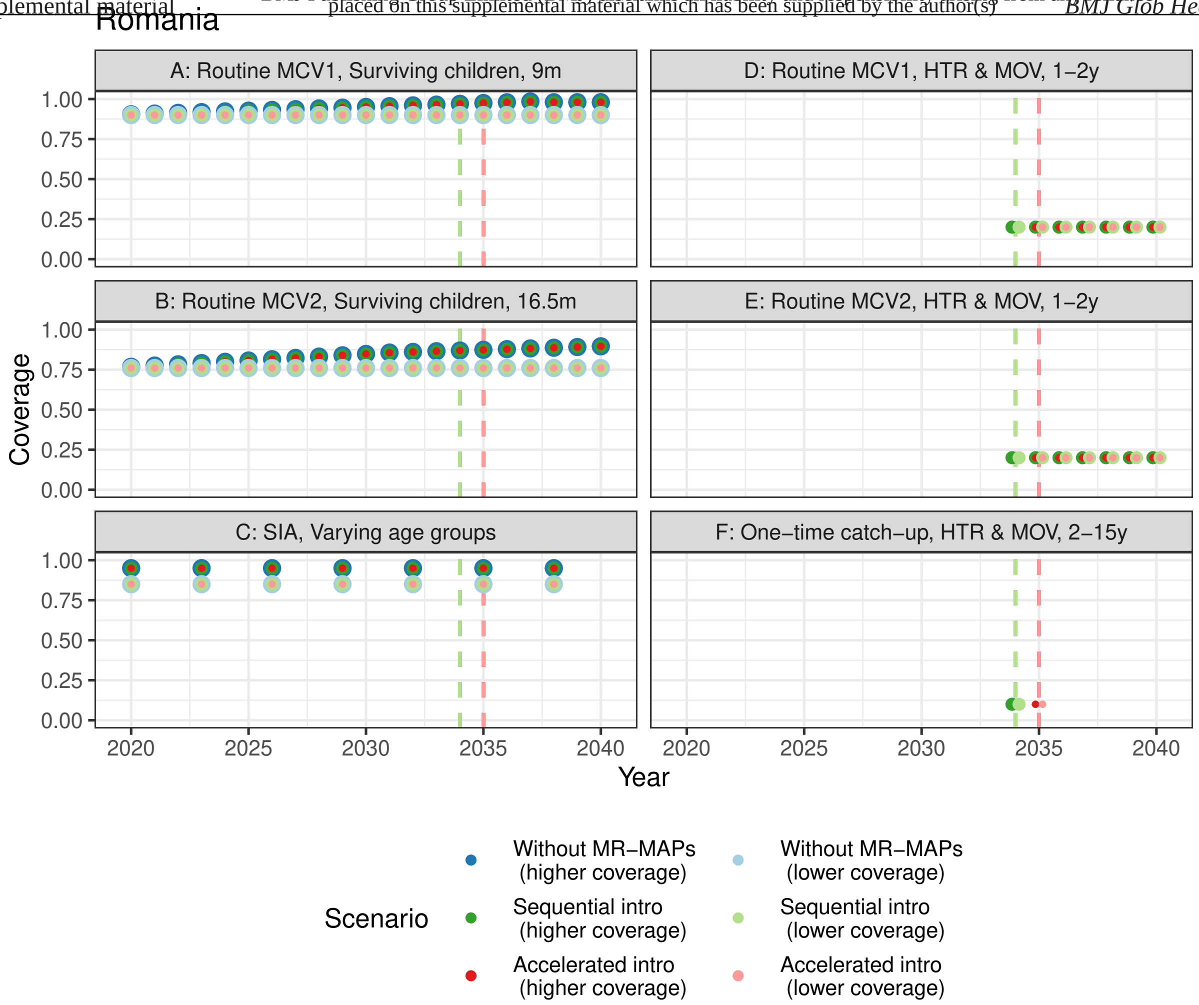
Romania
A: Routine MCV1, Surviving children, 9m
D: Routine MCV1, HTR & MOV, 1–2y
B: Routine MCV2, Surviving children, 16.5m
E: Routine MCV2, HTR & MOV, 1–2y
C: SIA, Varying age groups
F: One–time catch–up, HTR & MOV, 2–15y
Coverage
1.00
0.75
0.50
0.25
0.00
2020
2025
2030
2035
2040
Year
Scenario
Without MR–MAPs (higher coverage)
Without MR–MAPs (lower coverage)
Sequential intro (higher coverage)
Sequential intro (lower coverage)
Accelerated intro (higher coverage)
Accelerated intro (lower coverage)

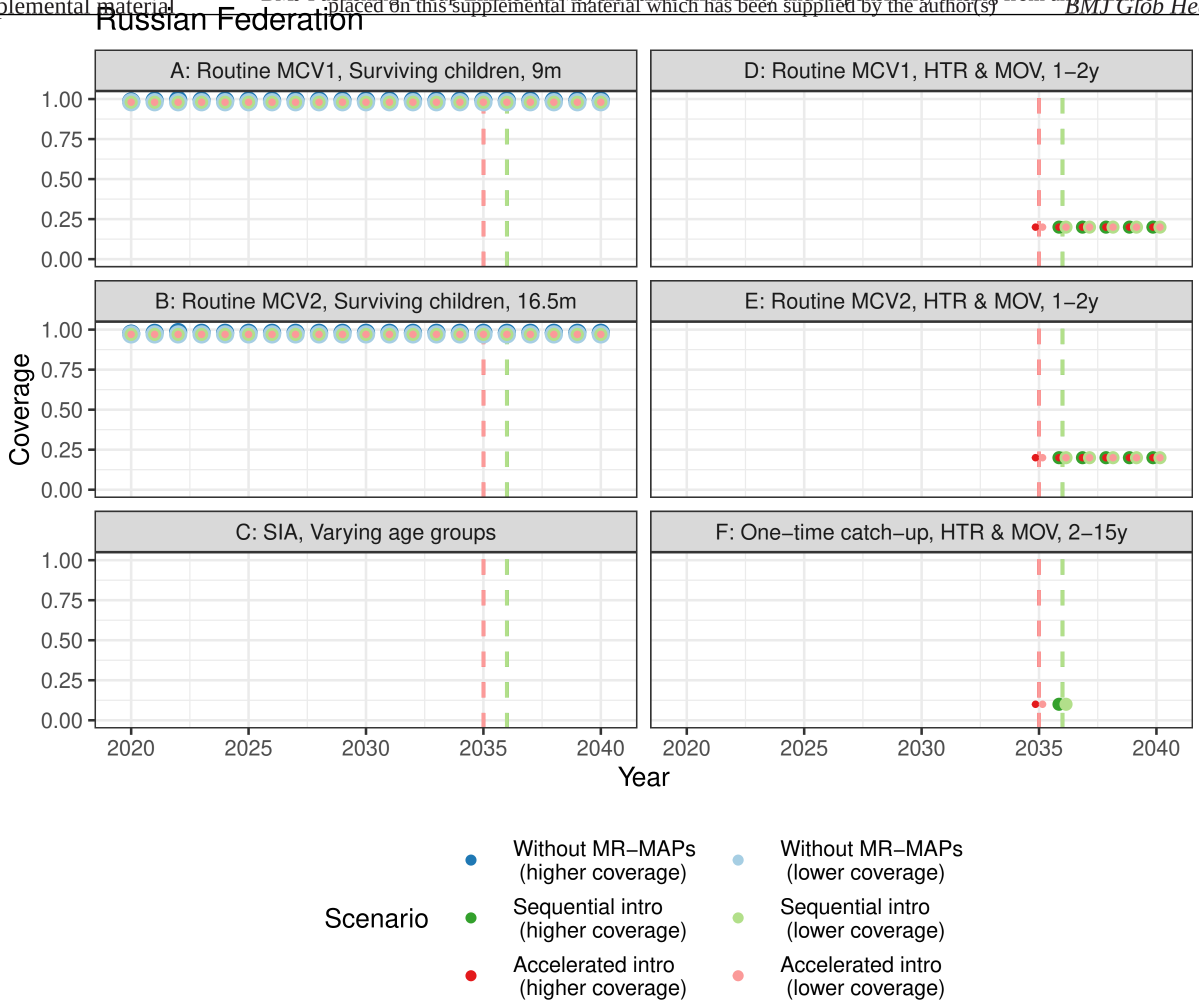
Russian Federation
A: Routine MCV1, Surviving children, 9m
D: Routine MCV1, HTR & MOV, 1−2y
B: Routine MCV2, Surviving children, 16.5m
E: Routine MCV2, HTR & MOV, 1−2y
C: SIA, Varying age groups
F: One−time catch−up, HTR & MOV, 2−15y
Coverage
1.00
0.75
0.50
0.25
0.00
2020
2025
2030
2035
2040
Year
Scenario
Without MR−MAPs (higher coverage)
Without MR−MAPs (lower coverage)
Sequential intro (higher coverage)
Sequential intro (lower coverage)
Accelerated intro (higher coverage)
Accelerated intro (lower coverage)

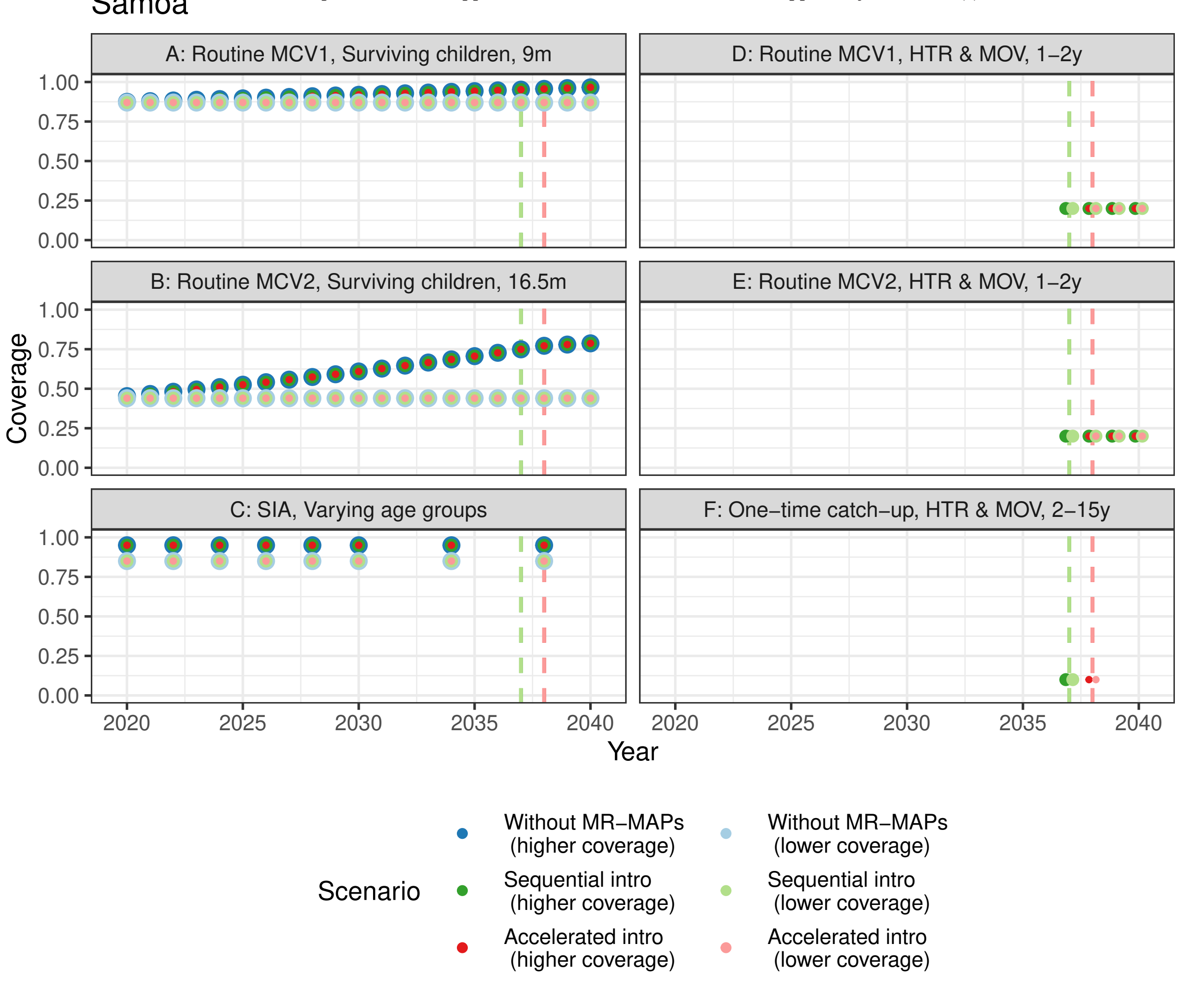
Samoa
A: Routine MCV1, Surviving children, 9m
D: Routine MCV1, HTR & MOV, 1−2y
B: Routine MCV2, Surviving children, 16.5m
E: Routine MCV2, HTR & MOV, 1−2y
C: SIA, Varying age groups
F: One−time catch−up, HTR & MOV, 2−15y
Coverage
1.00
0.75
0.50
0.25
0.00
2020
2025
2030
2035
2040
Year
Scenario
Without MR−MAPs (higher coverage)
Without MR−MAPs (lower coverage)
Sequential intro (higher coverage)
Sequential intro (lower coverage)
Accelerated intro (higher coverage)
Accelerated intro (lower coverage)

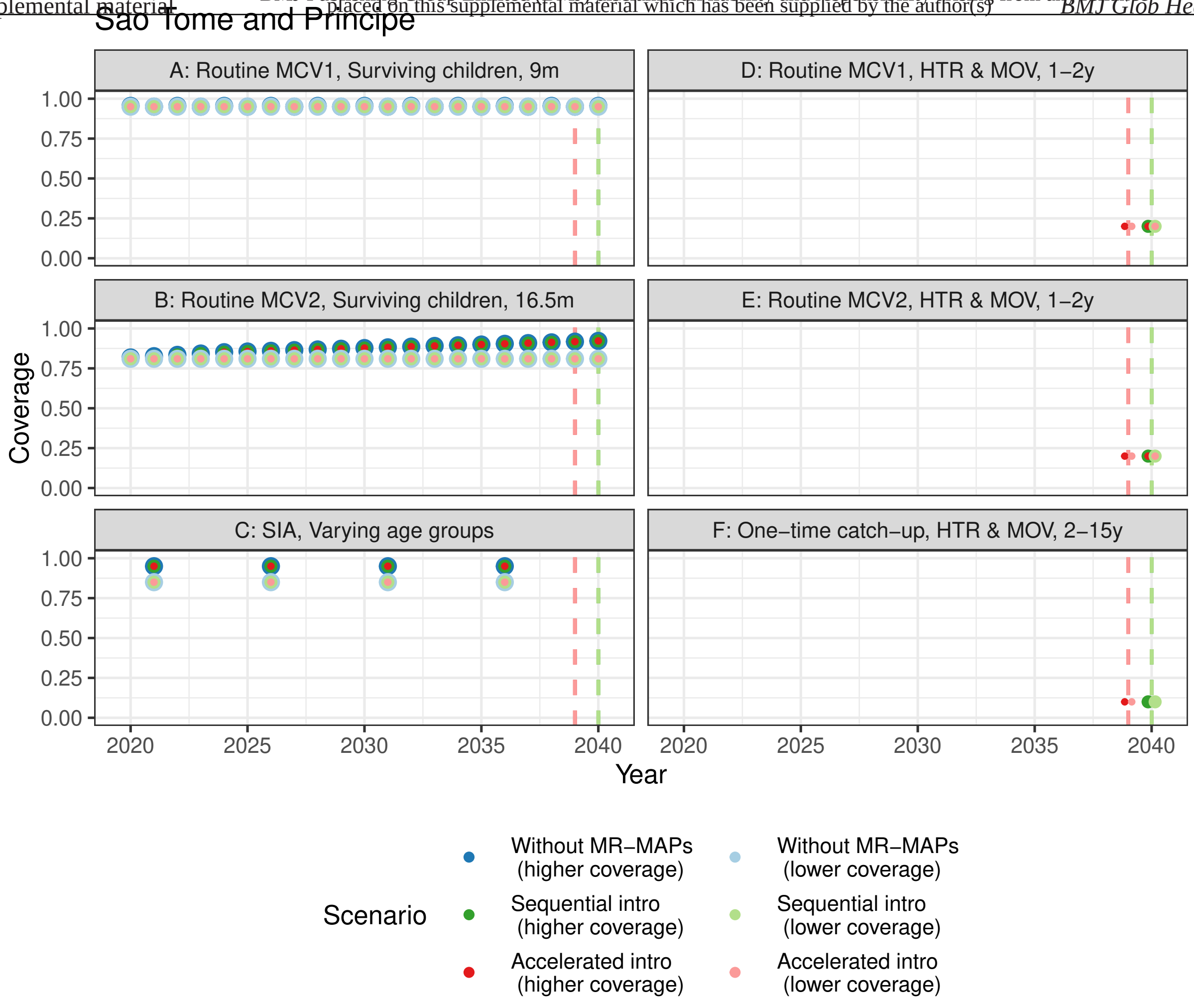
Sao Tome and Principe
A: Routine MCV1, Surviving children, 9m
D: Routine MCV1, HTR & MOV, 1–2y
B: Routine MCV2, Surviving children, 16.5m
E: Routine MCV2, HTR & MOV, 1–2y
C: SIA, Varying age groups
F: One–time catch–up, HTR & MOV, 2–15y
Coverage
1.00
0.75
0.50
0.25
0.00
2020
2025
2030
2035
2040
Year
Scenario
Without MR–MAPs (higher coverage)
Without MR–MAPs (lower coverage)
Sequential intro (higher coverage)
Sequential intro (lower coverage)
Accelerated intro (higher coverage)
Accelerated intro (lower coverage)

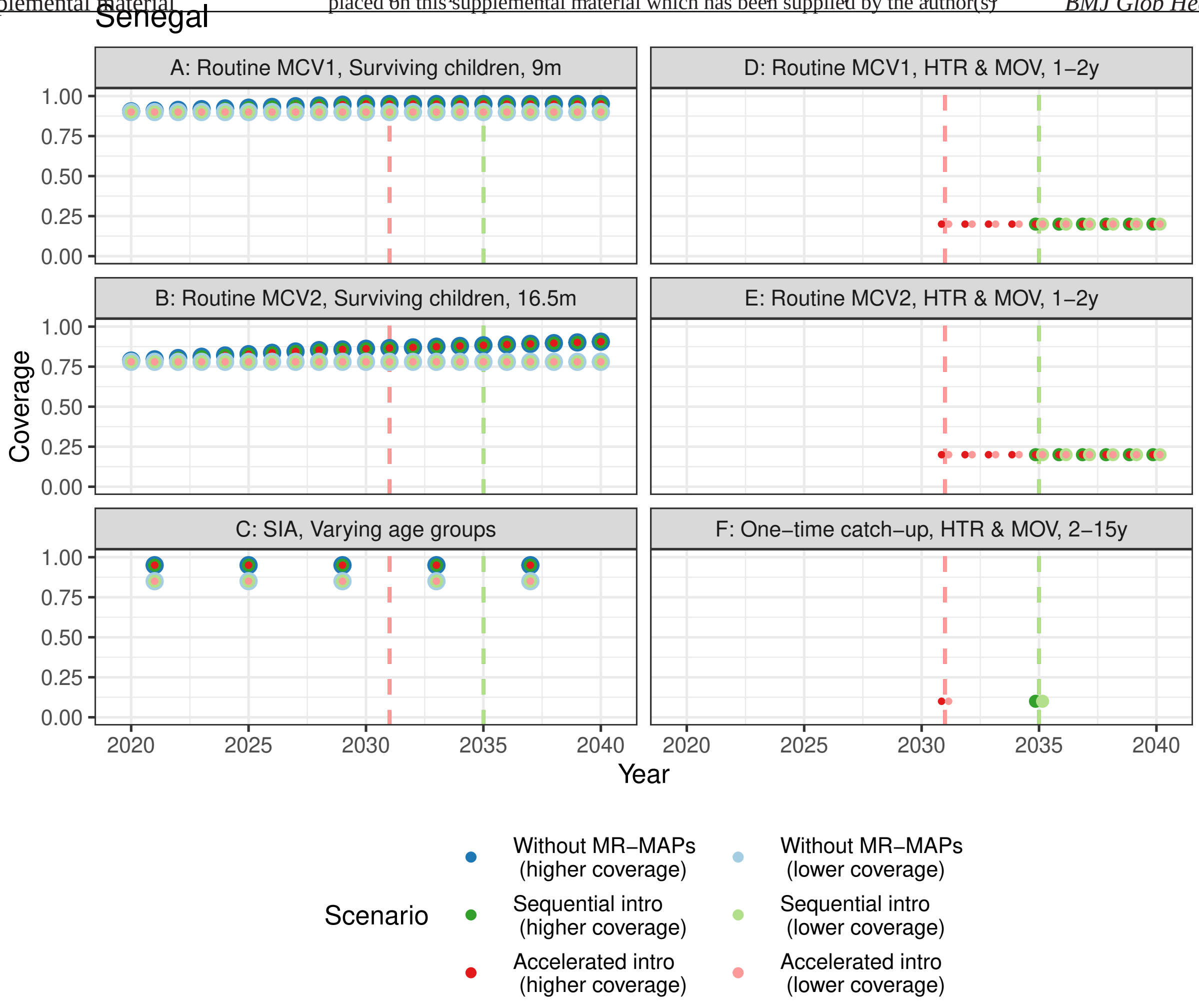
Senegal
A: Routine MCV1, Surviving children, 9m
B: Routine MCV2, Surviving children, 16.5m
C: SIA, Varying age groups
D: Routine MCV1, HTR & MOV, 1−2y
E: Routine MCV2, HTR & MOV, 1−2y
F: One−time catch−up, HTR & MOV, 2−15y
Coverage
1.00
0.75
0.50
0.25
0.00
2020
2025
2030
2035
2040
Year
Scenario
Without MR−MAPs (higher coverage)
Sequential intro (higher coverage)
Accelerated intro (higher coverage)
Without MR−MAPs (lower coverage)
Sequential intro (lower coverage)
Accelerated intro (lower coverage)

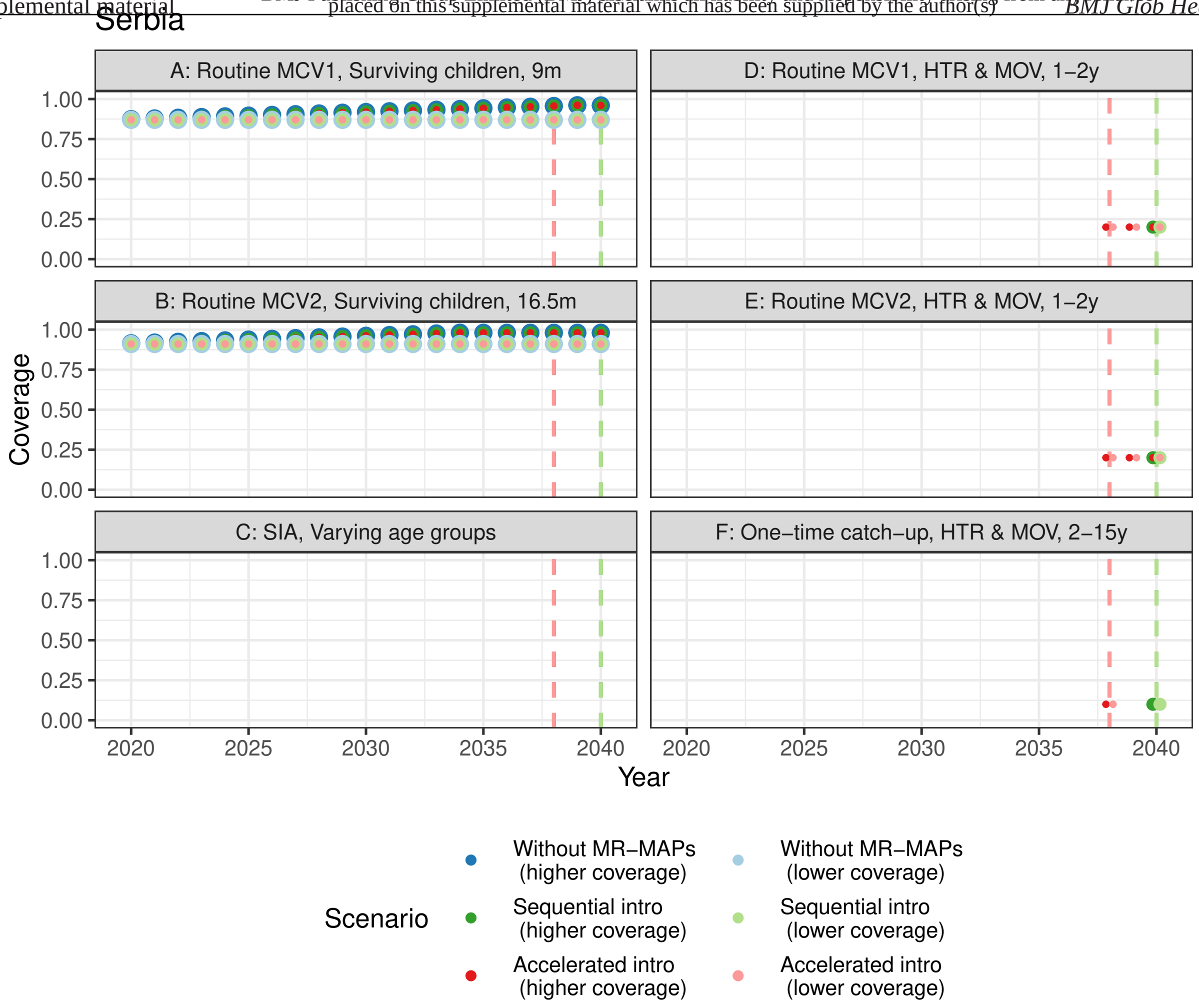
Serbia
A: Routine MCV1, Surviving children, 9m
B: Routine MCV2, Surviving children, 16.5m
C: SIA, Varying age groups
D: Routine MCV1, HTR & MOV, 1−2y
E: Routine MCV2, HTR & MOV, 1−2y
F: One−time catch−up, HTR & MOV, 2−15y
Coverage
1.00
0.75
0.50
0.25
0.00
2020
2025
2030
2035
2040
Year
Scenario
Without MR−MAPs (higher coverage)
Without MR−MAPs (lower coverage)
Sequential intro (higher coverage)
Sequential intro (lower coverage)
Accelerated intro (higher coverage)
Accelerated intro (lower coverage)

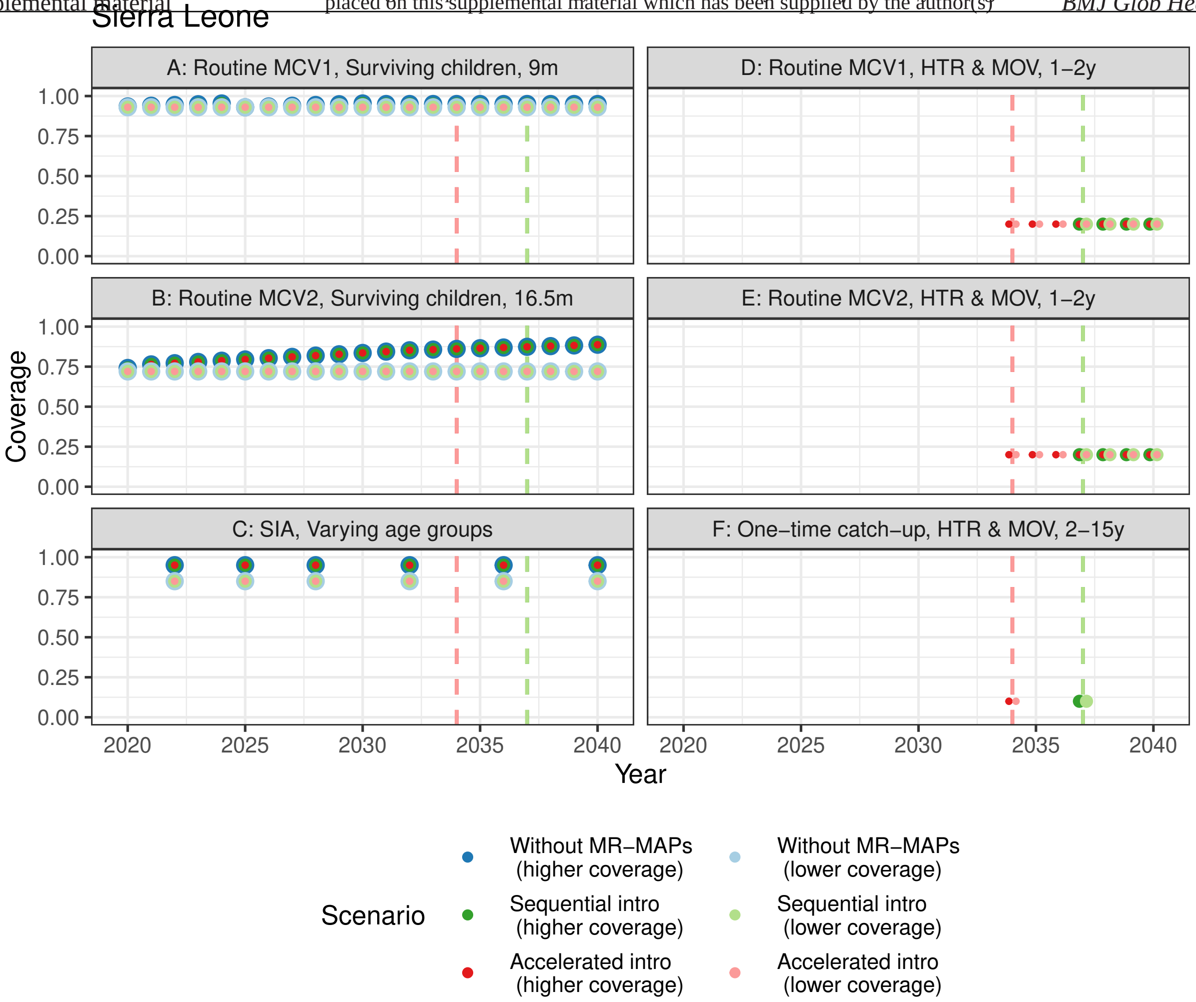
Sierra Leone
A: Routine MCV1, Surviving children, 9m
D: Routine MCV1, HTR & MOV, 1−2y
B: Routine MCV2, Surviving children, 16.5m
E: Routine MCV2, HTR & MOV, 1−2y
C: SIA, Varying age groups
F: One−time catch−up, HTR & MOV, 2−15y
Coverage
1.00
0.75
0.50
0.25
0.00
2020
2025
2030
2035
2040
Year
Scenario
Without MR−MAPs (higher coverage)
Without MR−MAPs (lower coverage)
Sequential intro (higher coverage)
Sequential intro (lower coverage)
Accelerated intro (higher coverage)
Accelerated intro (lower coverage)

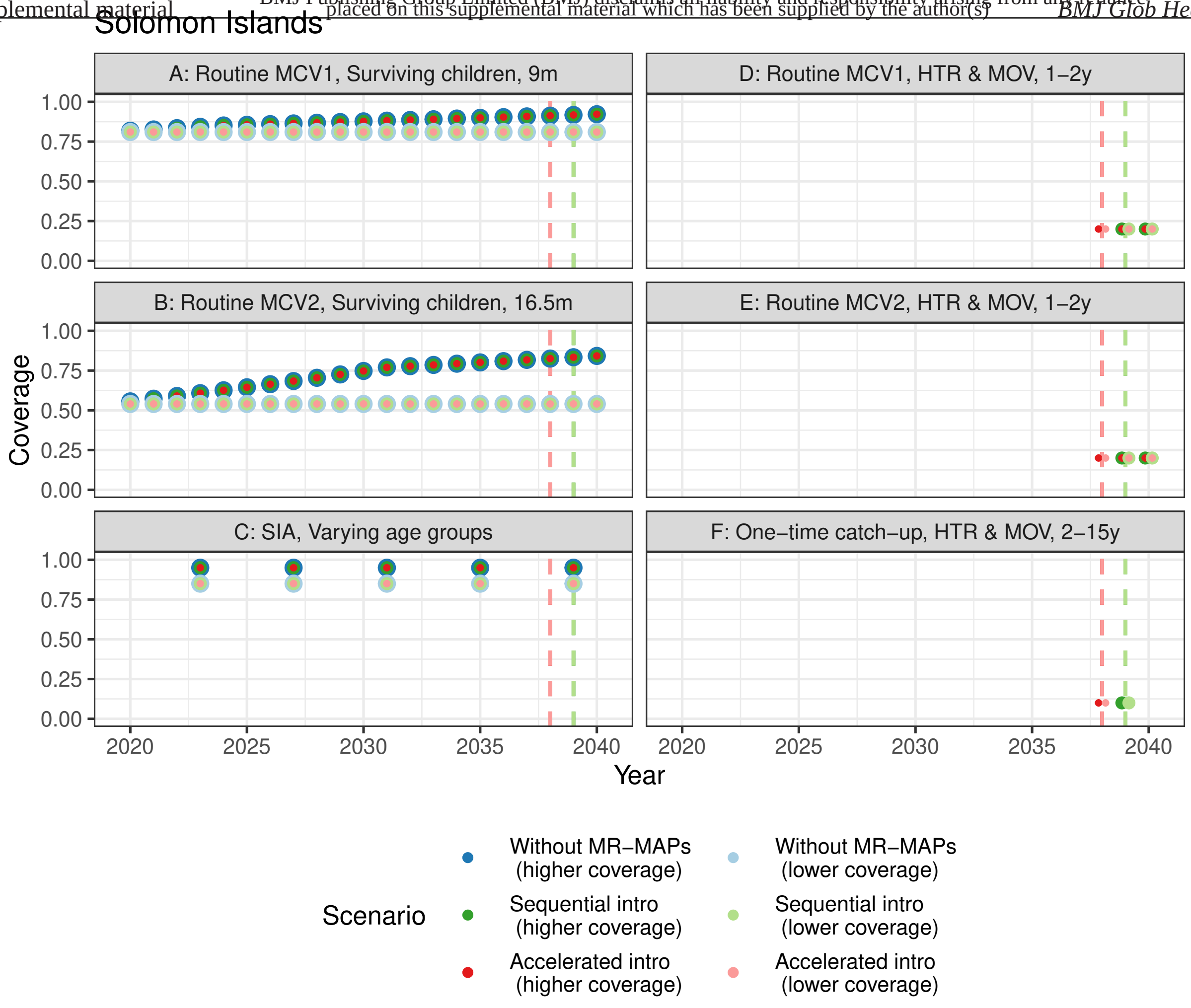
Solomon Islands
A: Routine MCV1, Surviving children, 9m
D: Routine MCV1, HTR & MOV, 1–2y
B: Routine MCV2, Surviving children, 16.5m
E: Routine MCV2, HTR & MOV, 1–2y
C: SIA, Varying age groups
F: One–time catch–up, HTR & MOV, 2–15y
Coverage
1.00
0.75
0.50
0.25
0.00
2020
2025
2030
2035
2040
Year
Scenario
Without MR–MAPs (higher coverage)
Without MR–MAPs (lower coverage)
Sequential intro (higher coverage)
Sequential intro (lower coverage)
Accelerated intro (higher coverage)
Accelerated intro (lower coverage)

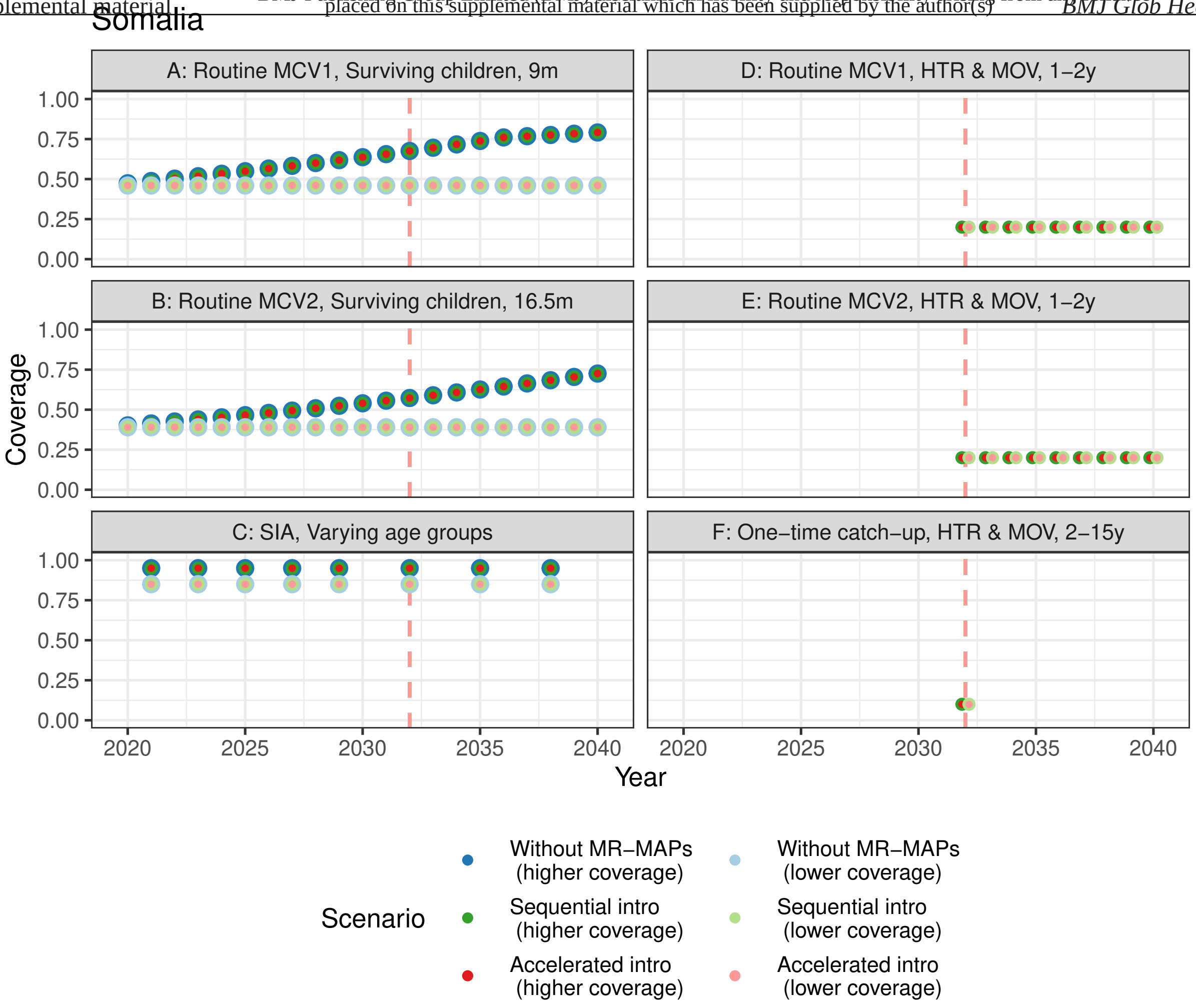
Somalia
A: Routine MCV1, Surviving children, 9m
B: Routine MCV2, Surviving children, 16.5m
C: SIA, Varying age groups
D: Routine MCV1, HTR & MOV, 1–2y
E: Routine MCV2, HTR & MOV, 1–2y
F: One–time catch–up, HTR & MOV, 2–15y
Coverage
1.00
0.75
0.50
0.25
0.00
2020
2025
2030
2035
2040
Year
Scenario
Without MR–MAPs (higher coverage)
Sequential intro (higher coverage)
Accelerated intro (higher coverage)
Without MR–MAPs (lower coverage)
Sequential intro (lower coverage)
Accelerated intro (lower coverage)

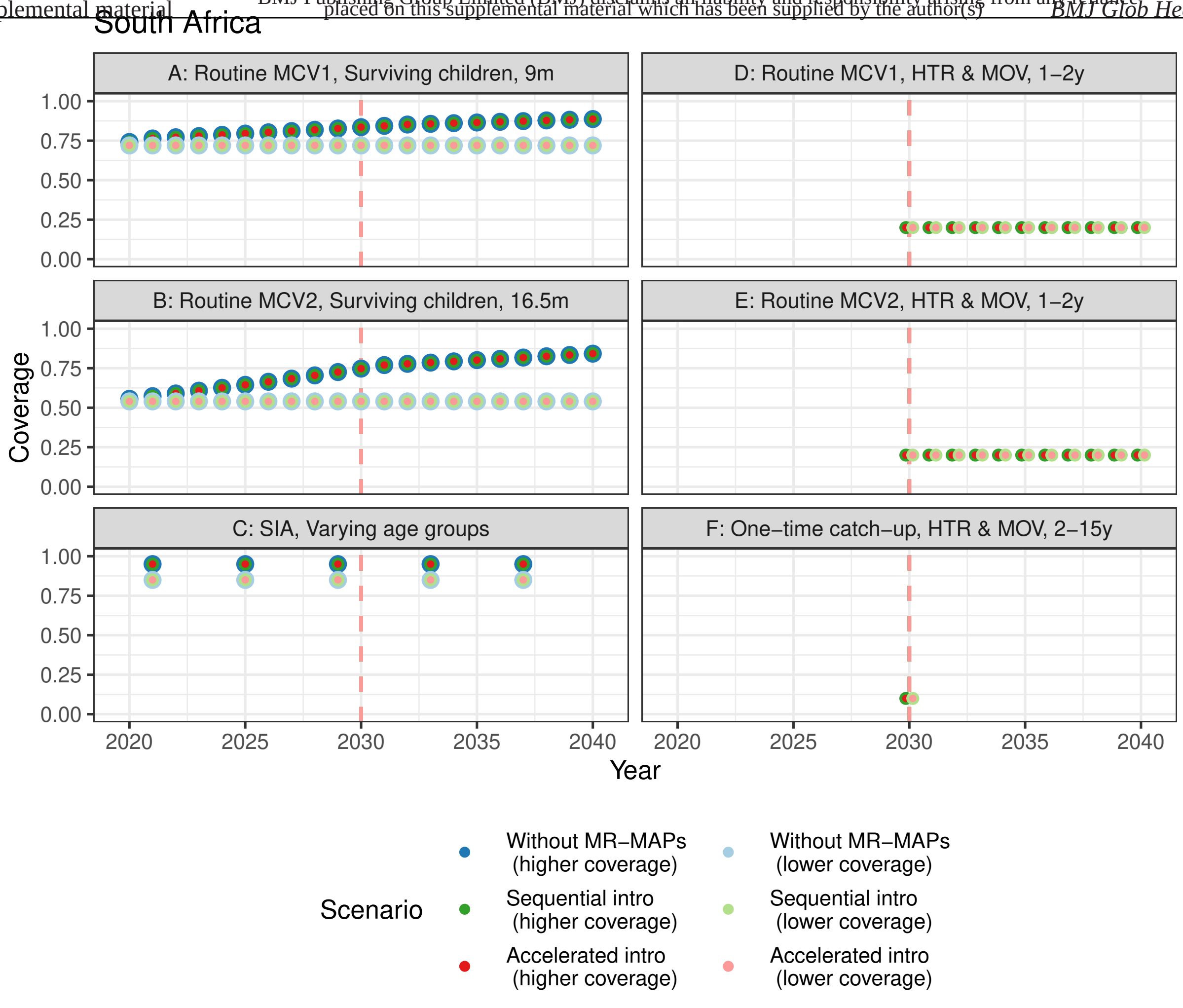
South Africa
A: Routine MCV1, Surviving children, 9m
D: Routine MCV1, HTR & MOV, 1–2y
B: Routine MCV2, Surviving children, 16.5m
E: Routine MCV2, HTR & MOV, 1–2y
C: SIA, Varying age groups
F: One−time catch−up, HTR & MOV, 2–15y
Coverage
1.00
0.75
0.50
0.25
0.00
2020
2025
2030
2035
2040
Year
Scenario
Without MR−MAPs (higher coverage)
Sequential intro (higher coverage)
Accelerated intro (higher coverage)
Without MR−MAPs (lower coverage)
Sequential intro (lower coverage)
Accelerated intro (lower coverage)

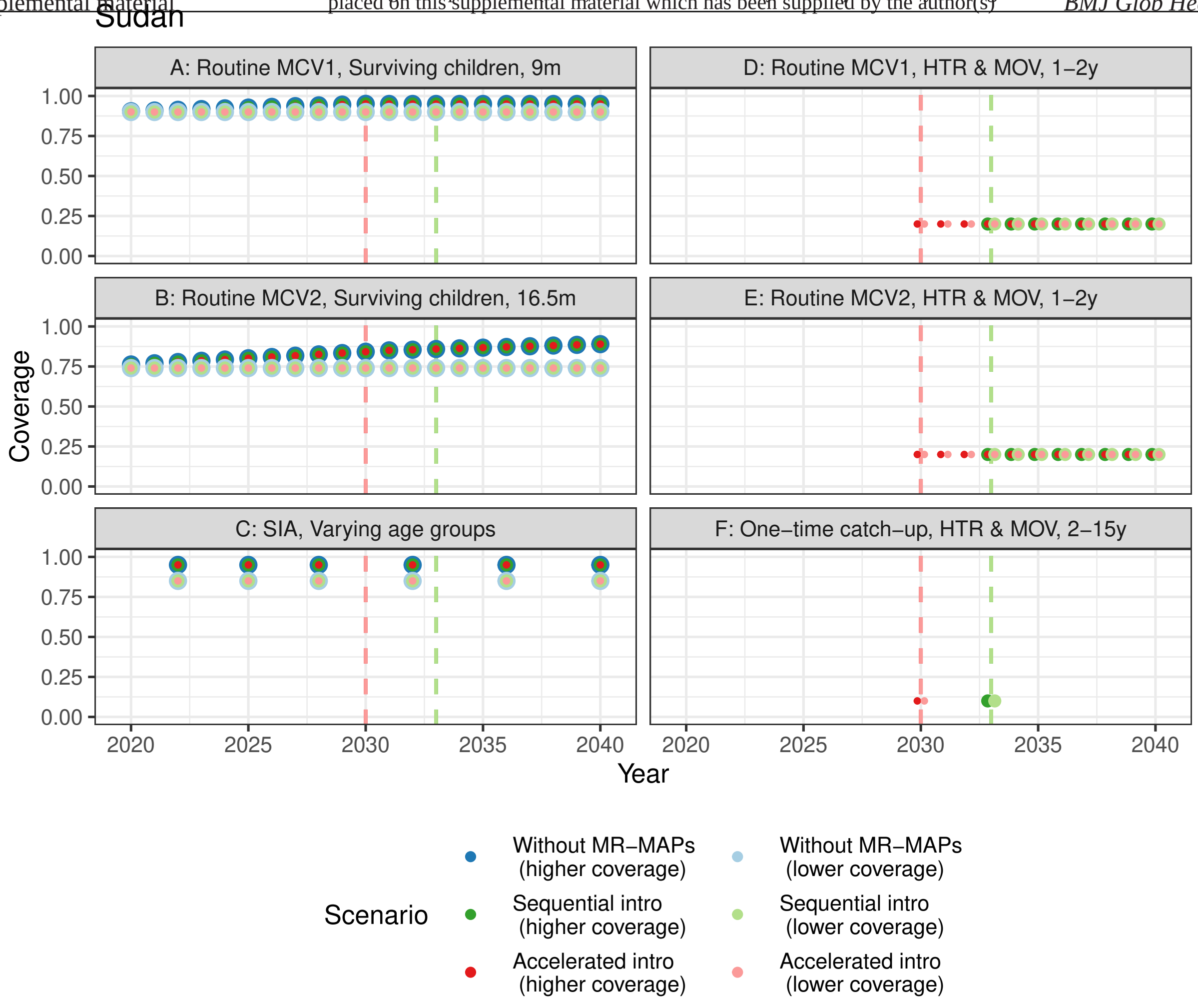
Sudan
A: Routine MCV1, Surviving children, 9m
D: Routine MCV1, HTR & MOV, 1−2y
B: Routine MCV2, Surviving children, 16.5m
E: Routine MCV2, HTR & MOV, 1−2y
C: SIA, Varying age groups
F: One−time catch−up, HTR & MOV, 2−15y
Coverage
1.00
0.75
0.50
0.25
0.00
2020
2025
2030
2035
2040
Year
Scenario
Without MR−MAPs (higher coverage)
Without MR−MAPs (lower coverage)
Sequential intro (higher coverage)
Sequential intro (lower coverage)
Accelerated intro (higher coverage)
Accelerated intro (lower coverage)

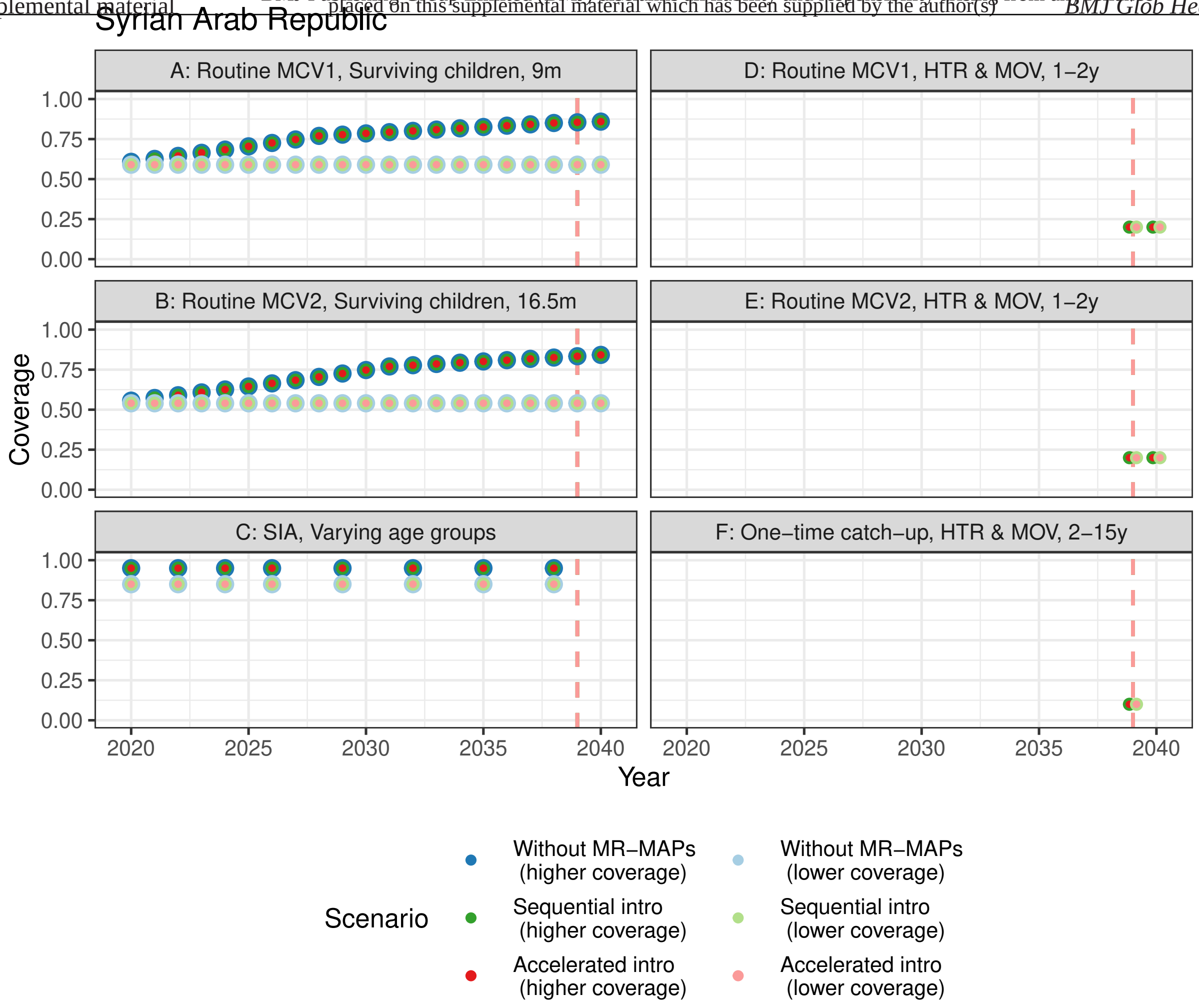
Syrian Arab Republic
A: Routine MCV1, Surviving children, 9m
D: Routine MCV1, HTR & MOV, 1−2y
B: Routine MCV2, Surviving children, 16.5m
E: Routine MCV2, HTR & MOV, 1−2y
C: SIA, Varying age groups
F: One−time catch−up, HTR & MOV, 2−15y
Coverage
1.00
0.75
0.50
0.25
0.00
2020
2025
2030
2035
2040
Year
Scenario
Without MR−MAPs (higher coverage)
Sequential intro (higher coverage)
Accelerated intro (higher coverage)
Without MR−MAPs (lower coverage)
Sequential intro (lower coverage)
Accelerated intro (lower coverage)

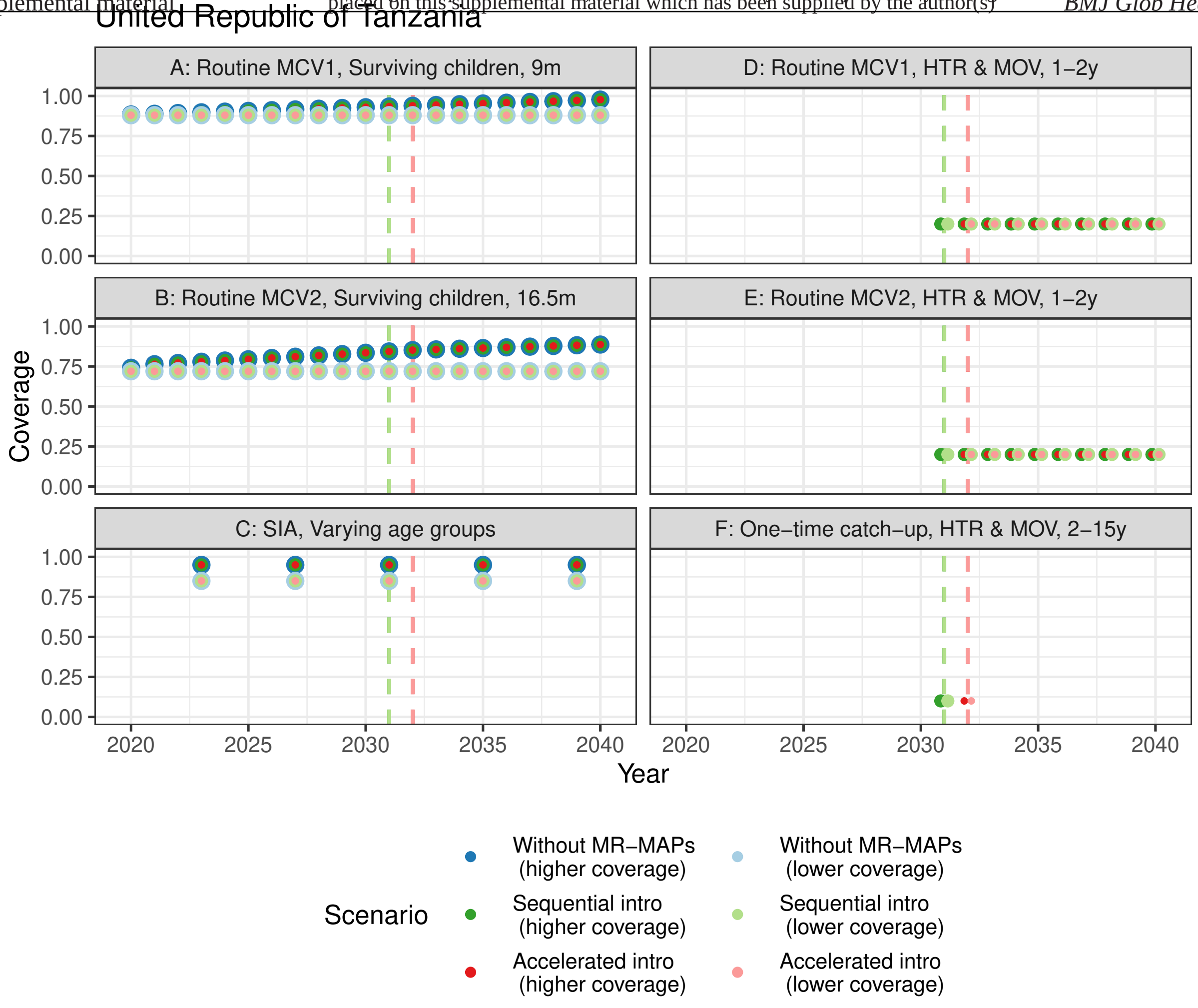
United Republic of Tanzania
A: Routine MCV1, Surviving children, 9m
D: Routine MCV1, HTR & MOV, 1−2y
B: Routine MCV2, Surviving children, 16.5m
E: Routine MCV2, HTR & MOV, 1−2y
C: SIA, Varying age groups
F: One−time catch−up, HTR & MOV, 2−15y
Coverage
1.00
0.75
0.50
0.25
0.00
2020
2025
2030
2035
2040
Year
Scenario
Without MR−MAPs (higher coverage)
Without MR−MAPs (lower coverage)
Sequential intro (higher coverage)
Sequential intro (lower coverage)
Accelerated intro (higher coverage)
Accelerated intro (lower coverage)

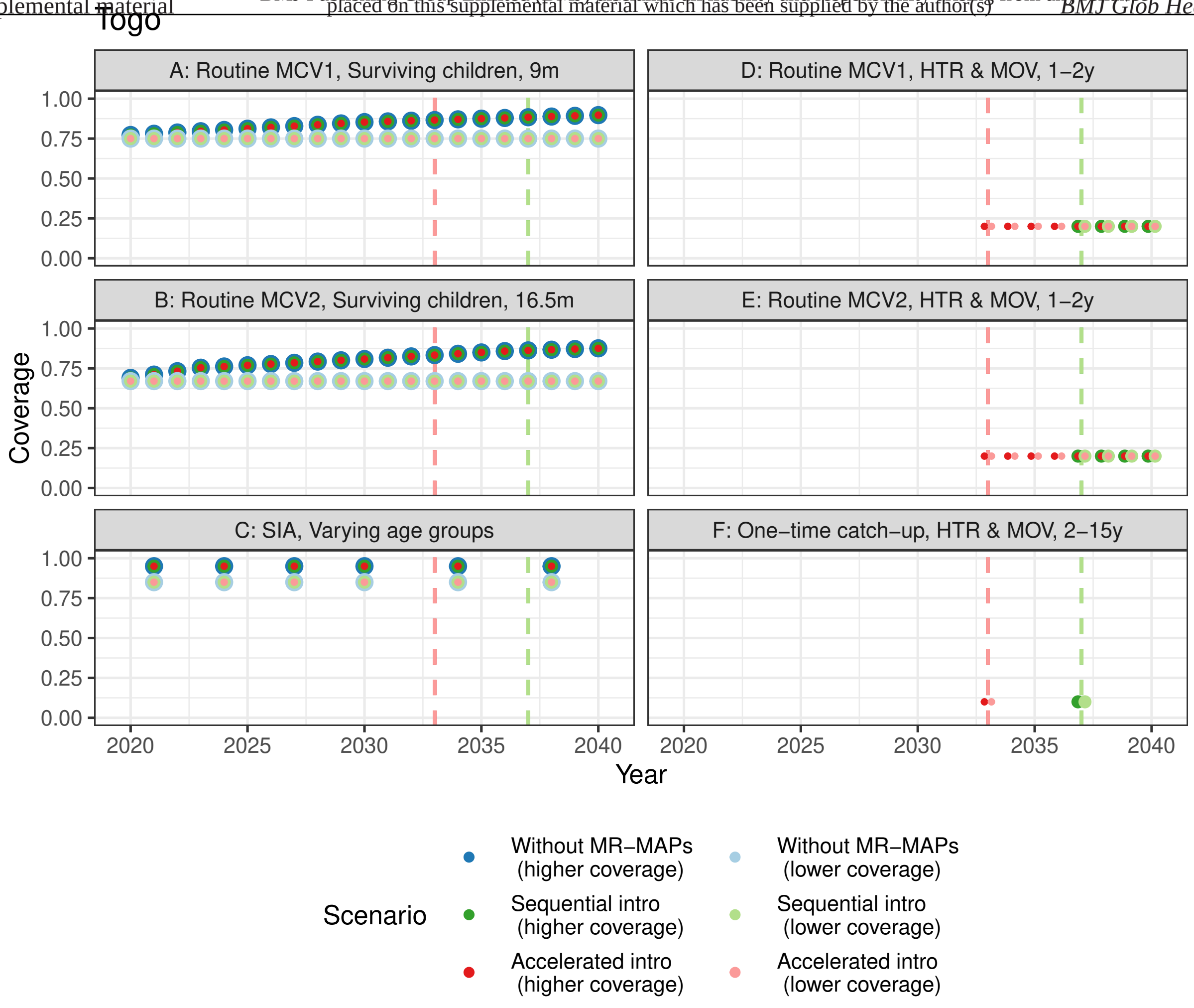
Togo
A: Routine MCV1, Surviving children, 9m
D: Routine MCV1, HTR & MOV, 1−2y
B: Routine MCV2, Surviving children, 16.5m
E: Routine MCV2, HTR & MOV, 1−2y
C: SIA, Varying age groups
F: One−time catch−up, HTR & MOV, 2−15y
Coverage
1.00
0.75
0.50
0.25
0.00
2020
2025
2030
2035
2040
Year
Scenario
Without MR−MAPs (higher coverage)
Sequential intro (higher coverage)
Accelerated intro (higher coverage)
Without MR−MAPs (lower coverage)
Sequential intro (lower coverage)
Accelerated intro (lower coverage)

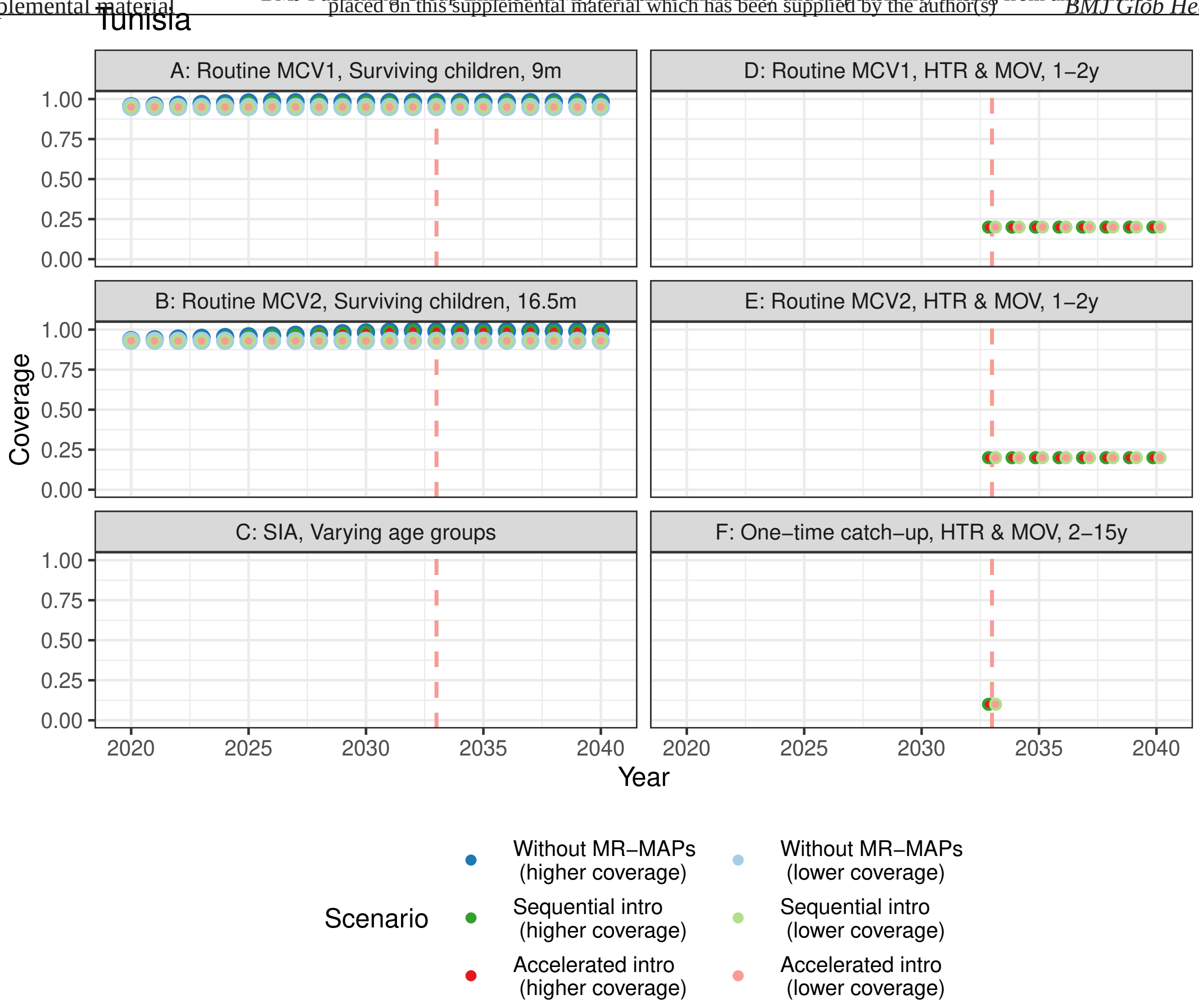
Tunisia
A: Routine MCV1, Surviving children, 9m
B: Routine MCV2, Surviving children, 16.5m
C: SIA, Varying age groups
D: Routine MCV1, HTR & MOV, 1–2y
E: Routine MCV2, HTR & MOV, 1–2y
F: One–time catch–up, HTR & MOV, 2–15y
Coverage
1.00
0.75
0.50
0.25
0.00
2020
2025
2030
2035
2040
Year
Scenario
Without MR–MAPs (higher coverage)
Without MR–MAPs (lower coverage)
Sequential intro (higher coverage)
Sequential intro (lower coverage)
Accelerated intro (higher coverage)
Accelerated intro (lower coverage)

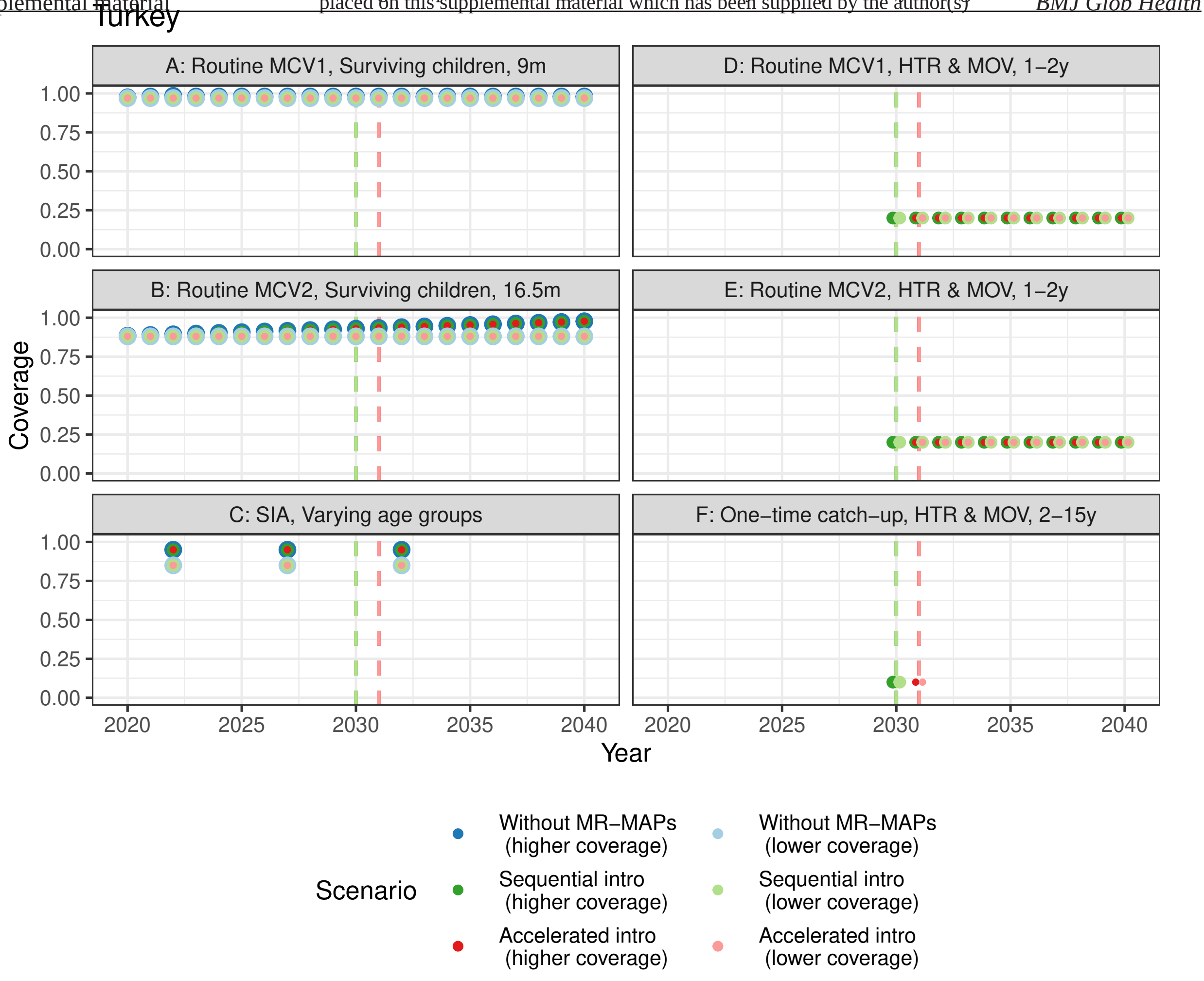
Turkey
A: Routine MCV1, Surviving children, 9m
D: Routine MCV1, HTR & MOV, 1−2y
B: Routine MCV2, Surviving children, 16.5m
E: Routine MCV2, HTR & MOV, 1−2y
C: SIA, Varying age groups
F: One−time catch−up, HTR & MOV, 2−15y
Coverage
1.00
0.75
0.50
0.25
0.00
2020
2025
2030
2035
2040
Year
Scenario
Without MR−MAPs (higher coverage)
Without MR−MAPs (lower coverage)
Sequential intro (higher coverage)
Sequential intro (lower coverage)
Accelerated intro (higher coverage)
Accelerated intro (lower coverage)

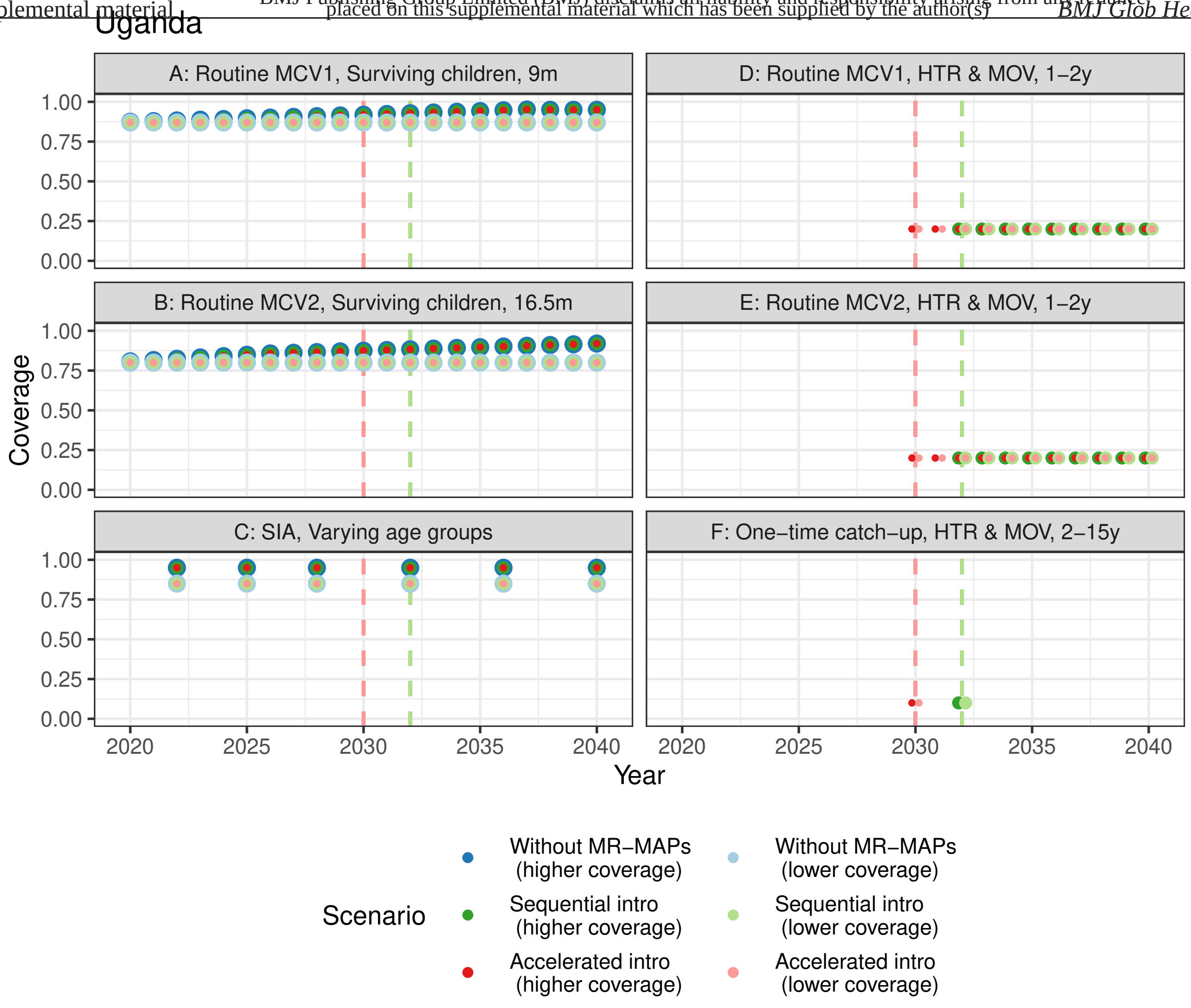
Uganda
A: Routine MCV1, Surviving children, 9m
D: Routine MCV1, HTR & MOV, 1−2y
B: Routine MCV2, Surviving children, 16.5m
E: Routine MCV2, HTR & MOV, 1−2y
C: SIA, Varying age groups
F: One−time catch−up, HTR & MOV, 2−15y
Coverage
1.00
0.75
0.50
0.25
0.00
2020
2025
2030
2035
2040
Year
Scenario
Without MR−MAPs (higher coverage)
Without MR−MAPs (lower coverage)
Sequential intro (higher coverage)
Sequential intro (lower coverage)
Accelerated intro (higher coverage)
Accelerated intro (lower coverage)

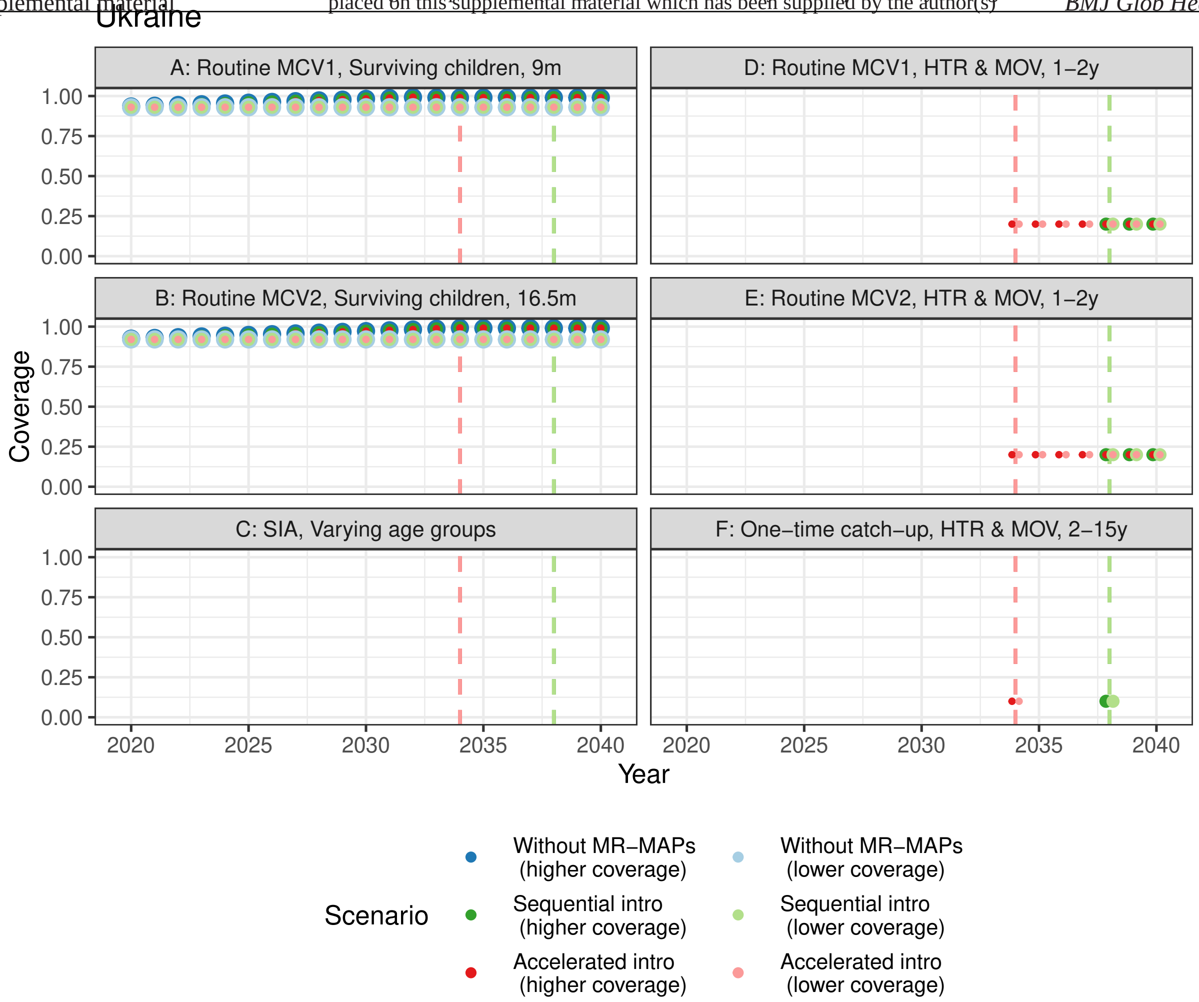
Ukraine
A: Routine MCV1, Surviving children, 9m
D: Routine MCV1, HTR & MOV, 1–2y
B: Routine MCV2, Surviving children, 16.5m
E: Routine MCV2, HTR & MOV, 1–2y
C: SIA, Varying age groups
F: One–time catch–up, HTR & MOV, 2–15y
Coverage
1.00
0.75
0.50
0.25
0.00
2020
2025
2030
2035
2040
Year
Scenario
Without MR–MAPs (higher coverage)
Without MR–MAPs (lower coverage)
Sequential intro (higher coverage)
Sequential intro (lower coverage)
Accelerated intro (higher coverage)
Accelerated intro (lower coverage)

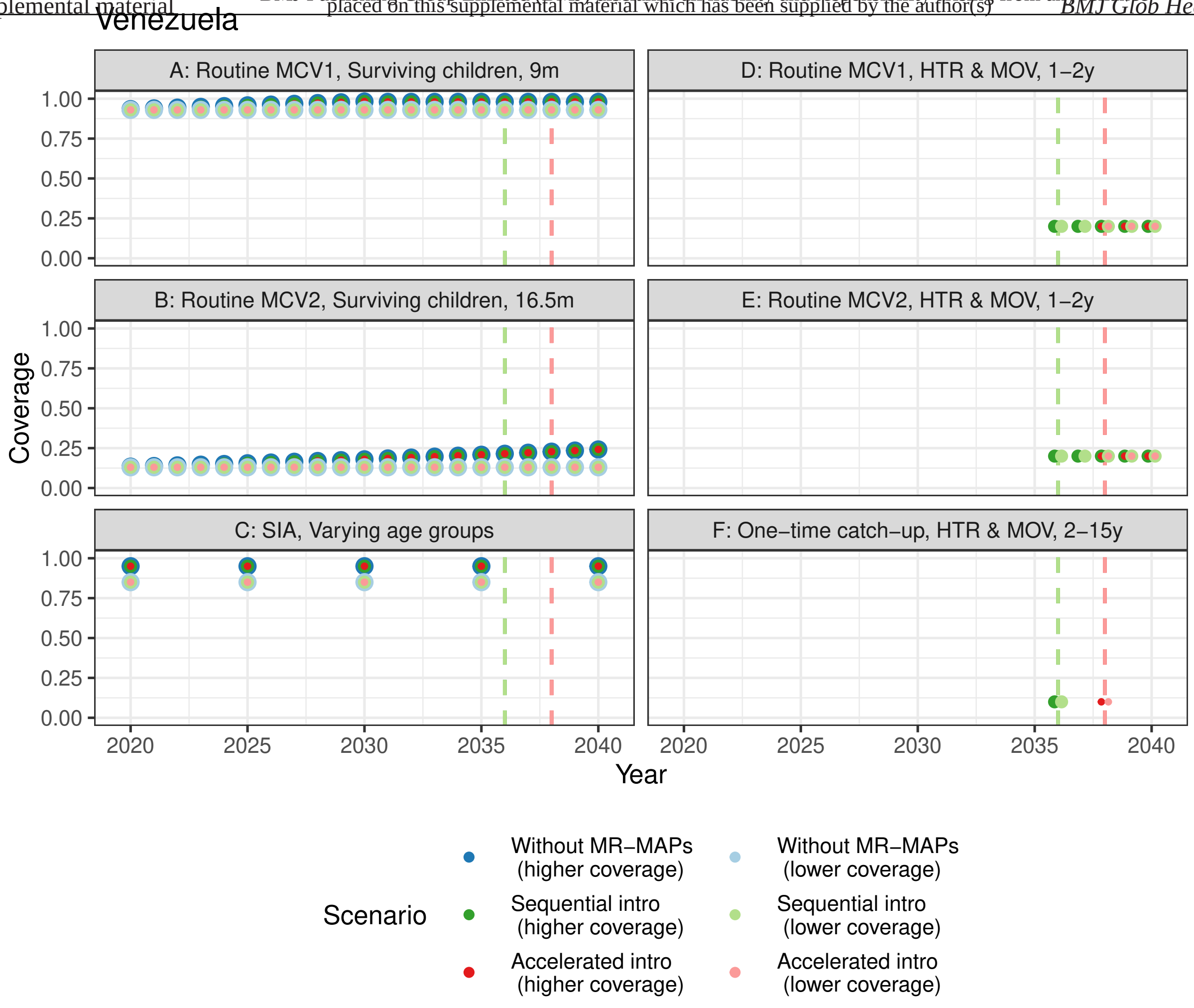
Venezuela
A: Routine MCV1, Surviving children, 9m
D: Routine MCV1, HTR & MOV, 1−2y
B: Routine MCV2, Surviving children, 16.5m
E: Routine MCV2, HTR & MOV, 1−2y
C: SIA, Varying age groups
F: One−time catch−up, HTR & MOV, 2−15y
Coverage
1.00
0.75
0.50
0.25
0.00
Year
2020
2025
2030
2035
2040
Scenario
Without MR−MAPs (higher coverage)
Without MR−MAPs (lower coverage)
Sequential intro (higher coverage)
Sequential intro (lower coverage)
Accelerated intro (higher coverage)
Accelerated intro (lower coverage)

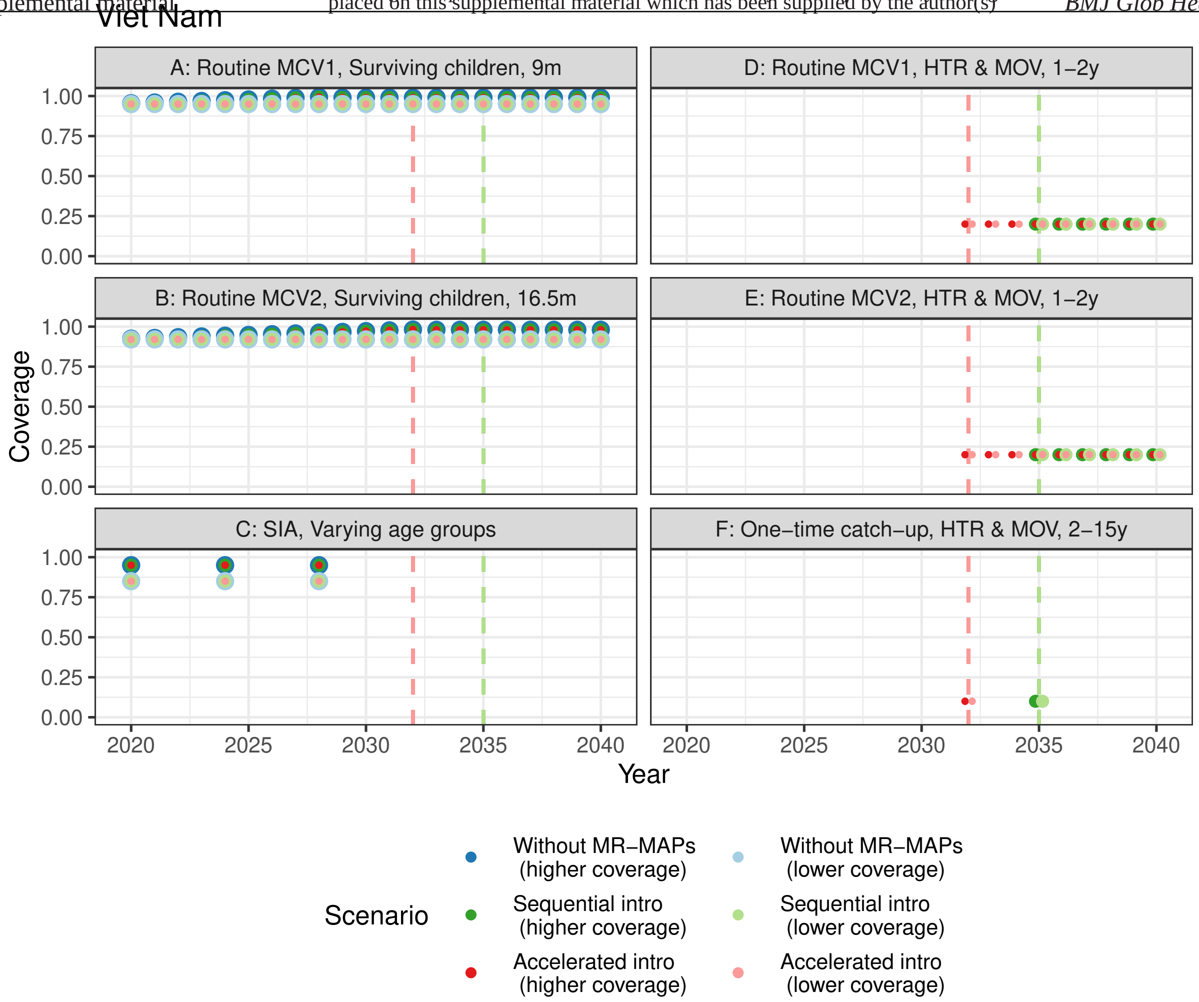
Viet Nam
A: Routine MCV1, Surviving children, 9m
D: Routine MCV1, HTR & MOV, 1−2y
B: Routine MCV2, Surviving children, 16.5m
E: Routine MCV2, HTR & MOV, 1−2y
C: SIA, Varying age groups
F: One−time catch−up, HTR & MOV, 2−15y
Coverage
1.00
0.75
0.50
0.25
0.00
2020
2025
2030
2035
2040
Year
Scenario
Without MR−MAPs (higher coverage)
Without MR−MAPs (lower coverage)
Sequential intro (higher coverage)
Sequential intro (lower coverage)
Accelerated intro (higher coverage)
Accelerated intro (lower coverage)

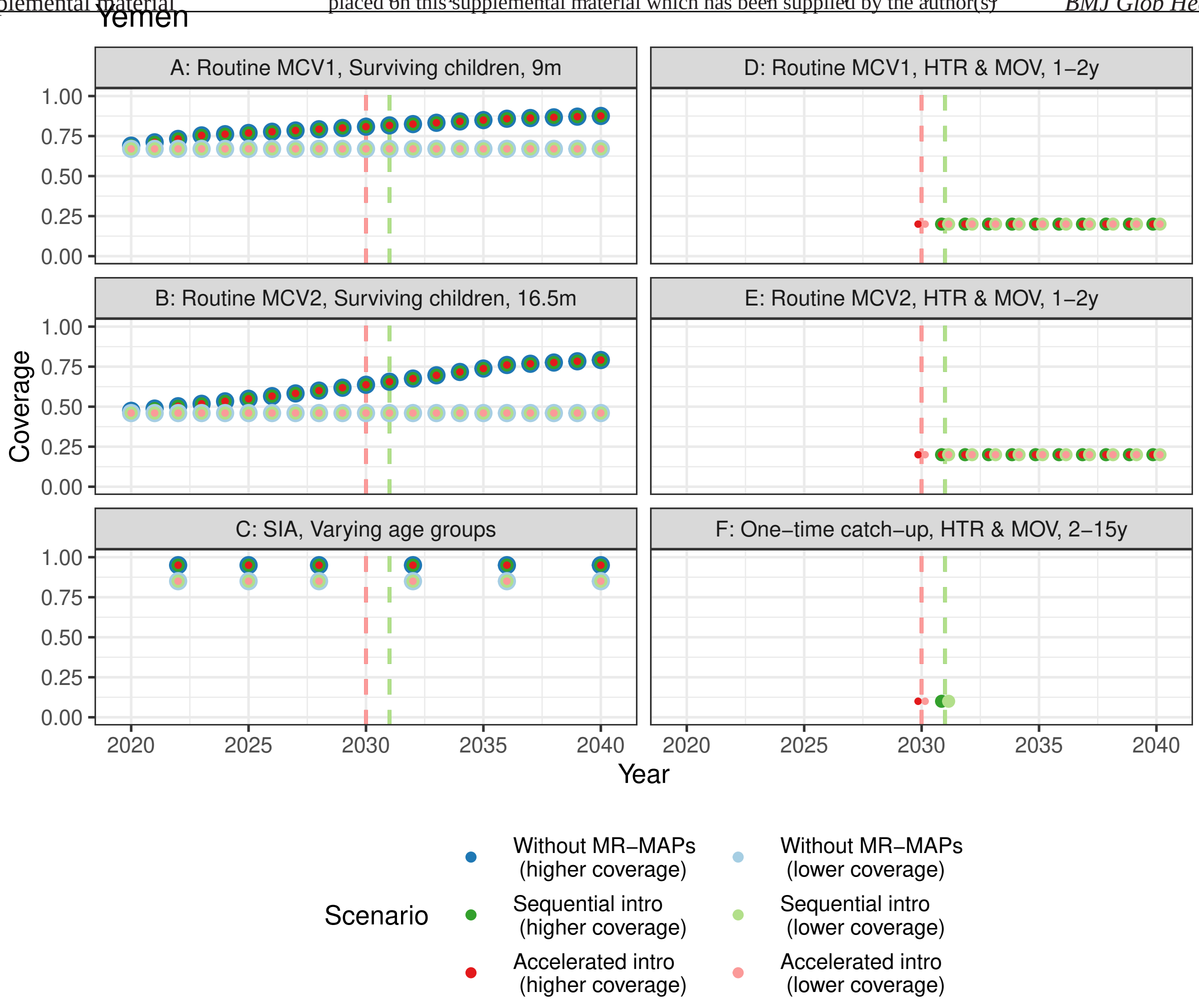
Yemen
A: Routine MCV1, Surviving children, 9m
D: Routine MCV1, HTR & MOV, 1−2y
B: Routine MCV2, Surviving children, 16.5m
E: Routine MCV2, HTR & MOV, 1−2y
C: SIA, Varying age groups
F: One−time catch−up, HTR & MOV, 2−15y
Coverage
1.00
0.75
0.50
0.25
0.00
2020
2025
2030
2035
2040
Year
Scenario
Without MR−MAPs (higher coverage)
Without MR−MAPs (lower coverage)
Sequential intro (higher coverage)
Sequential intro (lower coverage)
Accelerated intro (higher coverage)
Accelerated intro (lower coverage)

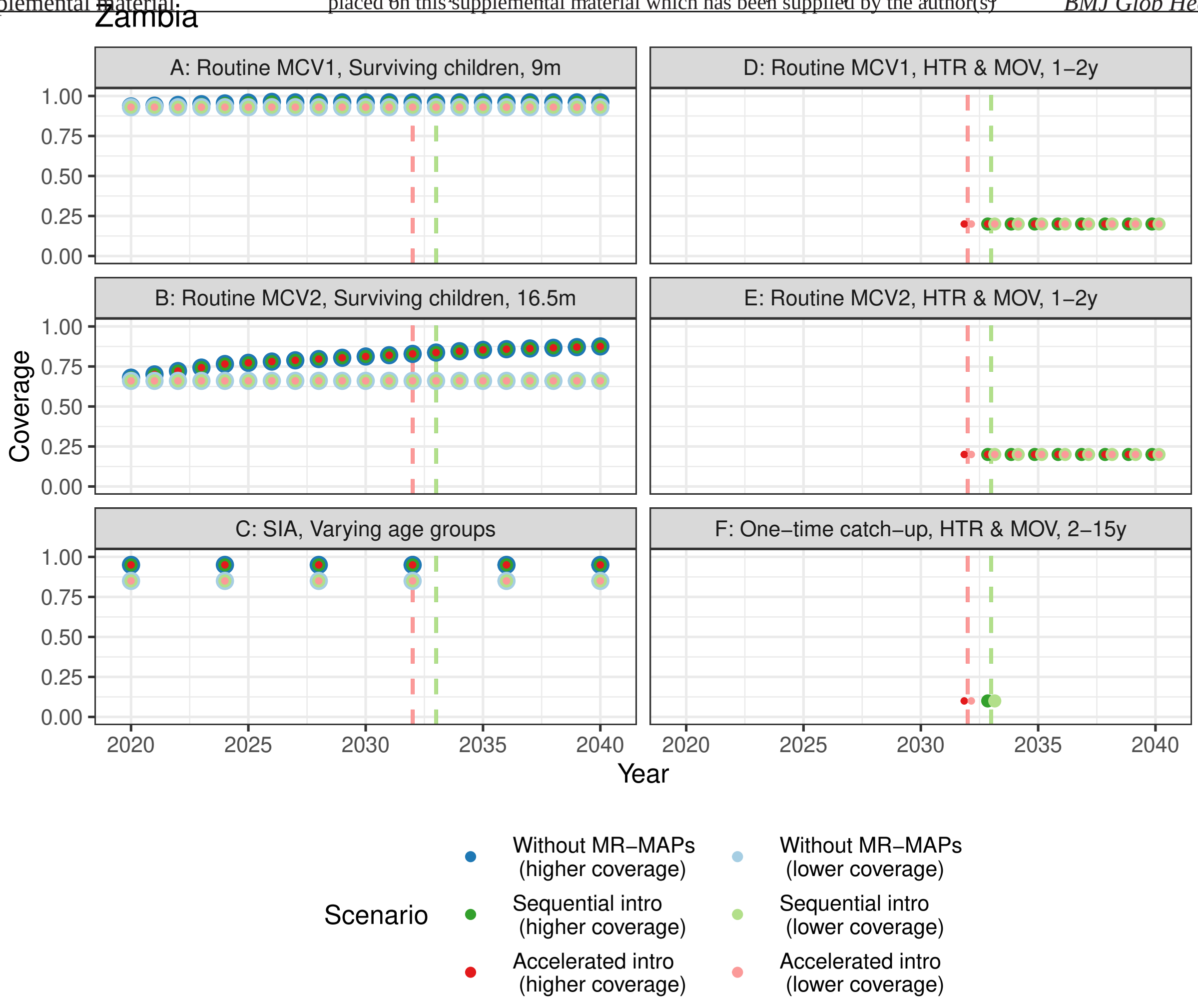
Zambia
A: Routine MCV1, Surviving children, 9m
D: Routine MCV1, HTR & MOV, 1−2y
B: Routine MCV2, Surviving children, 16.5m
E: Routine MCV2, HTR & MOV, 1−2y
C: SIA, Varying age groups
F: One−time catch−up, HTR & MOV, 2−15y
Coverage
1.00
0.75
0.50
0.25
0.00
2020
2025
2030
2035
2040
Year
Scenario
Without MR−MAPs (higher coverage)
Without MR−MAPs (lower coverage)
Sequential intro (higher coverage)
Sequential intro (lower coverage)
Accelerated intro (higher coverage)
Accelerated intro (lower coverage)

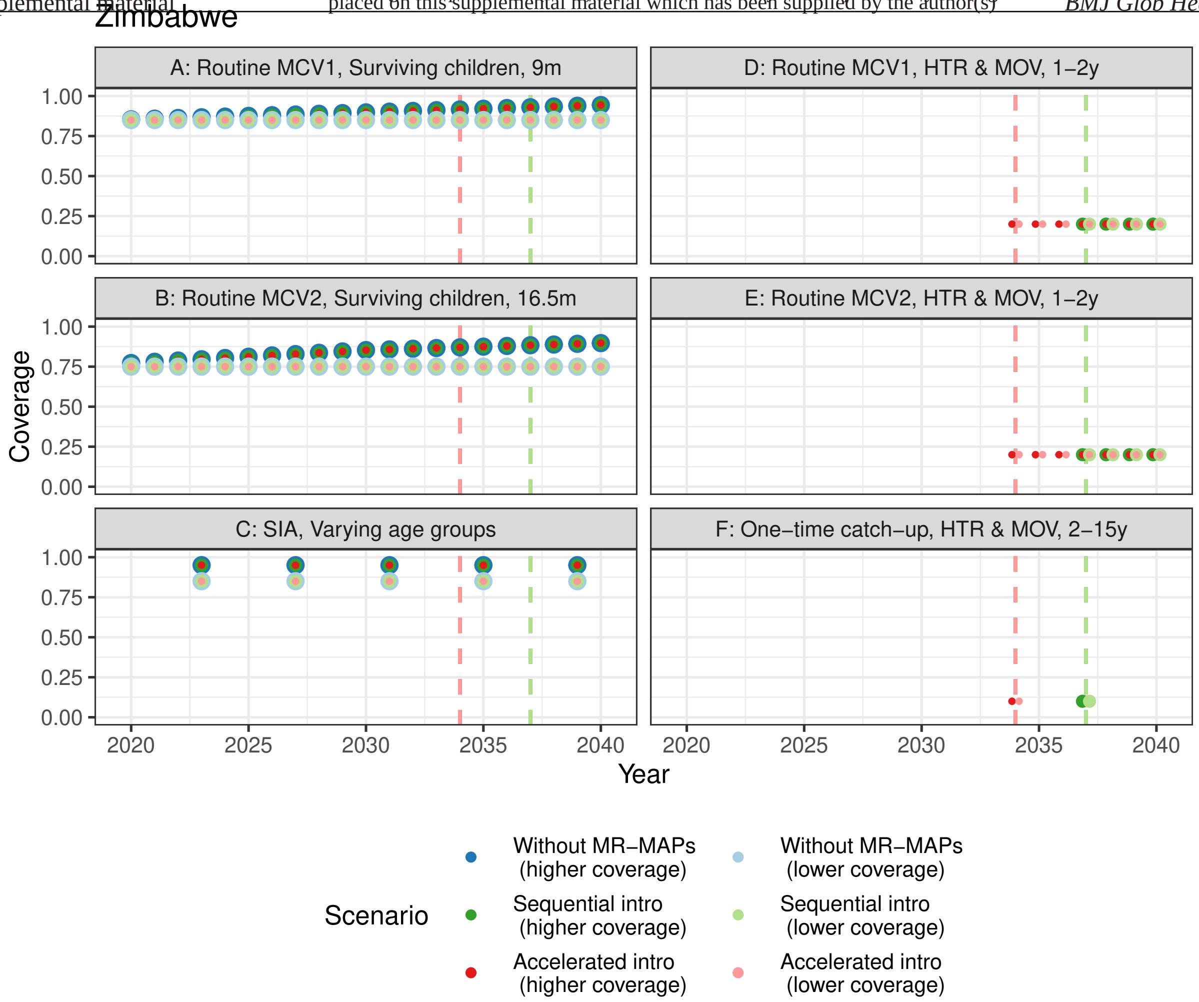
Zimbabwe
A: Routine MCV1, Surviving children, 9m
D: Routine MCV1, HTR & MOV, 1−2y
B: Routine MCV2, Surviving children, 16.5m
E: Routine MCV2, HTR & MOV, 1−2y
C: SIA, Varying age groups
F: One−time catch−up, HTR & MOV, 2−15y
Coverage
1.00
0.75
0.50
0.25
0.00
2020
2025
2030
2035
2040
Year
Scenario
Without MR−MAPs (higher coverage)
Without MR−MAPs (lower coverage)
Sequential intro (higher coverage)
Sequential intro (lower coverage)
Accelerated intro (higher coverage)
Accelerated intro (lower coverage)
